# Membrane Trafficking Modulation during *Entamoeba* Encystation

**DOI:** 10.1038/s41598-017-12875-6

**Published:** 2017-10-09

**Authors:** Emily Herman, Maria A. Siegesmund, Michael J. Bottery, Ronny van Aerle, Maulood Mohammed Shather, Elisabet Caler, Joel B. Dacks, Mark van der Giezen

**Affiliations:** 1 Department of Cell Biology, Faculty of Medicine and Dentistry, University of Alberta, T6G 2H7 Edmonton, Alberta Canada; 2 Biosciences, University of Exeter, Stocker Road, Exeter, EX4 4QD UK; 3 Department of Biology, University of York, Heslington, York, YO10 5DD UK; 4 Centre for Environment, Fisheries, and Aquaculture Science (Cefas), Barrack Road, The Nothe, Weymouth, Dorset, DT4 8UB UK; 5 J. Craig Venter Institute, 9714 Medical Center Drive, Rockville, MD 20850 USA; 6 Present Address: National Heart, Lung, and Blood Institute (NHLBI), National Institutes of Health (NIH), 6701, Rockledge Drive, Room 9144, Bethesda, MD 20892-7950 USA

## Abstract

*Entamoeba histolytica* is an intestinal parasite that infects 50–100 million people and causes up to 55,000 deaths annually. The transmissive form of *E*. *histolytica* is the cyst, with a single infected individual passing up to 45 million cysts per day, making cyst production an attractive target for infection control. Lectins and chitin are secreted to form the cyst wall, although little is known about the underlying membrane trafficking processes supporting encystation. As *E*. *histolytica* does not readily form cysts *in vitro*, we assessed membrane trafficking gene expression during encystation in the closely related model *Entamoeba invadens*. Genes involved in secretion are up-regulated during cyst formation, as are some *trans*-Golgi network-to-endosome trafficking genes. Furthermore, endocytic and general trafficking genes are up-regulated in the mature cyst, potentially preserved as mRNA in preparation for excystation. Two divergent dynamin-related proteins found in *Entamoeba* are predominantly expressed during cyst formation. Phylogenetic analyses indicate that they are paralogous to, but quite distinct from, classical dynamins found in human, suggesting that they may be potential drug targets to block encystation. The membrane-trafficking machinery is clearly regulated during encystation, providing an additional facet to understanding this crucial parasitic process.

## Introduction

*Entamoeba histolytica* is one of the most common causes of death due to parasites in the world^1^. It is thought to infect 50–100 million people each year of whom 55,500 succumb due to dysentery, amoebic colitis and invasive disease such as amoebic liver abscess^2,3^. Although a global pathogen, it is a major issue in tropical areas within the Indian subcontinent, Africa and South and Central America^4^. The majority of *E*. *histolytica* infections are asymptomatic cyst spreaders, but 10% of infections will develop disease^3^, with symptoms including dysentery, colitis, and amoebic granulomas^5^. If arteries are perforated during infection, amoebae can spread to the liver and brain causing abscesses, to the heart causing pericarditis, and to the lungs causing pleuropulmonary disease^5^. Invasive infection and dysentery is commonly treated with nitroimidazole-derived drugs^6^ and the aminoglycoside paromomycin, but surgical intervention may be necessary in cases of fulminant colitis^7,8^.

*E*. *histolytica* parasites are transmitted by ingestion of cyst-contaminated water. While the average infectious dose is >1,000 organisms, a single infectious individual can pass up to 45 million cysts in their stool per day^9^. After exiting the host, cysts must survive the drying, nutrient poor, and thermo-variable conditions of the environment for several weeks to months. Once taken up, cysts face the harsh acidity of the stomach and the majority of the small intestine, rich with digestive enzymes, as excystation occurs later in the terminal ileum^10^. After excystation, the *Entamoeba* trophozoite divides and colonises the colon, in some cases causing tremendous tissue destruction. It is here that trophozoites form the hardy and infectious cysts that are excreted to the environment^5^.

The robustness and prodigious quantity of cysts shed by infected individuals means that communities in the developing nations that lack proper sanitation are particularly affected^4^. As a diarrheal disease, *Entamoeba* infection influences child mortality, development, and worker productivity, promoting poverty^11^. In regions with unreliable water treatment and waste disposal infrastructure, and where access to medicines for treatment and symptom management may be insufficient, controlling the spread of the environmental cyst form of *Entamoeba* is of utmost importance. Several groups have sought to elucidate the systems and molecules that are involved in encystation, recently reviewed in Mi-ichi *et al*. (2016) including Gal-lectin production, catecholamine pathway signalling, cholesterol sulphate synthesis, heat shock protein 90, chitin metabolism, proteolytic systems, and potentially enolase^12^. However, little is known about the underlying cellular trafficking events that facilitate encystation, and furthermore, how the quiescent cyst is prepared for eventual excystation.

The process of forming a stable, resistant cyst relies on the specific, ordered secretion of cyst wall proteins. It is thought that the cyst wall is assembled in three phases, according to the “wattle-and-daub” model^13^. In the foundation stage, the lectin Jacob is trafficked to the cell surface and there binds constitutively expressed Gal/GalNAc lectins^13,14^. During the “wattle” stage, chitin is synthesized and secreted, likely crosslinked by Jacob’s tandemly arranged chitin-binding domains. Jacob lectins and chitin are seen in separate vesicles in early encystation (12 hours post induction, hpi), and have begun to accumulate at the cyst wall at 24–36 hpi^13^. Although the timing is not clear, the chitin-cleaving enzyme chitinase and deacetylases trim and deacetylate extracellular chitin^15,16^. Finally, the Jessie3 lectin, which binds chitin and may also self-aggregate, solidifies the cyst wall in the “daub” phase, making it impermeable to small molecules^13^. Jessie3 is observed in vesicles beginning at 36 hpi, and is found in the cyst wall at 72 hpi^13^. The heavy secretory load clearly implicates the membrane trafficking system (MTS) in encystation.

Membrane trafficking is the movement of proteins and other macromolecules throughout the organelles of the endomembrane system, including endo- and exocytosis. It is indeed a defining feature of eukaryotic cells, and has been shown to be critical for parasite infection^17^ and evasion of the immune system^18^. Several studies have implicated the MTS in *Entamoeba* parasitism^19–21^, and specifically encystation. Membrane trafficking events are regulated by small GTPases such as Rabs, involved with vesicle fusion dynamics, and Arfs, which regulate the assembly of protein coats on vesicles. Fourteen Rab genes were found to be upregulated during encystation by a microarray screen^22^. A targeted bioinformatics analysis has shown that *Entamoeba* has expanded its Rab protein family through gene duplication; it has over 100 Rab proteins^23^, many of which lack homologues in human^24^, raising the possibility that some divergent MTS proteins may be potential targets for drugs abrogating cyst formation. Furthermore, the *E*. *histolytica* genome paper reported that the amoebae contain basic vesicle transport machinery common to eukaryotic cells, and in addition to Rabs, it has also expanded its Arf GTPase complement^25^. However, an in-depth assessment of the *Entamoeba* MTS, and how it relates to encystation, has not yet been done. Due to its importance in cyst formation, we have taken a comparative genomics and transcriptomics approach to define the MTS gene complement of *Entamoeba*, and then assessed how these genes are modulated during encystation. A recent transcriptomic analysis of *E*. *histolytica* pathogenesis relative to its environment highlights the relevance of large-scale genomic approaches to understanding multiple aspects of this parasite’s biology and lifestyle^26^.

One membrane trafficking factor potentially involved in cyst formation in *E*. *histolytica* is the dynamin family of enzymes. Dynamins are large GTPase proteins involved in membrane remodelling^27,28^, although the exact mechanism by which dynamin drives membrane fission is still debated^29^. In general, dynamins first polymerise around the neck of a vesicle forming a helix, and then induce scission through tube constriction and/or membrane destabilization, by hydrolysing GTP. They are essential for myriad cellular processes, such as endocytosis, organelle division, cytokinesis, vesicle scission, and cytoskeletal organisation^29,30^. In a previous study of *Giardia intestinalis*, an unrelated enteric cyst-forming parasite, its single dynamin-related protein (Drp) was shown to be necessary in cyst formation^31^. It is associated with encystation-specific vesicles, and expression of a dominant negative Drp mutant blocks *Giardia* encystation^31^. Because of the involvement of dynamin in membrane trafficking, and the requirement of Drp function for *Giardia* stage conversion, we focused particularly on the expression of dynamin-related proteins during *Entamoeba* encystation.

As *E*. *histolytica* cysts do not form readily *in vitro*, the closely related model *Entamoeba invadens* is often used to study cyst formation. *E*. *invadens* is a reptile pathogen, and encystation is easily induced under axenic conditions by glucose starvation^32^. We induced encystation in *E*. *invadens* and performed RNA-Seq on mRNA from the trophozoite (0 hpi), early encystation stage (24 hpi), late encystation stage (48 hpi), and the mature cyst (72 hpi), and identified MTS gene expression patterns during this process. In addition to showing the up-regulation of previously identified encystation genes such as Jacob, Jessie and chitinase, we found that the secretory pathway is generally up-regulated during cyst formation, as well as TGN-endosome trafficking. Furthermore, we found that the mature cyst contains specific mRNAs associated with clathrin-mediated endocytosis, potentially in preparation for excystation. Two highly divergent Drps are also implicated by their predominant expression and by their changed localization during encystation.

## Results

### Transcriptome assembly

We induced encystation in *E*. *invadens* and performed transcriptomics in trophozoites, during early and late encystation, and in the mature cyst. Transcriptome sequencing resulted in 40 to 160 million reads per time point, with the fewest being at 48 hpi (late stage encystation) and the highest being at 0 hpi (trophozoite). After pre-processing to trim adapters and filter poor-quality reads, the number of reads were 34.3 million reads at 0 hpi, 27.8 million reads at 24 hpi, 9.1 million reads at 48 hpi, and 26.4 million reads at 72 hpi. These reads were mapped to the *E*. *invadens* IP-1 genome with PASA identifying 116 potential new transcripts. The *E*. *invadens* genome contains 11,553 predicted proteins, and only 473 have expression values of 0 FPKM at all time points. Therefore, approximately 96% of the *E*. *invadens* genome is transcribed at least at one time point during encystation. Of the 116 novel transcripts, only 26 retrieved any other sequence when used to search the NCBI non-redundant nucleotide and protein databases, and 11 of these are related to transposable element sequences (Supplementary Table S1). All genes are listed with expression values in Supplementary Table S1.

### *E*. *histolytica* and *E*. *invadens* have a similar repertoire of MTS genes

**Figure 1:**
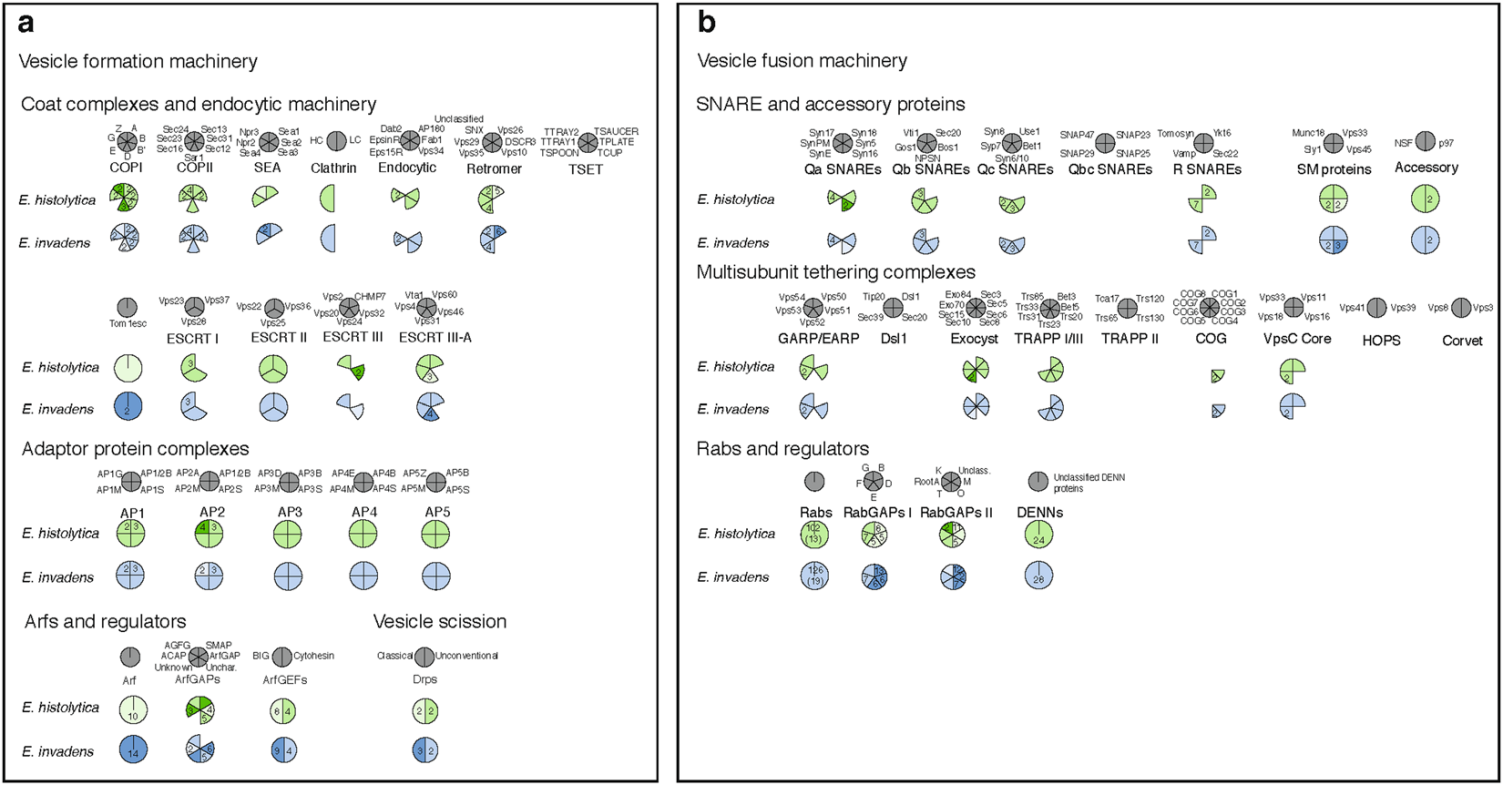
Coulson plot showing vesicle formation machinery (**a**) and vesicle fusion machinery (**b**) in *E*. *histolytica* and *E*. *invadens*. Filled sectors indicate presence of a homologue while empty sectors indicate that it could not be identified. Numbers indicate paralogues. Sectors are shaded lighter or darker to indicate where the two *Entamoeba* species differ in copy number.

In order to support the validity *E*. *invadens* as a model to study the MTS during encystation, we used comparative genomics to assess the complement of MTS genes in *E*. *histolytica* and *E*. *invadens*. The predicted proteomes of *E*. *invadens* and *E*. *histolytica*, and the *E*. *invadens* transcriptome generated in this study, were examined for the presence of MTS genes using molecular data from human and other model organisms using the homology searching tool BLASTP. Figure 1a and b show the presence and paralogue number of vesicle formation and vesicle fusion machinery, respectively, in the two *Entamoeba* genomes. Orthologue accession numbers for all MTS components searched are available in Supplementary Table S2. In general, the presence/absence of MTS genes is conserved between the two species, with 246 of 367 identified genes showing one to one orthology. Excluding Arf and Rab GTPases and their regulators, there are 11 genes for which *E*. *invadens* and *E*. *histolytica* have a different number of paralogues, and in most of these cases, only one or two duplications have occurred.

The Arf and Rab GTPases, the TBC (Tre-2/Bub2/Cdc16) proteins (Rab GTPase Activating Proteins; Rab GAPs), and the DENN domain-containing proteins (Guanine nucleotide Exchange Factors for Rabs; RabGEFs) are highly paralogous gene families. Phylogenetic analyses of Arf and Rab GTPases and their regulators were undertaken in order to determine 1:1 orthologues and independent expansions within *Entamoeba*. Supplementary Table S3 is a summary of this work, with the corresponding phylogenies found in Supplementary Figures S1M–N, S1R-T, S2A-B, and Supplementary Files 3 and 4. With the exception of the Arfs, more than half of these proteins have direct 1:1 orthology between *E*. *invadens* and *E*. *histolytica*, while less than 1/3 of the proteins are ‘singletons’ (i.e. one or more gene family members in one *Entamoeba* species with no clear orthologues in the other). The rest of the proteins make up orthologous groups with some expansion or loss events (i.e. 1 + :1 or 1:1 + orthology). Overall, the vast majority of GTPases and their regulators share orthology between the two species (>75%), although there have been multiple independent expansions in both species. While there is divergence in this one sub-system of the membrane-trafficking machinery between the two organisms, the overall similarity of the remaining MTS complement in *E*. *histolytica* and *E*. *invadens* is high. 130 of 155 genes have 1:1 orthology, and there are 61 cases where a factor deduced as present in the LECA was not identified in either genome. Therefore, we suggest that *E*. *invadens* is a good proxy for studying encystation in the human pathogen *E*. *histolytica*.

The vesicle formation machinery (Fig. 1a) is largely complete relative to that of human and yeast model systems, despite the high sequence divergence of *Entamoeba* spp. genes. The only complex that appears to be absent given its complete lack of identified subunits is TSET, a recently discovered vesicle coat complex that has been partially or fully lost numerous times in eukaryotes^33^. Although other complexes show partial losses, it is not clear whether these are real gene absences or false negatives due to high sequence divergence, and generally enough members of the complex are present that it may still function even if some subunits are truly missing. There has been a clear *Entamoeba* lineage-specific duplication of the coat complex COPI, which is responsible for retrograde transport in the Golgi, and between the Golgi and ER^34^. There has also been a notable expansion of the Vps26 subunit of the retromer complex in both species, which plays a role in cargo preference for the complex in yeast and human^35,36^. Retromer is responsible for the recycling of lysosomal cargo receptors from the endosomes to the TGN in yeast and human^37^, and in *Entamoeba*, it has been shown to be targeted to pre-phagosomal vacuoles (PPVs), an *Entamoeba-*specific endolysosomal organelle important for pathogenesis^21^. Finally, there are duplications in the large subunits of the adaptor protein (AP) complexes 1 and 2, which interact with the vesicle coat proteins in TGN-endosome and endocytic trafficking, respectively^38,39^.

Common losses are observed in both *Entamoeba* species for the vesicle fusion machinery (Fig. 1b). Anterograde trafficking from the ER to the Golgi is supported by the presence of SNARE proteins Syntaxin 5, Bos1, Ykt6^40^, ^*inter*, *alia*^ and potentially Syntaxin of plants 7 (Syp7)^41^, as well as the SM protein Sly1 and the majority of the multi-subunit tethering complex (MTC) TRAPPI^42^. Conversely, neither the SNARE proteins associated with retrograde Golgi-ER trafficking, nor the MTC Dsl1, appear to be present. Only two subunits were identified of the Conserved Oligomeric Golgi (COG) complex, an MTC involved in retrograde Golgi trafficking^43,44^. However, as the COPI coat complex is present (and certain subunits duplicated), and Golgi-ER retrograde trafficking is an integral feature of ER resident protein recycling and cisternal maturation^34,45–47^, it is likely that this trafficking pathway is functional.

Genes involved in endosome-to-TGN trafficking are conserved and in some cases expanded; for example, Syntaxin 16, Vti1, Syntaxins 6/10, and Vesicle Associated Membrane Proteins (VAMPs)^40^, ^*inter*, *alia*^ the Sec. 1/Munc18-like protein Vps45^48^, and a partial GARP MTC complex^49^. This mirrors the expansions of the retromer subunit Vps26, as retromer is the coat complex that functions in this pathway. The endosome-to-lysosome trafficking pathway appears to be somewhat incomplete, as the SNAREs syntaxin 7 and syntaxin 8 could not be confidently identified, and while three out of four VpsC core components were found, we could not identify any of the HOPS (Vps41, Vps39) or Corvet (Vps3, Vps8) accessory proteins.

Two secretory SNARE subfamilies have also undergone expansion. While the SNARE proteins involved in constitutive versus regulated secretion are not well established outside of mammals^50^, there is evidence for an expansion of plasma membrane syntaxins, as well as VAMPs, which can function at the plasma membrane. The exocytic SM protein Munc18 was identified^51^, as well as a partial, but potentially functional, exocyst complex; the MTC involved in exocytosis^52^. This expansion certainly raises the possibility that extra machinery may support secretion of cyst components. In order to determine which MTS proteins are specifically involved in this process, we assessed their expression patterns during encystation.

### Genes differentially expressed during encystation fall into ten clusters of expression patterns

Statistical pair-wise comparisons of each time point were conducted to identify significantly differently expressed genes using edgeR^53^. This resulted in a set of 9,073 genes identified as differentially expressed between at least two time points, of which 4,987 were considered to be significant (defined as FDR < 0.01, fold change >2). As the dispersion between genes was set *a priori* rather than estimated from the data, we chose not to focus on differential expression of individual genes. We opted instead to cluster genes based on their expression patterns, and then identify the membrane trafficking pathways for which a clear pattern is observed.

**Figure 2:**
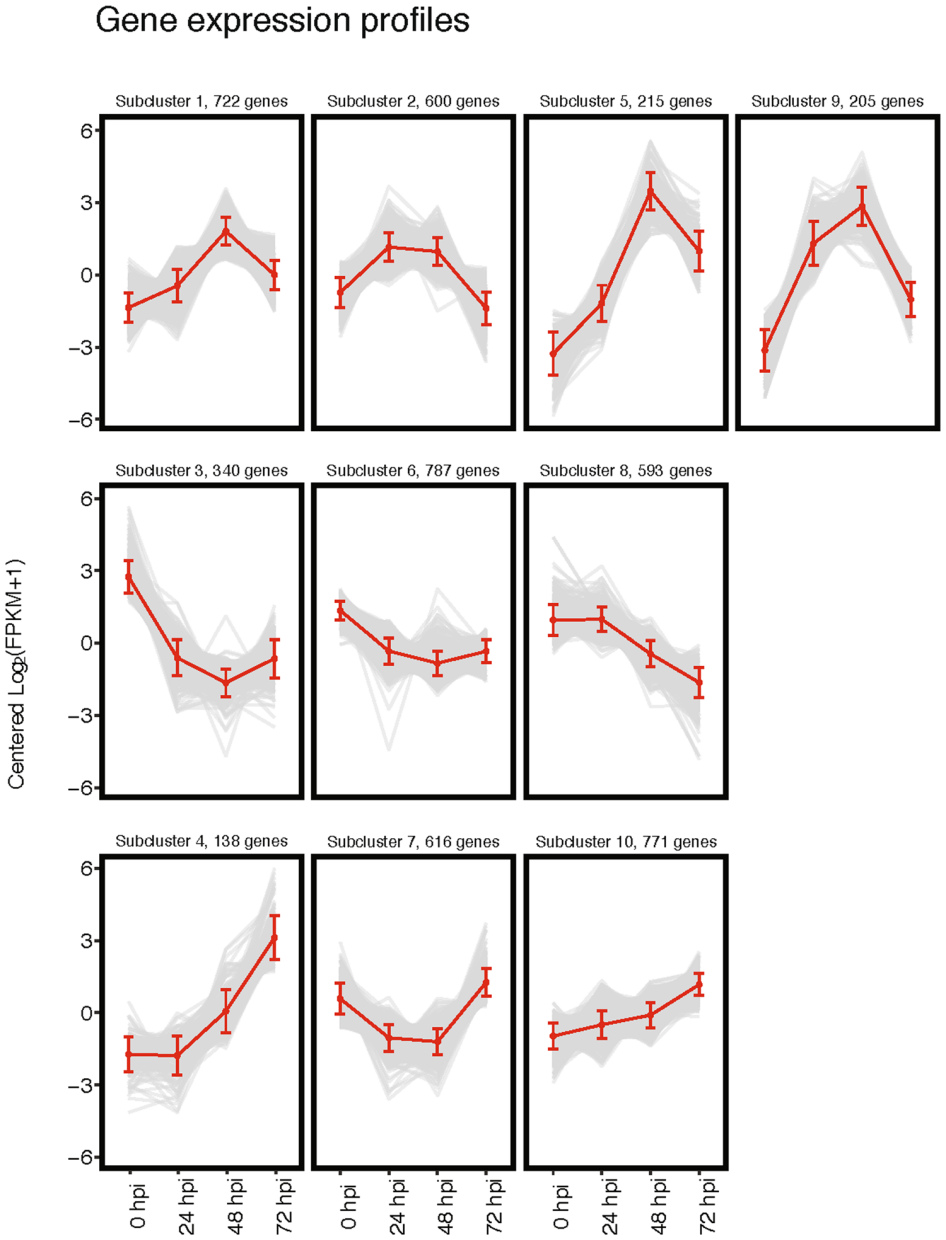
Differentially expressed transcripts cluster into ten expression profiles. Plots of expression profiles (centred log_2_ FPKM) of transcripts that are significantly differently expressed at least one time point (FDR < 0.01 log_2_ fold change >2) split into ten subclusters defined using both *k*-means and MCL clustering algorithms. The number of genes in each cluster is provided above respective plots. Red points represent the cluster’s mean expression profile.

Ten clusters with elevated expression at one or more time point were identified using *k*-means clustering (Fig. 2). In general, the subclusters represent three over-arching patterns: genes whose expression increases during early and/or late encystation, but decreases in the mature cyst; genes whose expression decreases during encystation; and genes whose expression increases in the mature cyst. The first pattern – genes up-regulated during encystation – includes subclusters 1, 2, 5, and 9. Within this group, it is possible to further distinguish genes whose expression remains constant in both early (24 hpi) and late encystation (48 hpi, subcluster 2) from those whose expression peaks in late encystation (subclusters 1, 5, and 9). Subclusters 3 and 8 are characterised by a general decrease in expression during the encystation process, including in the mature cyst (72 hpi). Finally, genes whose expression peaks in the mature cyst make up subclusters 4, 7, and 10. Subcluster 7 is particularly intriguing, as its members are generally down-regulated during early and late encystation, but return to expression levels equivalent to or slightly higher than in the trophozoite (0 hpi). Subcluster 10, on the other hand, shows a pattern of steadily increasing expression throughout encystation and in the mature cyst. Subcluster 6 shows a pattern of downregulation between the trophozoite stage and early encystation (24 hpi); however, the magnitude of this change is low relative to the other expression patterns, and therefore we have chosen to focus on the other subclusters. Jacob lectins are found in subcluster 9, except EiJacob6 and EiJacob7 which are found in subclusters 4 and 5, respectively. Jessie lectins are found in subcluster 5. This is congruent with the notion that the Jacob proteins act at an earlier stage, as subcluster 9 describes genes that have high expression during early and late encystation, and Jessie proteins act later, as subcluster 5 describes genes specifically up-regulated in late encystation. Furthermore, all four chitinases have expression patterns in agreement with previous RT-PCR work^54^ and are also members of subclusters 5 and 9.

The variance between these clusters can be accounted for within the first two principle components of the data set (Supplementary Figure S3). The majority of the variance is explained by principle component 1, accounting for 58.4% of the total variability within the data set. This component is dominated by the contrast in expression of genes within trophozoite (0 hpi) and within late encystation (48 hpi). Principle component 2 accounts for 33% of the variance within the data set, representing the contrast in expression of genes between the encystation (24 hpi) and cyst (72 hpi) stages. Differences within expression between trophozoite (0 hpi) and within late encystation (48 hpi) play a prominent role in distinguishing the expression of these clusters.

### Membrane trafficking gene expression during encystation

Of approximately 400 MTS genes, 223 were identified as differentially expressed and could be clustered into the groups described above during encystation (Supplementary Table S1). Strikingly, we found that 1/4 of the ~400 trafficking components searched for in *E*. *invadens* encode multiple paralogues where two or more are found in subclusters with different general expression patterns. These findings point to a complex MTS with potentially functionally distinct paralogues that can be swapped in and out of protein complexes to finely tune trafficking processes during encystation. Rather than a blanket increase or decrease in expression of some trafficking factors over others, this suggests that the MTS is highly modulated and a critical part of encystation.

We identified 18 genes involved in secretion in subclusters 1, 2, 5, or 9, which show a pattern of up-regulation during encystation, and which do not have paralogues with opposing expression patterns (Supplementary Table S2). Several members of the COPII vesicle coat complex, responsible for the initial ER-to-Golgi step of trafficking secreted material, are up-regulated during encystation (Sar1^55^ and two paralogues of Sec. 24). Intra-Golgi and Golgi-ER retrograde trafficking is also represented in these subclusters, which include two of the six subunits of the COPI vesicle coat complex required for intra-Golgi and Golgi-ER trafficking, four BIG-like ArfGEF paralogues, as well as several SNARE proteins that function in this pathway (Syntaxin 5, Gos1, Bos1, Bet1) and the SM protein Sly1, which helps disassemble this SNARE complex. Post-Golgi secretory SNAREs are members of these subclusters: Syntaxin PM (two paralogues), and Syp7 as well as the cognate SM protein Munc18. Syp7 is a plant syntaxin thought to be involved in secretion in plants^41^, and due to its absence in human, it may represent a therapeutic target to disrupt encystation.

Rab1 and EhRabA, thought to function in early secretion^56,57^, appear to be down-regulated during encystation. However, they may play multiple roles in trafficking: while the *Entamoeba* Rab1 homologue has not been functionally characterized, Rab1 proteins in *Dictyostelium* cells are recruited to phagosomes^58^, and EhRabA has been shown to be involved in cell motility^59^.

The TGN-endosome recycling pathway is more modestly represented in subclusters 1, 2, 5, and 9, with five members. These include the SNARE protein Syntaxin 16, its cognate SM protein Vps45, EpsinR, and Fab1 (PIKfyve in mammals), which as well as a subunit of the GARP multi-subunit tethering complex, Vps51, is involved in the tethering of vesicles during fusion.

Overall, there is a weak endocytic/phagocytic signal in subclusters 3 and 8 (five members), whose expression decreases during encystation. These include both paralogues of Rab8, two cytohesin-like ArfGEFs, and Rab5. Contrary to this, the phagocytic Rab EhRabB appears to be up-regulated during encystation. However, this gene is known to be activated by heat shock^60^, and may be activated as part of the stress response cascade that has been proposed to regulate encystation^61^, rather than as part of an increase in phagocytic machinery. The *E*. *invadens* genome has many genes with multiple paralogues, and it is often the case that while one paralogue of a gene appears to be down-regulated during encystation (in subcluster 3 or 8), the other paralogue does not change in expression. As such, there are few genes and pathways that clearly exemplify a pattern of downregulation. Despite the high modulation and differential expression of MTS components, it is relatively rare for two paralogues of any given gene to have ‘opposing’ expression patterns, i.e. one paralogue is a member of a subcluster whose expression increases during encystation, and the other is part of a subcluster whose expression decreases under those same conditions. The majority of these cases occur in highly paralogous gene families, such as the TBC-B family of RabGAPs, or the exocytic SNARE protein Vamp7. Given the extensive duplications and diversification in these gene families, we are hesitant to use their subcluster membership as evidence of an emphasized MTS pathway.

Curiously, there are 57 MTS genes whose expression increases from late encystation (48 hpi) to the mature cyst (72 hpi; subclusters 4, 7, 10). The most obvious example is the ESCRT complex proteins, where 7 of 17 are found in these subclusters. The ESCRT complexes are responsible for multivesicular body biogenesis, a mechanism by which plasma membrane and cytosolic proteins can be targeted to the lysosome for degradation. Other members of these subclusters that are involved in endosome recycling and endo-lysosomal function include the adaptor protein complex AP1γ subunit (two paralogues), EpsinR, Syntaxin 6 or 10, two putative DENN2 paralogues, Rab7, TBC-B, and TBC-F. Clathrin heavy chain is also highly expressed in the mature cyst. There are 7 secretory pathway genes that make up these subclusters, including two members of the COPII coat complex (Sec. 23 and Sec. 31), two non-human SNARE proteins thought to function in ER-to-plasma membrane trafficking in plants (NPSN11 and Syp7), a plasma membrane syntaxin, the COPI subunit COPB’, and two BIG-like ArfGEFs. Finally, there are three ArfGAP1, one SMAP, and three uncharacterized ArfGAP paralogues in these subclusters, although their interacting Arf proteins and therefore functions are unknown. Other small G proteins and their regulators that could not be classified, or whose function is unknown, include five RabGEFs, three RabGAPs, two Arf proteins, and nine Rabs. These mRNAs are highly expressed in the mature cyst, although it is not clear whether they are translated into protein products at this stage. As the mature cyst is quiescent, it is possible that this is done to prepare for excystation, so that the cell can quickly perform early endocytic, exocytic and recycling functions prior to beginning the gene expression program seen in trophozoites. This may be analogous to increases in mRNA stability seen in late stage trophozoites of the malaria parasite *Plasmodium falciparum*, which has been suggested to allow nascent merozoites to rapidly activate their development cycle upon invasion of a new erythrocyte^62^. However, two proteomic analyses of *E*. *histolytica* have identified several membrane trafficking proteins (among others) present in the cyst^63,64^, suggesting that at least some MTS proteins are retained.

### Presence of divergent Entamoeba dynamin proteins during encystation

One final membrane-trafficking gene of interest was identified as a member of subcluster 5, a dynamin-related protein (Drp). DRPs have been shown to be involved in membrane trafficking steps such as vesicle scission and vesicle fusion, as well as other cellular processes requiring membrane deformation including cytokinesis. Given that the single dynamin encoded by the enteric parasite *G*. *intestinalis* is required in its encystation^31^, we asked whether Drps might also play a role in encystation in *Entamoeba*.

The *E*. *histolytica* genome encodes four dynamin homologues, EhDrp1 (EHI_013180), EhDrp2 (EHI_052740), EhDrp3 (EHI_174650), and EhDRP4 (EHI_139440). We further identified their homologues in the *E*. *invadens* genome: EiDrp1 (EIN_081190), EiDrp2 (EIN_114780), EiDrp2a (EIN_428080), EiDrp3 (EIN_376410) and EiDrp4 (EIN_080030), based on sequence similarity.

While Drp 1–4 are comparable in length and molecular weight to the other dynamin family members, Drp3 and Drp4 appear to differ in domain structure from other dynamins. While *Entamoeba* Drp1 and 2 contain the classic N-terminal large GTPase domain and C-terminal GTPase effector domain, only the N-terminal large domain was identified in Drp3 and Drp4 using Pfam^65^, SMART^66^, and NCBI’s Conserved Domain Database^67^. However, Drp3 and Drp4 are alignable in the region downstream of the GTPase domain, suggesting sequence divergence and perhaps non-identified functional domains.

### Phylogenetic analysis of DRPs in *Entamoeba spp*

**Figure 3:**
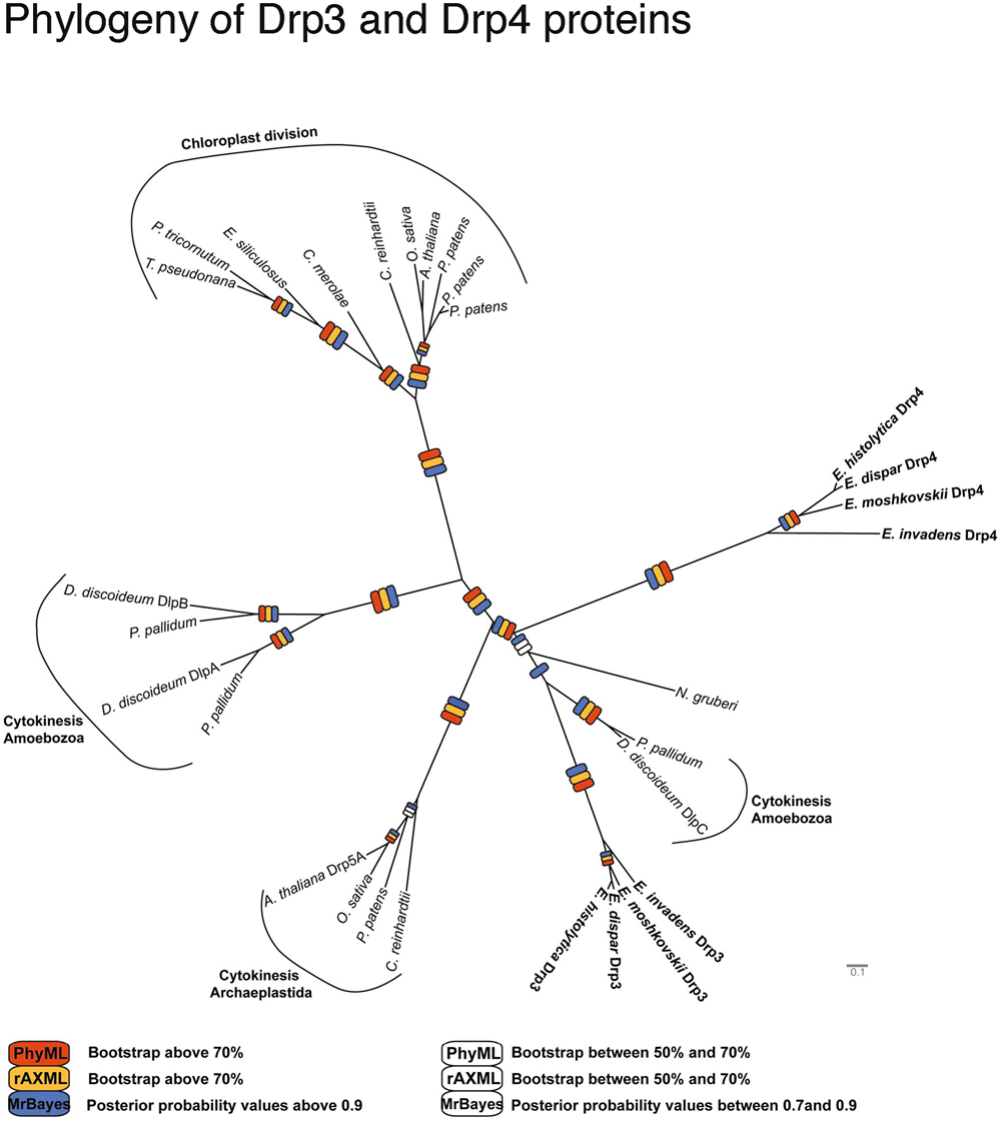
Phylogenetic analysis of the unusual dynamin-like proteins Drp3 and Drp4. This protein family contains dynamins involved in chloroplast division and cytokinesis and no homologs have been identified in humans. The PhyML Maximum Likelihood topology is shown. Bootstrap values and posterior probability values were calculated using PhyML, RAxML and MrBayes. The dataset contained 29 sequences with 572 informative residues (see Supplementary Table S7 for accession numbers).

To assess how the *Entamoeba* Drps are phylogenetically related to other dynamins, we performed extensive global and amoebozoan phylogenetic analyses. The initial dataset contained 133 dynamins and dynamin related sequences (Supplementary Figure S4, which was then separated into Drp1 and Drp2 sequences (Supplementary Figure S5), and Drp3 and Drp4 sequences, with an emphasis on amoebozoan taxa (Fig. 3). The phylogenetic reconstructions clearly show conservation of the orthologues among the *Entamoeba* species. *Entamoeba* Drp1 and Drp2 clustered separately from other functionally characterized members of the dynamin protein family (Supplementary Figure S4) but their function is difficult to discern. However, *Entamoeba* Drp3 and Drp4 formed a well-supported relationship with free-living non-parasitic Amoebozoa, and the archaeplastid and Stramenopile lineages involved in cell and chloroplast division (Supplementary Figure S4 and Fig. 3). *Entamoeba* Drp3 and Drp4, together with the amoebozoan *D*. *discoideum* DlpC and *Polysphondylium pallidum* Drp homologue (EFA76188) form a well-supported clade, with the *Entamoeba* Drp4 clade being the outgroup to the other sequences. Importantly, there is no such human dynamin orthologue, therefore these divergent *Entamoeba* DRPs may be prospective therapeutic targets if they are relevant to cyst formation.

Surprisingly, the basal red alga *Cyanidioschyzon merolae* groups with the phaeophycete taxa *Thalassiosira pseudonana*, *Phaeodactylum tricornutum* and *Ectocarpus siliculosus*, whose secondary plastid originates from a red algal endosymbiont (Fig. 3). This might suggest that these chloroplast-containing stramenopiles acquired this dynamin through the secondary plastid endosymbiosis event. This reconstruction is in agreement with an earlier analysis that suggested a link between cytokinesis and chloroplast division^68^.

### Divergent Drps are significantly expressed in late encystation stage in *E. invadens*

Identification of these unusual Drps prompted us to look more closely at their expression during encystation. To this end, exponentially growing cells were glucose starved to induce cyst formation. In order to see more specifically how these Drps are expressed, we performed reverse transcription PCR and quantitative PCR on mRNA sampled at the following time points: 0 hpi, 24 hpi, 28 hpi, 32 hpi, 36 hpi, 40 hpi, 44 hpi, and 72 hpi. Cyst formation was monitored by fluorescent microscopy using Calcofluor White, which stains the chitin cell wall (Supplementary Figure S6). In addition to monitoring cyst formation, total RNA was isolated at each time point and the expression of chitinases (known encystation markers) was analysed (Supplementary Figure S7). In agreement with previous studies^54^, chitinases are expressed during cyst formation, with the highest expression between 32 and 44 hpi.

**Figure 4:**
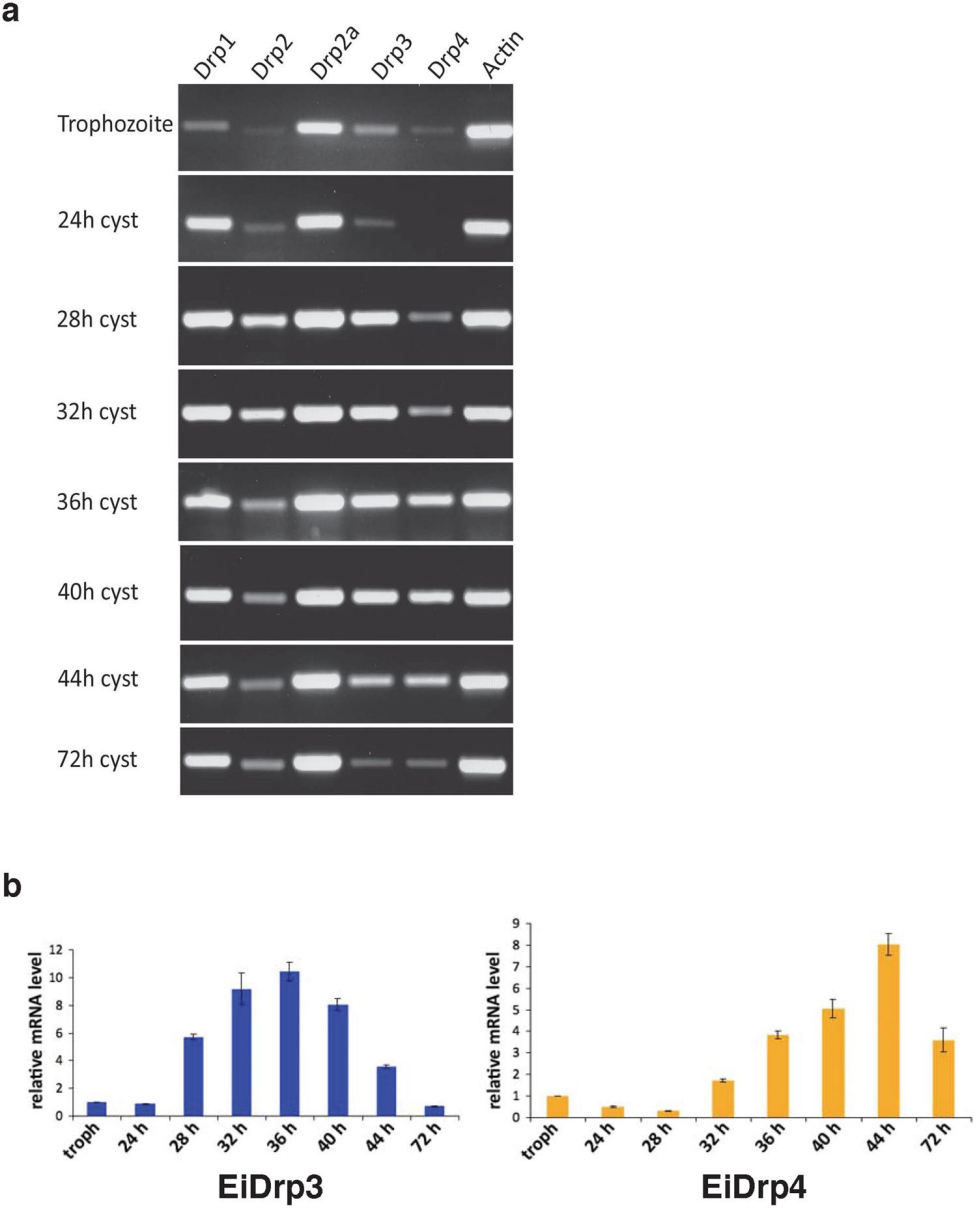
Semi-quantitative expression of all *E*. *invadens* Drps and quantitative expression of divergent EiDrp3 and EiDrp4. *E*. *invadens* dynamin expression was assessed during cyst formation using semi-quantitative RT-PCR (**a**). Comparative analysis indicates that the unusual Drp3 and Drp4 dynamins that are absent in humans are only expressed during cyst formation. Stages shown are trophozoites, early cysts (24–28 hpi), mid cysts (32–40 hpi) and late and mature cysts (44–72 hpi). Actin expression was used as a loading control. Quantitative real-time PCR analysis of unusual *Entamoeba* dynamins during cyst formation (**b**). Relative quantification of *E*. *invadens* Drp3 (left) and Drp4 (right) expression in trophozoites versus cysts is shown. Time point of RNA extraction is indicated on the x-axis (early cysts: 24–28 hpi, mid cysts: 32–40 hpi and late and mature cysts: 44–72 hpi) and relative mRNA levels is shown on the y-axis. Standard deviations are based on triplicate experiments.

During cyst formation, reverse transcription PCR revealed that all *E*. *invadens* Drps are up-regulated, however the degree of which is stage-specific for individual genes (Fig. 4a). EiDrp1 and EiDrp2 are expressed at low levels in the trophozoite stage, but EiDrp1 expression increases after 24 hpi and remains high until the late cyst stage, while EiDrp2 expression peaks at 28–32 hpi, and drops but remains constant after 36 hpi. Drp2a is constitutively highly expressed, suggesting a general cellular role for this gene.

RT-PCR, qPCR and Western blotting results show that the two divergent Drps are predominantly expressed during cyst formation (Fig. 4 and Supplementary Figure S8). To directly compare gene expression, we determined the PCR amplification efficiency for each primer pair, with values of 106% for EiDrp3, 104% for EiDrp4, and 109% for the reference gene SKI-interacting protein SKIP. EiDrp3 expression increases during early encystation (28 hpi), peaks at mid-encystation (36 hpi), and drops during late encystation (40 + hpi). EiDrp4 expression peaks in late encystation, at 44 hpi, and drops once again in the mature cyst (72 hpi). The RNA-Seq and subcluster analysis also indicates that the expression of both EiDrp3 and EiDrp4 increases during encystation, as they are members of subclusters 1 and 10, respectively. These genes are therefore likely to be involved in cyst formation, and represent potential therapeutic targets as they are highly divergent from human dynamins.

### Divergent Drps change cellular locations during encystation

**Figure 5:**
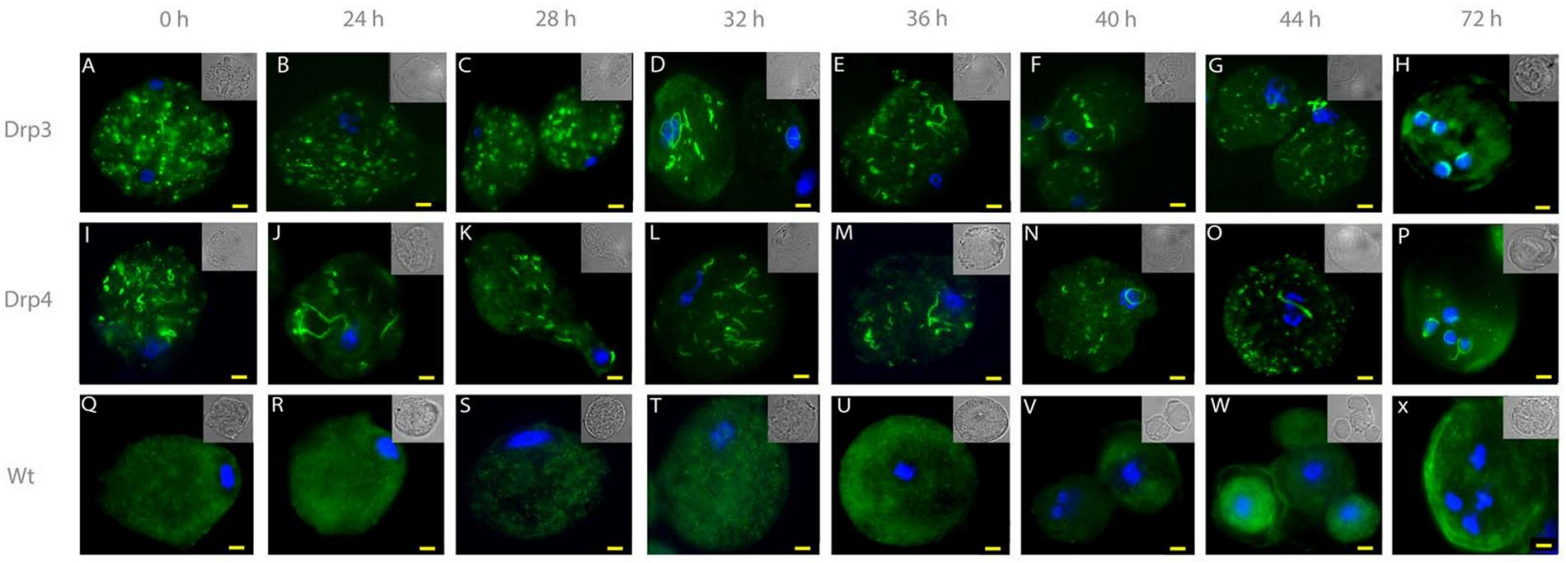
Localisation of divergent Drps during encystation of *Entamoeba invadens*. Hemagglutinin-tagged *E*. *invadens* Drp3 and Drp4 were stably overexpressed using p*Ei*NEO-LUC^69^ under the control of the ribosomal protein gene L3 promotor. The HA-tag was visualised using anti-HA antibodies (ThermoFisher) as primary antibodies and secondary antibodies conjugated with Alexa Fluor 488 (Abcam). Shown are representative images of the most frequently observed phenotype at each time point, based on data in Supplementary Figure S9. Trophozoites and encysting cells were collected at indicated time points. Wildtype *E*. *invadens* IP-1 were treated the same to indicate absence of cross-reactivity. Nuclei are stained using DAPI and a bright field image is shown in the top right of each image. Bar equals 3 µm.

We have clearly demonstrated that the highly divergent Drp3 and Drp4 are upregulated during encystation. If these proteins play some role in encystation, it is likely that their cellular localization would change during the course of the encystation process. To investigate the possibility, we produced recombinant *E*. *invadens* cell lines producing hemagglutinin-tagged versions of Drp3 and Drp4 expressed using the pEiNEO-LUC vector developed in the Bhattacharya laboratory^69^. This vector has been demonstrated to continuously express inserted genes under control of the promoter of ribosomal protein gene L3 during stage conversion from trophozoite to cyst and back. Due to the asynchronous nature of *E*. *invadens* encystation^70^, we observed several different localization patterns variably across the time points (Supplementary Figure S9); however, the pattern representing the majority of cells at each time point is shown in Fig. 5. The localization of both Drp3 and Drp4 in the trophozoite seems punctate and diffuse, and Drp3 is localized to fewer but larger structures than Drp4 (Fig. 5). For Drp3, the localization stays similar for the first 28 hours after which the number of punctate structures reduces and more elongated structures appear (Fig. 5 and Supplementary Figure S9). The final cyst localization seems close to the nucleus suggesting a possible association with the ER. Drp4 on the other hand seems more or less similar throughout encystation but also localizes around the nucleus in the final cyst stage (Fig. 5 and Supplementary Figure S9).

## Discussion

*E*. *invadens* is a well-established model system in which to study cyst formation of the related human pathogen *E*. *histolytica*. As cyst wall formation by secretion of various lectins and chitin is critical to pathogenicity in *Entamoeba*, we have focused on the role of the membrane trafficking system (MTS) in this process.

The MTS gene complement of *E*. *invadens* and *E*. *histolytica* is highly similar. As these species are not immediate sister taxa^71^, these data might be used as a proxy for studying other *Entamoeba* species. Vesicle formation machinery presence is well-conserved relative to that of the model systems of human and yeast. Only the ESCRT family of proteins is relatively incomplete, although we cannot exclude the possibility that the potentially missing genes are present but highly divergent, and refractory to identification by BLAST searches alone. However, even in their absence, it is likely that the ESCRT complex is capable of generating multivesicular bodies, especially given that many ESCRT components show patterns of increased expression in the mature cyst. MVBs are sorting organelles; usually their contents are destined for lysosomal degradation, but can also be secreted via an unconventional pathway involving autophagy machinery^72^.

Overall, vesicle fusion machinery is less well-conserved. None of the SNARE proteins involved in retrograde trafficking from the Golgi to the ER could be found (Syntaxin 18, Sec. 20, Use1, Sec. 22)^40^. Additionally, the COG complex, which is involved in intra-Golgi trafficking^43^, appears to be heavily reduced (only two subunits out of eight could be positively identified). Dsl1 appears to be missing, but it is typically partially or completely lost in organisms that lack peroxisomes^73^. In these cases where very few or no members of a complex could be identified, false negatives due to divergence are much less likely. *Entamoeba* does not have a ‘stacked’ Golgi body, instead, large Golgi ‘vesicles’ surround the nucleus, as visualized by confocal microscopy of amoebae stained with anti-Arf antibodies^74^. One may speculate that these potential losses of intra-Golgi and Golgi-ER trafficking machinery are correlated with *Entamoeba*’s alternative Golgi organelle structure. However, the COPI coat complex, which also supports retrograde Golgi-ER trafficking is nearly complete, and almost all subunits have undergone gene duplication events in both *Entamoeba* species. This suggests that the typical Golgi-to-ER trafficking pathway is functional and may even have two functionally distinct COPI coat complexes. Additionally, two paralogues of the SM protein that functions in this step are present in both *Entamoeba* species, lending further support for this trafficking pathway. These results suggest that Golgi-ER trafficking in *Entamoeba* differs from what is required in mammalian and yeast cells^75,76^, in that it is apparently accomplished without the SNAREs and MTCs that tether incoming vesicles to target membranes in opisthokont systems.

The MTC complexes of the endocytic system are similarly reduced. GARP, which supports endosome-TGN trafficking^49^, appears to be missing one subunit (Vps52), and while the core TRAPP and VpsC core subunits are largely conserved, the subunits that are specific to TRAPPII (another early endosome-TGN trafficking tether)^77^, and both Corvet and HOPS (early and late endosome/lysosome trafficking, respectively)^78^ could not be found. Our results corroborate those shown in Klinger *et al*. 2013, in which neither Dsl1 nor Corvet were identified^73^. However, other MTS proteins that support these functions are present, such as the retromer complex and adaptor protein complexes AP1 and AP3. Again, these pathways are likely functional, but perhaps do not require the canonical MTCs for vesicle fusion. In both this case and that of the Golgi-to-ER trafficking pathway above, it is possible that analogous machinery has evolved to take the place of the more canonical components. This would make for attractive therapeutic targets, but can only be addressed by molecular or biochemical means. Our identification of machinery (e.g. the VpsC or COPI complexes) that could serve as bait for interrogation to identify protein interactors should stimulate these types of analyses in the future.

Between *E*. *histolytica* and *E*. *invadens*, the presence and number of paralogues of MTS proteins is relatively similar, with the exception of Arf and Rab GTPases, and their regulators. While there has been significant innovation in both species, we do not suspect that this is related to encystation, as that is a feature of both organisms. Ras family GTPases commonly vary in number expanded in closely related taxa^79^, and in this case, may instead be an adaptation to their respective hosts, or other lifestyle factors. As shown in Supplementary Table S3, one-to-one orthology was observed in at least 50% of the Rab GTPases, and Arf and Rab regulators, suggesting a balance of functional retention and innovation. Given that the small GTPases are variably expanded, it is consistent that the number of cognate GAPs and GEFs similarly fluctuates. However, there are six *E*. *invadens*-specific Rab genes that are members of subclusters 1, 2, 5, and 9 – which are up-regulated during encystation – suggesting they may be involved in this process. Therefore, we cannot overlook the possibility that there may be some differences in the trafficking events underlying encystation in the two *Entamoeba* species. There are also a number of *E*. *histolytica*-specific Rabs, and again, these may be relevant to encystation as it occurs in their respective hosts.

The two pathways that chiefly characterize encystation are the secretory pathway, and TGN-endosome recycling. The purpose of the former is clear: to support Jacob and Jessie lectin secretion and therefore cyst formation. This correlates with the increase in expression of chitin-cleaving chitinases during encystation (Supplementary Figure S7)^54^. The role of the TGN-endosome recycling pathway in encystation is less clear. During encystation, vesicles containing cyst-forming material are transported to the cell surface, and the extra membrane from this exocytic process must be retrieved. It may be that endosome-to-TGN trafficking functions to retrieve surface proteins that have been internalized with this membrane, thus preventing them from being trafficked to the lysosome for degradation.

It is interesting to consider that many of the genes that are differentially expressed during encystation are small monomeric G proteins and their regulators, i.e. Arf and Rabs. This suggests that these proteins that control vesicle formation and fusion kinetics may be the ‘gatekeepers’ of cyst formation. Our work and others’ has shown that Rabs are particularly differentially expressed during cyst formation^22^. Unfortunately, little work has been done to investigate the function of Rabs in *Entamoeba*, other than that of Rabs 5^20^, 7^21^, 8^80^, 11^81–83^, RabA^57^, and RabB^60^. Therefore, these unstudied Rabs represent potential therapeutic targets, in particular, those proteins that are highly expressed during encystation.

It has been previously shown that *E*. *histolytica* Rab11A is involved in encystation^81^, and Rab11 paralogues are also involved in protein recycling, as are their human orthologues^81–83^. In *E*. *invadens*, Rab11A is highly but consistently expressed at time points during encystation, while Rab11B is a member of subcluster 6, and appears to decrease slightly in expression during encystation. By contrast, Rab11C and Rab11D expression increases in early encystation, peaks at late encystation, and drops again in the mature cyst (subcluster 2), implicating Rab11C and Rab11D in cyst formation. Much of the work on *Entamoeba* Rabs has examined their involvement in phagocytosis and pathogenesis. Rab5 and Rab7, particularly, have been shown to be involved in phagosome and pre-phagosomal vacuole maintenance^20,21,84,85^. Three Rab7 paralogues are members of subclusters 1 and 2, suggesting that they may be involved in encystation, in contrast to other paralogues that have different expression patterns. This again lends support to the idea of a highly specialized MTS in *Entamoeba* where gene families encode functionally divergent paralogues.

The only clear pattern of downregulation during cyst formation is seen in some endocytic and phagocytic trafficking components, which is congruent with the idea that the amoeba is preparing to become a quiescent cyst. We propose that part of this preparation involves generating mRNAs for various endocytic and degradative trafficking genes that may be kept untranslated for rapid translation during excystation, similar to the mRNA localization that occurs in *Drosophila* embryogenesis^86^. Proteomics analyses of *E*. *histolytica* cysts have shown a handful of MTS proteins, among many others, present in mature cysts^63,64^, raising the possibility that these mRNAs may indeed be translated. Two proteins identified were clathrin heavy chain and a TBC-B homologue (EHI_094140). Our analysis shows that the *E*. *invadens* orthologues of both genes are members of subcluster 10, whose expression peaks in the mature cyst. However, at this time, we cannot differentiate between long-lived proteins generated during encystation and untranslated mRNA pools kept in the cyst for excystation. Regardless, this raises the question of a mechanism to prevent mRNA translation, degradation, or protein functioning in the quiescent cyst. Cysts can remain infective for weeks under optimal conditions^87^, suggesting that there is a mechanism by which mRNA (or protein) is stored in an untranscribed or inactive manner during this time. Interestingly, there is evidence for the accumulation of mRNA in cysts of the amoeba *Acanthamoeba* ^88^, and the ciliate *Colpoda inflata* ^89^, suggesting that mRNA storage in cysts occurs in other organisms, although the mechanisms have not yet been deduced.

A similar study to ours was performed by Ehrenkaufer *et al*.^90^, which assessed genome-wide transcriptional changes in *E*. *invadens* using RNA-Seq. They generated gene expression profiles, and identified Gene Ontology (GO) terms enriched in each profile or profile group. Genes annotated with the GO term ‘vesicle-mediated transport’ were found to be significantly enriched in profiles with a pattern of increased gene expression during encystation. We found that overall, the membrane trafficking gene expression patterns we assigned are largely consistent with the profile assignments by Ehrenkaufer *et al*.^90^. Furthermore, consistent with our findings, they identified genes involved in RNA metabolism expressed in the mature cyst, potentially preconfiguring the cell for rapid excystation.

We identified four dynamin related proteins in *E*. *histolytica* and five in *E*. *invadens*. *Entamoeba* Drp1 and Drp2(a) both resemble canonical dynamin related proteins, while Drp3 and Drp4 do not. Immunofluorescence studies have previously shown EhDrp1 to bind to the nuclear membrane^91^, while the function of Drp2/Drp2a has not been studied. Although the phylogeny is not internally well-resolved, these proteins do group with other canonical dynamins, including metazoan dynamins. Drp3 and Drp4, however, form a well-supported clade with other amoebozoan, *Naegleria*, plant, and stramenopile and alveolate sequences, to the exclusion of any metazoan sequence. These homologues have been reported to work in cytokinesis in *Dictyostelium* and *Arabidopsis* and suspected to work in chloroplast division in both plant and Stramenopiles, based on phylogenetic affinity^68^. However, the *Entamoeba* Drp3 and Dpr4 proteins are solely expressed during encystation, during which time two rounds of nuclear replication occur, but cytokinesis does not^92,93^. It is likely that that the original function of this Drp subfamily is cytokinesis, based on this function being retained in two evolutionarily distant organisms, suggesting *Entamoeba* Drp3 and Drp4 have been repurposed in encystation, although their function is not known. It is worth noting that the RNASeq expression patterns were confirmed by RT-PCR, qPCR and Western blotting, serving not only to give confidence about the dynamic expression, but also as a validation of the RNASeq data generally.

The cellular location of these divergent Drps changed dramatically during encystation from a punctate diffuse localization to one surrounding the nucleus in the mature cyst suggesting an association with the ER. Further co-localization and knock-down experiments will allow us to understand the role of these unusual Drps more fully. The fact that these Drps do not have human orthologues raises the possibility of their use as therapeutic targets. In addition to these, we identified a SNARE protein, Syp7, which is specifically up-regulated during encystation, and lacks a human orthologue^94^. While disrupting them would not treat an infection, it could reduce parasite spread in a population. Currently, lumenal amoebicides, which target organisms in the intestinal lumen rather than invasive tissue infection, include diloxanide furoate, iodoquinol, and paromomycin. Diloxanide furoate has an efficacy >50%, while the other two have an efficacy rate >80%, and are relatively safe^95,96^. However, some asymptomatic cases still fail to respond to treatment with these drugs, and it has been previously suggested that high rates of genetic polymorphisms in a limited geographical region, and extensive geographic diversity in general, may be responsible^6,97^. Additionally, drug resistance is an ever-growing problem, and *E*. *histolytica* resistance to treatment with metronidazole, which is effective against invasive *Entamoeba*, has been reported^98^. The development of new amoebicidal drugs is therefore still a valuable pursuit, especially drugs that limit cyst production, which is key in areas with poor sanitation.

In conclusion, we have shown, using the model *E*. *invadens*, that the membrane trafficking system is modulated during the encystation process. The normal secretory pathway supports exocytosis of cyst wall factors, and TGN-endosome recycling may be a mechanism to control membrane homeostasis. Furthermore, early endocytic factors such as clathrin, as well as phagocytic genes, appear to be upregulated in the mature cyst, suggesting that mRNAs or perhaps protein is stored in the cyst in preparation for excystation. Finally, *Entamoeba* expresses two highly divergent dynamin-related proteins specifically during encystation. Orthologues in other taxa are involved in cytokinesis, suggesting that the *Entamoeba* proteins have undergone neofunctionalization. These represent potential drug targets to be explored in future, as an additional way to lower diarrheal disease burden around the world.

## Methods

### Strains, Culture Conditions and Cyst Formation

Trophozoites of *E*. *invadens* IP-1 were cultured in 50 mL culture flasks with unvented lids in axenic LYI-S-2 medium at 20 °C^99^. To induce encystation, trophozoites in logarithmic growth phase were transferred into axenic glucose-free 50% LY-G medium^100^. For RNA-Seq experiments, samples were taken at 0, 24, 48, and 72 hours post-induction (hpi) of cyst formation; to examine the expression of dynamin-related proteins in cyst formation, samples were taken 0, 24, 28, 32, 36, 40, 44 and 72 hpi in accordance with previous studies^13,54,70,101^. The 72 hpi mature cyst sample was treated with 0.05% Sarkosyl (Sigma Aldrich) to destroy any remaining trophozoites. Cells were pelleted and washed in phosphate-buffered saline for subsequent RNA and protein extraction. *Escherichia coli* strain DH5α was maintained in LB containing 100 µg /ml ampicillin when used to support vector maintenance.

### Amplification and Cloning of EiDrp3 and EiDrp4

Genes encoding EiDrp3 (GenBank accession number EIN_376410) and EiDrp4 (EIN_080030) were PCR amplified from genomic DNA of *E*. *invadens* IP-1 using primers adding hemagglutinin (HA) tags at the 3′ end, for EiDrp3: 5′-ATG TCT ATC TTG AAT GAA CCA G-3′ and 5′-CTA TGC ATA GTC TGG AAC GTC ATA TGG ATA CAT TTC TCG CTC TTT GG-3′; and for EiDrp4: 5′-ATG ACA TCA ACA CAG ACA ATG CG-3′ and 5′-CTA TGC ATA GTC TGG AAC GTC ATA TGG ATA AAA GTT CAA TTT CTT TTT AAG TTG AGG-3′. The cycling parameters of PCR were: denaturation at 95 °C for 45 s; annealing at 50–56 °C for 45 s; elongation at 72 °C for 1 min/kb; 35 cycles, using GoTaq G2 Hot Start Green Master Mix (Promega). PCR products were purified with QIAquick PCR purification kit (Qiagen) and cloned into pGEM-T-Easy and confirmed by sequencing. The genes were subsequently cloned into the *E*. *invadens* expression vector p*Ei*NEO-LUC^102^, a very kind gift of Professor Sudha Bhattacharya′s laboratory (Jawaharlal Nehru University, New Delhi, India). To facilitate cloning, *Kpn*I and *Bam*HI sites were added via PCR: for EiDrp3: 5′-AGA AGA GGT ACC ATG TCT ATC TTG AAT GAA CCA G-3′ and 5′-TCT TCT GGA TCC CTA CAT TTC TCG CTC TTT GG-3′; for EiDrp4: 5′-AGA AGA GGT ACC ATG ACA TCA ACA CAG ACA ATG CG-3′ and 5′-TCT TCT GGA TCC CTA AAA GTT CAA TTT CTT TTT AAG TTG AGG-3′. Constructs were confirmed by sequencing.

### Transfection of *E. invadens*

Transfection was performed by electroporation according to Singh *et al*. 2012^102^. Briefly, *E*. *invadens* cells were grown up to 70% confluency, placed on ice for 15 min and harvested by centrifugation at 400 g for 5 min at 4 °C. Cells were washed once with PBS pH 7.0 and once washed with incomplete cytomix buffer (120 mM KCI, 0.15 mM CaCl_2_, 10 mM K_2_HPO_4_/KH_2_PO_4_ pH 7.5, 25 mM HEPES, 2 mM EGTA, 5 mM MgCl_2_, final pH 7.8). Subsequently, 2 × 10^6^ trophozoites were re-suspended in 600 μl of complete cytomix buffer (5 mM reduced glutathione and 2 mM ATP). Cells were transferred to a 0.4 mm electroporation cuvette. 100 μg of plasmid DNA was added. Two consecutive pulses of 3000 V/cm were applied at capacitance of 25 μF using a Bio‐Rad Gene Pulser Xcell Electroporation System. Electroporated cells were incubated on ice for 10 min, transferred to borosilicate tubes containing 12 ml of LYI-S medium supplemented with 15% heat inactivated adult bovine serum and 2% vitamin mix. 10 μg of G418 was added after 24 hrs. Cells were subcultured with G418 containing media every 72 hrs.

### Fluorescence microscopy

Immunofluorescence was performed according to Siegesmund *et al*.^103^. Trophozoites and cysts were harvested at 400 g/5 min, attached to poly-L-lysine coated glass slides for 20 min, fixed in 4% paraformaldehyde (PFA) for 45 min, and rehydrated with 1x PBS for 30 min. Trophozoites were permeabilized with 0.2% Triton X-100 in 1x PBS at room temperature for 1 hour. Cysts were permeabilized overnight at 4 °C. Cells were blocked with 2% BSA at room temperature for 2 hours and incubated with anti-HA primary antibodies (ThermoFisher) diluted to 1:500 in 0.2% Triton X-100, 0.02% sodium azide and 2% BSA for 1 hour at room temperature. Slides were washed twice with 0.2% Triton X-100 in 1x PBS for 3–5 min. and incubated with secondary antibodies conjugated with Alexa Fluor 488 (Abcam) diluted to 1:500 in 0.2% Triton X-100, 0.02% sodium azide, 1% BSA in 1x PBS, for 1 hour at room temperature. Slides were washed with 0.2% Triton X-100, 0.5% BSA in 1x PBS, 3 times for 5 min. each. Slides were mounted with Fluoroshield Mounting Medium with DAPI (Abcam) and covered immediately. Cells were examined using an Olympus IX-81 inverted microscope equipped with the appropriate filter combinations and a 100x objective. Cyst formation was monitored by Calcofluor White staining; two drops of 0.05% Calcofluor White solution (Fluka) were added to a microscope slide with 40 μL encysting cell suspension and incubated for 5 minutes at room temperature before applying the coverslip. Cysts were observed under ultraviolet light by fluorescent microscopy. Spores from the ascomycete fungus *Magnaporthe grisea* were used as a positive control. For a negative control, *E*. *invadens* mature cysts were not treated with Calcofluor White (Supplementary Figure S10).

### RNA extraction for RT-PCR and qPCR

Trophozoites and the various cyst stages were harvested and washed in PBS. RNA was isolated using Tripure (Roche). Total RNA isolated was treated with DNAseI (Ambion) for 1 hour at 37 °C to remove any genomic DNA. To test for successful DNA removal, RNA samples were subjected to PCR using gene specific primers. Purified RNA was quantified spectrophotometrically using a Nanodrop ND-1000 (Thermo-Fisher). First strand cDNA synthesis was started from 2 μg RNA using Promega ML-MLRT reverse transcriptase in combination with random primers.

### Transcription analysis during cyst formation

PCR was performed for 30 cycles at 95 °C for 45 s, 53 °C for 45 s, and 72 °C for 45 s using the GoTaq Master Mix (Promega). Prior to PCR, the annealing temperature for the primer pairs was determined experimentally by gradient PCR using a mix of cDNA samples from each time point. The chitinase primers and corresponding annealing temperatures were taken from a previous study^54^. All other primers used in this study are listed in Supplementary Table S4. Quantitative real-time PCR experiments were carried out using a Stratagene Mx3005 P real-time PCR system, Brilliant II Sybr Green qPCR Master Mix (Agilent) and cDNA prepared as described above. PCR was performed for 40 cycles at 95 °C for 30 s, 58 °C for 30 s, and 72 °C for 30 s. Primer specificities and PCR amplification efficiencies were analysed by melt curve and standard curve experiments. The melt curve was established for each primer pair by increasing temperatures at the end of the PCR reaction from 55 to 94 °C, and monitoring fluorescence. The standard curve for each primer pair was constructed by using serial diluted cDNA templates for PCR. qPCR experiments were performed in technical triplicates. The reference gene of choice was the SKI-interacting protein SKIP (Accession number: AANW01003268), which has been shown to express stably during cyst formation in *E*. *invadens* ^104^.

### RNA-seq analysis

cDNA libraries were constructed for each time point (0, 24, 48, 72 hpi) using the Illumina TruSeq RNA Library Preparation kit and run on an Illumina HiSeq. 2000 sequencer, generating 100 bp paired-end sequences. Raw reads were trimmed, clipped and filtered using the FASTX-Toolkit (http://hannonlab.cshl.edu/fastx_toolkit/). The first 12 bp were trimmed of the reads using the fastx_trimmer, and adapter sequences were removed using the fastx_clipper tool (using ‘AGATCGGAAGAGC’ as adapter string). The 3′ end of the reads were quality-trimmed using a Perl script written by Joe Fass (Bioinformatics Core Facility at the UC Davis Genome Center, USA) (Trim.slidingWindow.pl; see Supplementary File 1), that uses a sliding window to trim the first base with a quality Phred score <20, in the first sliding window with a quality Phred score <20. Reads less than 30 bp were removed and processed sequences were paired using a Python script (https://github.com/lexnederbragt/denovo-assembly-tutorial/blob/master/scripts/pair_up_reads.py). Transcripts were assembled *de novo* using CLC Genomics Workbench software package (CLC, Denmark) clustered using CD-HIT-EST^105^ and subsequently used to correct and refine the *E*. *invadens* IP-1 genomic sequence using a hybrid genomic guided and *de novo* approach to identify potential new transcripts (PASA)^106^. All raw sequence data are accessible at the NCBI Sequence Read Archive through accession numbers MF361146 - MF361261.

The assembled transcripts were preliminary annotated against the *E*. *invadens* IP-1 (JCVI) and *E*. *histolytica* HM-1:IMSS (JCVI) annotated transcripts and proteomes (publically available at amoebaDB.org). All-against-all nucleotide BLASTN and BLASTX (translating a nucleotide query in six reading frames) searches were conducted^107^. Annotations were assigned to the RNA-Seq transcripts when hits had an E- value of at least 1e-3 and coverage of at least 50%. When no adequate hit was available the transcript was annotated as hypothetical. A second genome sequence of *E*. *invadens* IP-1 independently constructed by The Sanger Institute (NCBI GenBank, accession number AANW00000000) was also used to annotate the RNA-Seq transcripts. Independent BLASTN was conducted for each RNA-Seq transcript against the NCBI Nucleotide BLAST database using BLAST + ^108^. Annotations were accepted for BLASTN hits with an E-value < 1e-2 with a minimum coverage of 50%.

### Gene expression analysis

The Trinity package^109,110^ and edgeR^53^ were used to determine the levels of gene expression using the mapped transcripts. Within the Trinity package, RSEM was used to calculate a Maximum Likelihood estimation of abundance based on the number of reads mapped at both an isoform level and gene level, given as fragments per kilobase of exon per million fragments mapped (FPKM)^110^. Differentially expressed genes were identified using edgeR, with a dispersion value of 0.4 (recommended for datasets with no biological replicates)^53^. Transcripts with a false discovery rate <0.01 and a minimum log_2_ fold change of 2-fold were classified as significantly differently expressed between two time points. TMM-normalized FPKM values for each gene across the four time points (0 hpi, 24 hpi, 48 hpi and 72 hpi) were calculated with default parameters^111^ producing expression profiles for each gene. As the Expectation Maximization algorithm of RSEM assigns reads to isoforms based on the most likely relative abundances of the transcripts, in some cases alternative transcripts were identified as very lowly expressed at only one time point, and these appeared to not be biologically significant. Therefore, only transcripts with the highest average expression across conditions were considered in our analyses.

Significantly differentially expressed genes (FDR < 0.01, fold change >2) were clustered in order to identify groups of similarly regulated genes using *k*-means clustering (Supplementary Table S5). The optimum number of clusters was determined using the median number of clusters proposed by 18 independent indexes (R package NbClust)^112^. Hierarchical clustering of differentially expressed transcripts with similar expression patterns using Euclidean distance was conducted using scripts from the Trinity package (clustering using ‘analyze_diff_expr.pl’ with the parameters –P 1e-2 and –C 1 and clusters split using ‘define_clusters_by_cutting_tree.pl’, parameters –K 10). To determine which variables (time points) within the data set were contributing the largest proportion of variation between the clusters, defined using the above techniques, Principal Component Analysis (PCA) was carried out. Scaled FPKM values for each gene at each of the four time points were used for the PCA analysis and a biplot was created using custom R script (Supplementary File 2) (R Core Team, 2013).

### Comparative genomics

For all membrane trafficking components, *Homo sapiens* sequences that have been functionally characterized were used as queries to search the individual transcript files for each of the four time points, as well as the combined set of *E*. *invadens* transcripts. Additionally, searches were repeated in the predicted proteome of the *E*. *invadens* genome at AmoebaDB (http://amoebadb.org/amoeba/). In cases where an MTS component has not been described in human, the sequence from the organism in which it is best described or evolutionarily is closest to *E*. *invadens* was chosen; these include *Arabidopsis thaliana*, *Naegleria gruberi*, *Saccharomyces cerevisiae*, and *Trypanosoma brucei*. tBLASTn searches were performed with a BLOSUM62 matrix and an E-value cut-off of 0.05 to identify potential homologues, which were then confirmed by reverse BLASTx searches into the *H*. *sapiens* genome. To be considered homologous, *E*. *invadens* sequences must retrieve the original human query sequence or a clear orthologue with an E-value < 0.05. As *E*. *invadens* sequences tend to be divergent, those that retrieved a clear orthologue of the human query by searching the NCBI non-redundant database with an acceptable E-value were also considered orthologous. Rab proteins were not searched for using BLAST, as a comparative genomic analysis of Rabs in *E*. *invadens* and *E*. *histolytica* was previously done by Nakada-Tsukui *et al*. 2010^23^.

Dynamins were identified by searching the *E*. *histolytica* genome using previously classified dynamin sequences from across the eukaryotic tree of life. The four *E*. *histolytica* dynamins, EhDrp1 (EHI_013180), EhDrp2 (EHI_052740), EhDrp3 (EHI_174650), and EhDRP4 (EHI_139440), were then used to search all available *Entamoeba* genomes on Sanger (http://www.sanger.ac.uk/) and AmoebaDB (http://amoebadb.org/amoeba/).

### Phylogenetics

Several MTS gene families are highly paralogous, and phylogenetic trees were generated to confirm the identity of homologues found via BLAST. Sequences were aligned using MUSCLE v3.8.31^113^, visualized using Mesquite v.3.03^114^, and then manually masked and trimmed to include only homologous positions. Masked alignments are available upon request. A list of sequence accessions, corresponding gene identifiers, and tree meta-data are listed in Supplementary Table S6. ProtTest v.3.4^115^ was used to determine the best-fit model of sequence evolution, which was LG^116^ with gamma-distributed rate variation across sites (+G) and observed amino acid frequencies (+F) in the following trees: adaptin GADEZ subfamily, ESCRT Snf7 family, the TBC/RabGAP family, and the DENN RabGEF family. Both the Rab and Arf trees to assess *Entamoeba* spp. sequence orthology also used this model. The LG + G model was used in ArfGEF and SNARE Qc subfamily trees, and the WAG + G + F model^117^ was used in ArfGAP trees. Phylobayes v3.3^118–120^ and MrBAYES v3.2.2^121,122^ were used for Bayesian inference of phylogeny, and RAxML v8.1.3^123^ was used for Maximum-Likelihood analyses. Phylobayes was run until the largest discrepancy observed across all bipartitions was less than 0.1 and at least 100 sampling points were achieved, MrBAYES was used to search treespace for a minimum of one million MCMC generations, sampling every 1000 generations, until the average standard deviation of the split frequencies of two independent runs (with two chains each) was less than 0.01. Consensus trees were generated using a burn-in value of 25%, well above the likelihood plateau in each case. RAxML was run with 100 pseudoreplicates. MTS gene phylogenies can be found in Supplementary Figures S1 and S2.

The dynamin sequences from *E*. *invadens*, *Entamoeba dispar* and *Entamoeba moshkovskii* were extracted from the respective genome projects and aligned with homologous protein sequences found in Archaeplastida, stramenopiles and *N*. *gruberi* using ClustalW in Seaview 4.2.12^124^. Manual masking and trimming was performed in Seaview. Three datasets were constructed including a global dynamin-family phylogeny, a separate Drp1/2 phylogeny and a Drp3/4 phylogeny. The large dataset contained 133 sequences with 229 informative sites. The Drp1 and Drp2 dataset contained 66 sequences with 230 informative sites and the Drp3 and Drp4 dataset contained 29 sequences with 572 informative residues. A complete list of taxa and accession numbers can be found in Supplementary Table S7. RAxML^123^ and the Maximum Likelihood program PhyML v3.0^125^ were used to generate trees for all datasets. MrBAYES^121,122^ was run for the Drp3/Drp4 dataset. Modelgenerator v.0.85^126^ was used to determine the best-fit model of sequence evolution, which was the LG model^116^ for all dynamin trees. For individual phylogenies, the following additional parameters were used: global dynamin phylogeny, LG + G; the Drp1/Drp2 phylogeny, LG + I + G (estimated proportion of invariant sites, + I); and Drp3/Drp4, LG + G. Bootstrap resampling was performed on ML trees with 100 replications for all three analyses. In addition, the Bayesian analysis for the Drp3/Drp4 dataset was performed using a mixed model accommodating 4 rate + inv categories containing 4 chains each. One million generations were calculated and trees sampled every 1,000 generations. The model stabilized rapidly and 200 trees were discarded as burn-in.

## Electronic supplementary material

Supplementary figures

Table S1

Table S2

Table S3

Table S4

Table S5

Table S6

Table S7

Python script

R script

Tree file

Tree file
