# Supplementary material for: Membrane Trafficking Modulation during Entamoeba Encystation
Source: Sci Rep. 2017 Oct 9;7:12854. doi: 10.1038/s41598-017-12875-6 (PMC5634486; doi:10.1038/s41598-017-12875-6)
Supplement: Supplementary file 1 — Supplementary figures [file 41598_2017_12875_MOESM1_ESM.pdf]

Supplementary figures

Membrane Trafficking Modulation during *Entamoeba* Encystation

Emily Herman[1], Maria A. Siegesmund[2]*, Michael J. Bottery[3*], Ronny van Aerle[2,4], Maulood Mohammed Shather[2], Elisabet Caler[5,#,**], Joel B. Dacks[1$], and Mark van der Giezen[2$]

[1]Department of Cell Biology, Faculty of Medicine and Dentistry, University of Alberta, T6G 2H7 Edmonton, Alberta, Canada.

[2]Biosciences, University of Exeter, Stocker Road, Exeter EX4 4QD, UK.

[3]Department of Biology, University of York, Heslington, York YO10 5DD, UK.

[4]Centre for Environment, Fisheries, and Aquaculture Science (Cefas), Barrack Road, The Nothe, Weymouth, Dorset, DT4 8UB, UK.

[5]J. Craig Venter Institute, 9714 Medical Center Drive, Rockville, MD 20850, USA.

[#]Current address: National Heart, Lung, and Blood Institute (NHLBI), National Institutes of Health (NIH), 6701, Rockledge Drive, Room 9144, Bethesda, MD, 20892-7950, USA.

[**]The views expressed in this manuscript are those of the authors and do not necessarily represent the views of the National Heart, Lung, and Blood Institute; the National Institutes of Health; or the U.S. Department of Health and Human Services.

[*]Both authors contributed equally.

[$]Corresponding authors.

Supplementary Figure S1. (A-T) Phylogenies of membrane trafficking system proteins used to classify paralogous gene families. For Phylobayes and MrBAYES trees, node values are posterior probabilities, and for RAxML trees, node values are bootstrap values. Note that DENN trees (R-T) were used to determine sequence orthology, but not classify DENN sequences. Trees are arbitrarily rooted. For a complete list of taxon identifiers, see Supplementary Table S6.

Supplementary Figure S2. Phylogenies of ArfGEF proteins used to determine orthology within the *Entamoeba* spp. For Phylobayes and MrBAYES trees, node values are posterior probabilities, and for RAxML trees, node values are bootstrap values. Trees are arbitrarily rooted. For a complete list of taxon identifiers, see Supplementary Table S6. The *Entamoeba*-specific Rab phylogenies are found as Nexus format tree files in Supplementary Files 3 and 4.

Supplementary Figure S3. Bioplot of gene expression cluster principal components. The arrows represent the loadings of the variables and their effect upon the first (58.4% variance) and second (33% variance) components, by which the subclusters can be almost exactly split. The length of the arrows represents the variances of each variable. Subclusters are colour coded for clarity.

Supplementary Figure S4. Phylogenetic analysis of the dynamin family. The phylogenetic reconstruction shown is a Maximum Likelihood tree constructed using PhyML and based on an alignment of 133 amino acid sequences containing 229 informative sites. The analysis includes dynamin protein sequences from all eukaryotic supergroups including Archaeplastida (green), Opisthokonta (brown), stramenopiles, alveolates, and haptophytes (light blue), Excavata (red) and Amoebozoa (purple). Superscript numbers indicate the number of sequences within a collapsed clade. Putative functions are mentioned next to the sequences. Nodes containing *Entamoeba* sequences are boxed. For visual purposes, the branch of the Opisthokonta Fzo1 clade was shortened by 70 %. For a detailed list of taxa and sequences, please see Supplementary Table S7.

Supplementary Figure S5. Phylogenetic analysis of classical dynamin proteins. Shown is a Maximum Likelihood topology constructed using PhyML based on 66 amino acid sequences containing 230 informative sites. Bootstrap values were calculated using PhyML and RAxML. Node colours are as shown in Supplementary Figure S4. Putative functions are

mentioned next to the sequences. Superscript numbers indicate the number of sequences within a collapsed clade. For a detailed list of taxa and sequences, please see Supplementary Table S7.

Supplementary Figure S6. Cyst formation in *Entamoeba invadens*. Cells were followed during encystation using bright-field microscopy (panels A-C) and cyst walls were stained with the chitin dye Calcofluor White (panels D-F). Cyst formation was divided into three stages similar to previous studies:[13,94–96] early cysts (24-28 hpi), mid cysts (32-40 hpi) and late and mature cysts (44-72 hpi). A and D, 24 hpi; B and E, 36 hpi and C and F, 72 hpi. Scale bar, 10 µm.

Supplementary Figure S7. Semi-quantitative RT-PCR analysis of E. invadens chitinase expression during encystation. Panels show the amplified product using chitinase-specific PCR primers from different time points during cyst formation. From left to right: chitinase 1 (accession number: AAB52724), chitinase 2 (ABC59330), chitinase 3 (ABC59331) and chitinase 4 (AB576188). Shown are trophozoites (A), 24 hpi (B), 28 hpi (C), 32 hpi (D), 36 hpi (E), 40 hpi (F), 44 hpi (G), and 72 hpi (H).

Supplementary Figure S8. Coomassie stained gel and Western blot analysis of total *E. invadens* protein using *Dictyostelium discoideum* anti-DlpA antibody. (A) Coomassie stained gel of *E. invadens* extracts from trophozoites, and cysts at 24 hpi, 36 hpi, 48hpi and 72 hpi, with a molecular weight marker on the left. (B) Western blot of anti-DlpA staining of extracts from *E. invadens* trophozoites, 24 hpi, 36 hpi, 48 hpi and 72 hpi. The *D. discoideum* antibody (kind gift of Dr. Miyagishima, RIKEN, Saitama, Japan) shows a positive band at ~90 kDa, coinciding with the predicted molecular weights of *E. invadens* Drp3 (~95 kDa) and Drp4 (~89 kDa), at 36-48 hpi of encystation.

Supplementary Figure S9. Subcellular localization of EiDrp3 and EiDrp4. HA-tagged EiDrp3 (top) and EiDrp4 (bottom) were constitutively expressed in *E. invadens* and localized by fluorescence microscopy. ~100 cells were observed for each time point and binned into observed phenotypes. Each micrograph is a representative fluorescence pattern, below which the number and percentage of cells observed with that pattern are listed for each timepoint (0 hpi, 24 hpi, 28 hpi, 32 hpi, 36 hpi, 40 hpi, 44 hpi, and 72 hpi). Values are shaded green to

reflect the number of cells observed with that fluorescence pattern, with darker shading indicating a higher number of cells.

Supplementary Figure S10. Controls for Calcofluor White staining. Positive and negative controls as seen using bright-field microscopy (panels A and B) and observed under UV light by fluorescence microscopy (panels C and D). Negative control (A and C), mature cysts (72 hour post-induction) that were not stained with Calcofluor White. Positive control (B and D), spores of the ascomycete fungus *Magnaporthe grisea*. Scale bar, 10 μm.

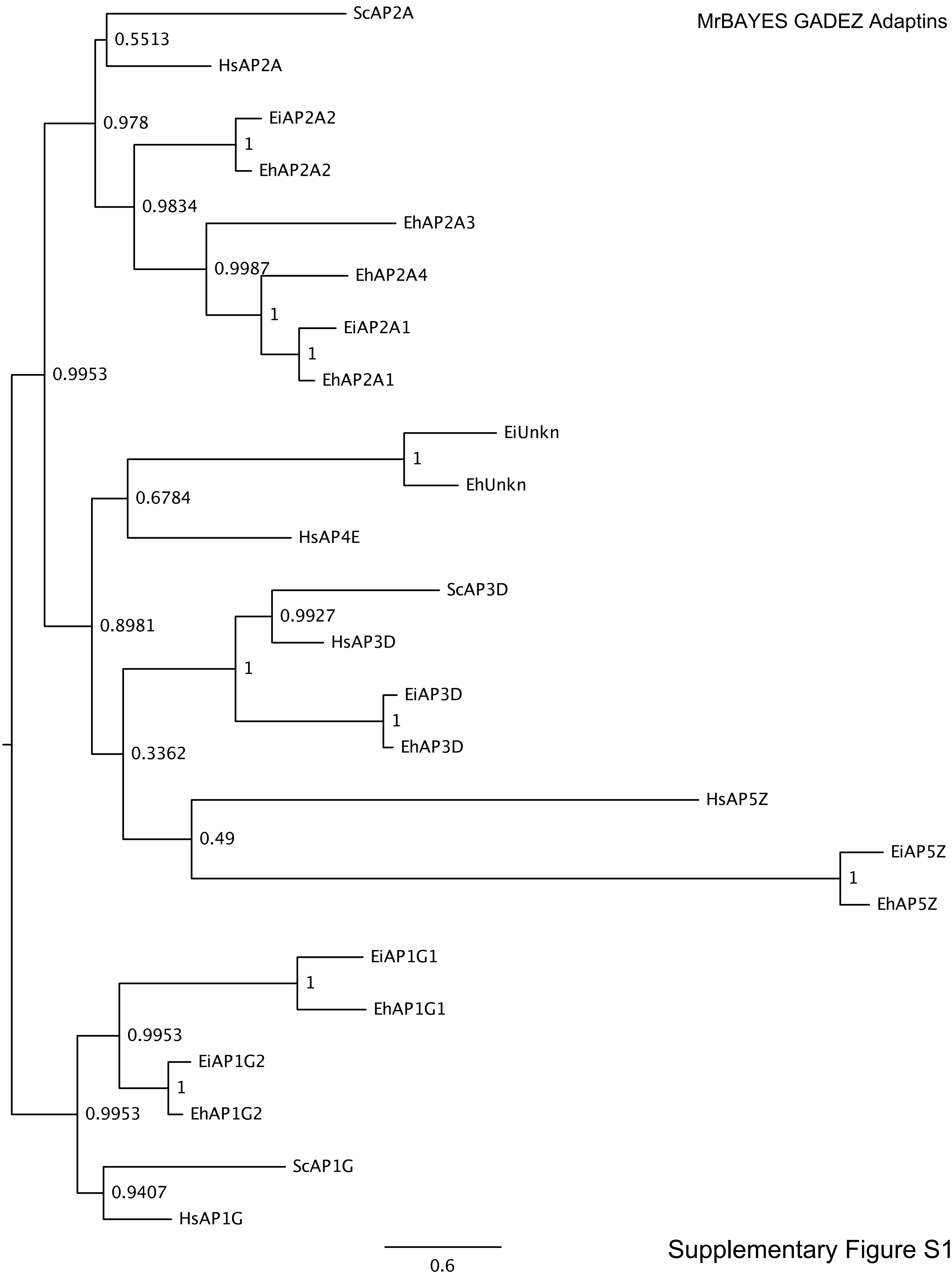

Supplementary Figure S1A

Phylobayes GADEZ Adaptins

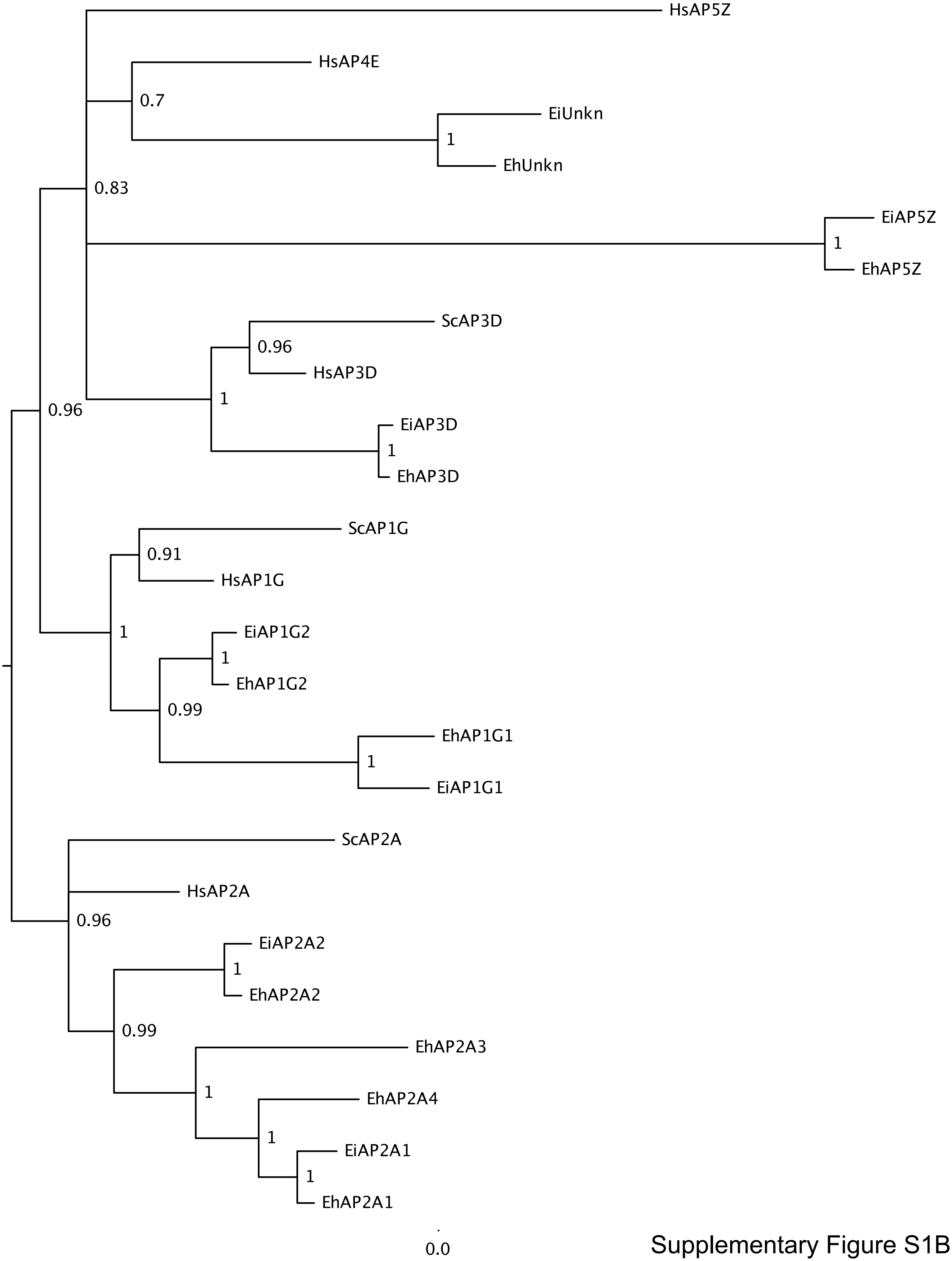

Supplementary Figure S1B

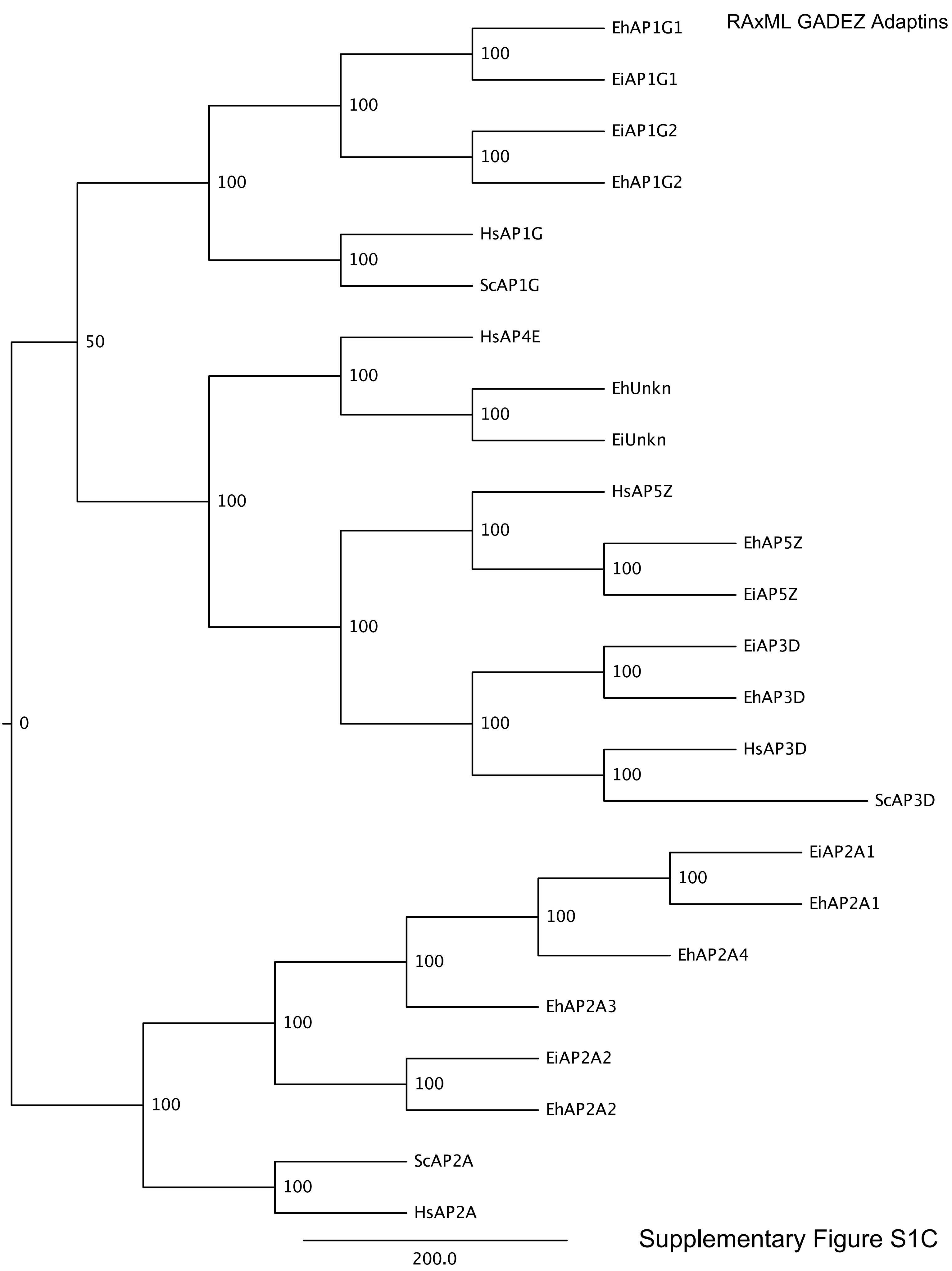

Supplementary Figure S1C

MrBAYES ArfGAP

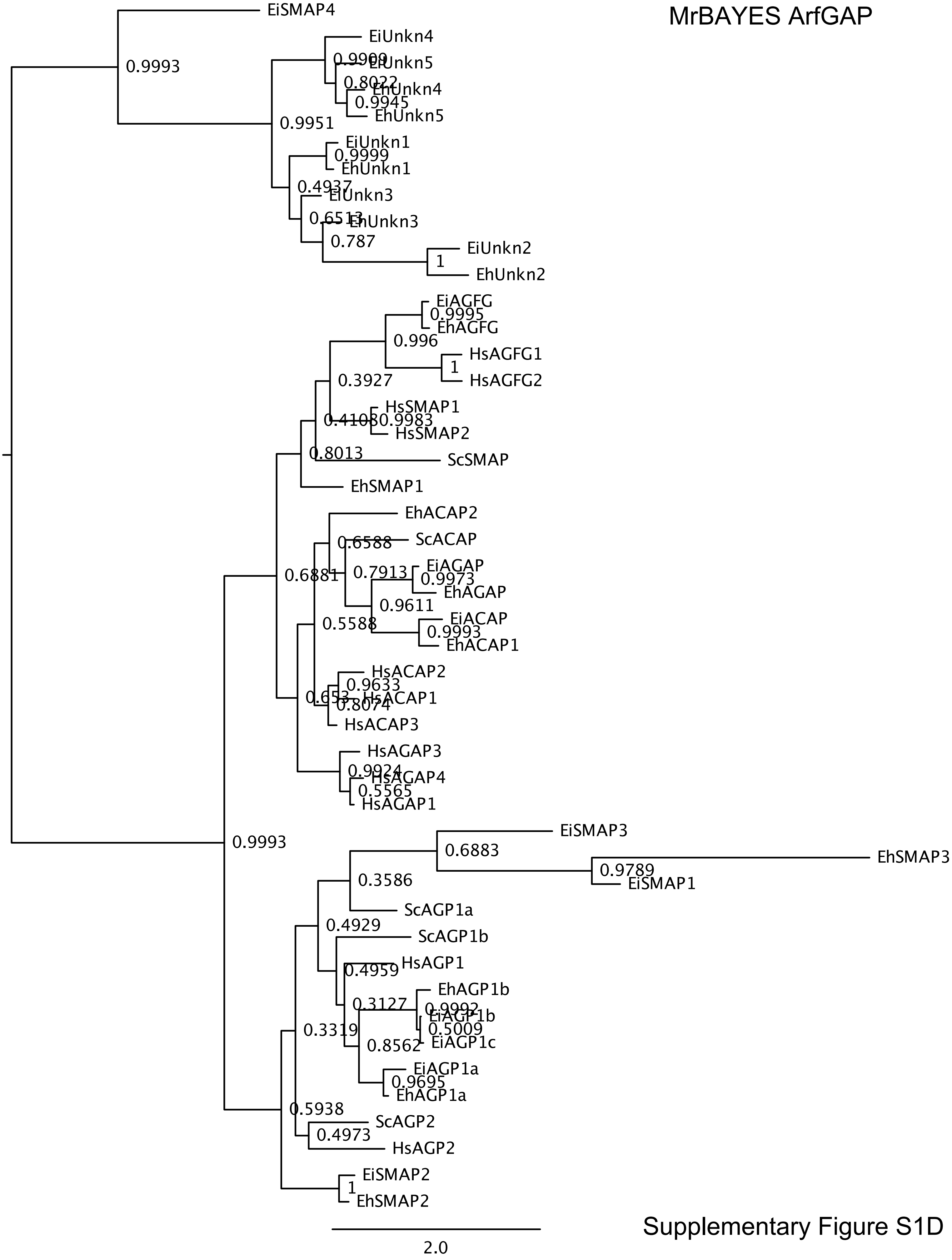

Supplementary Figure S1D

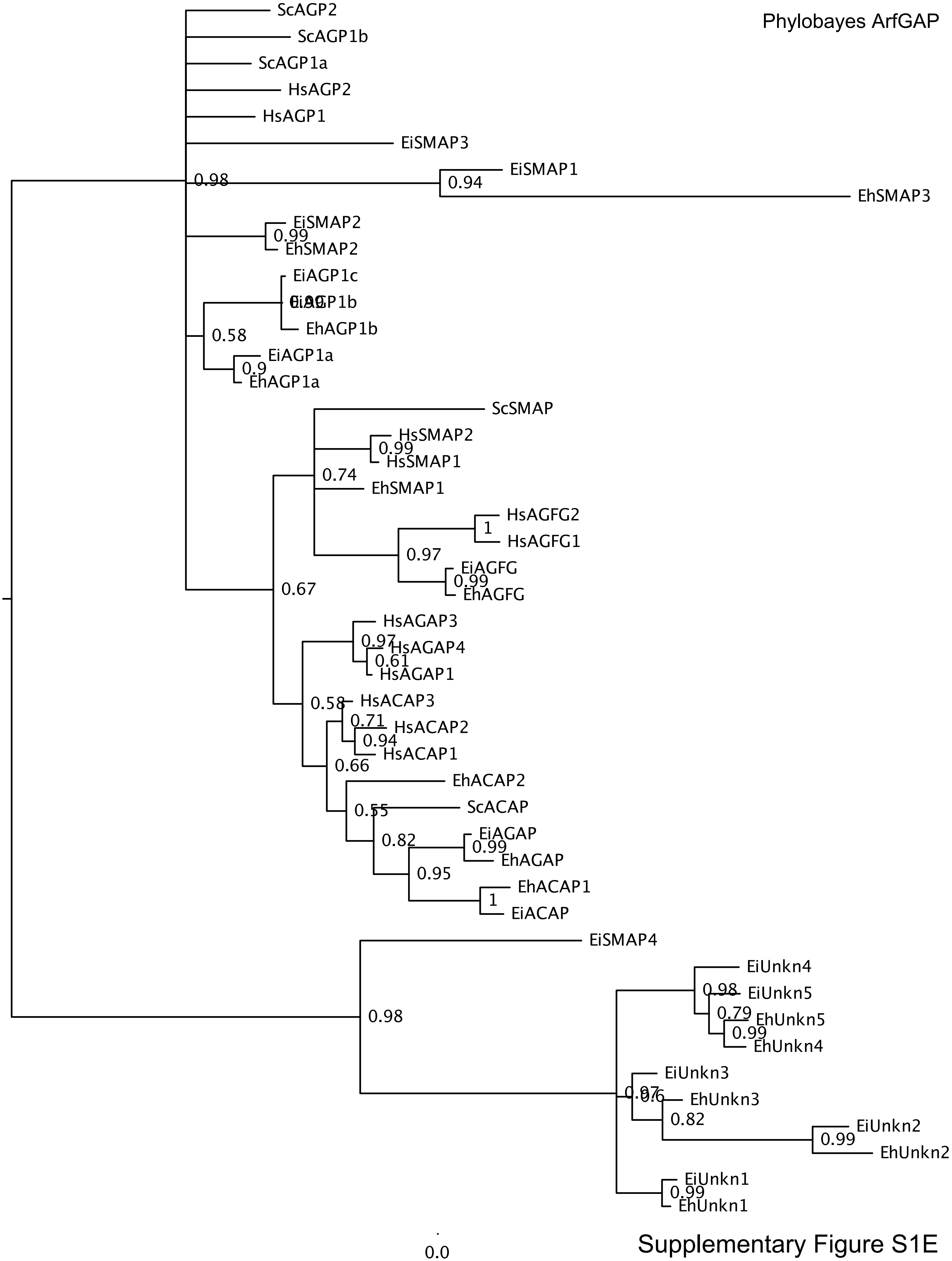

Supplementary Figure S1E

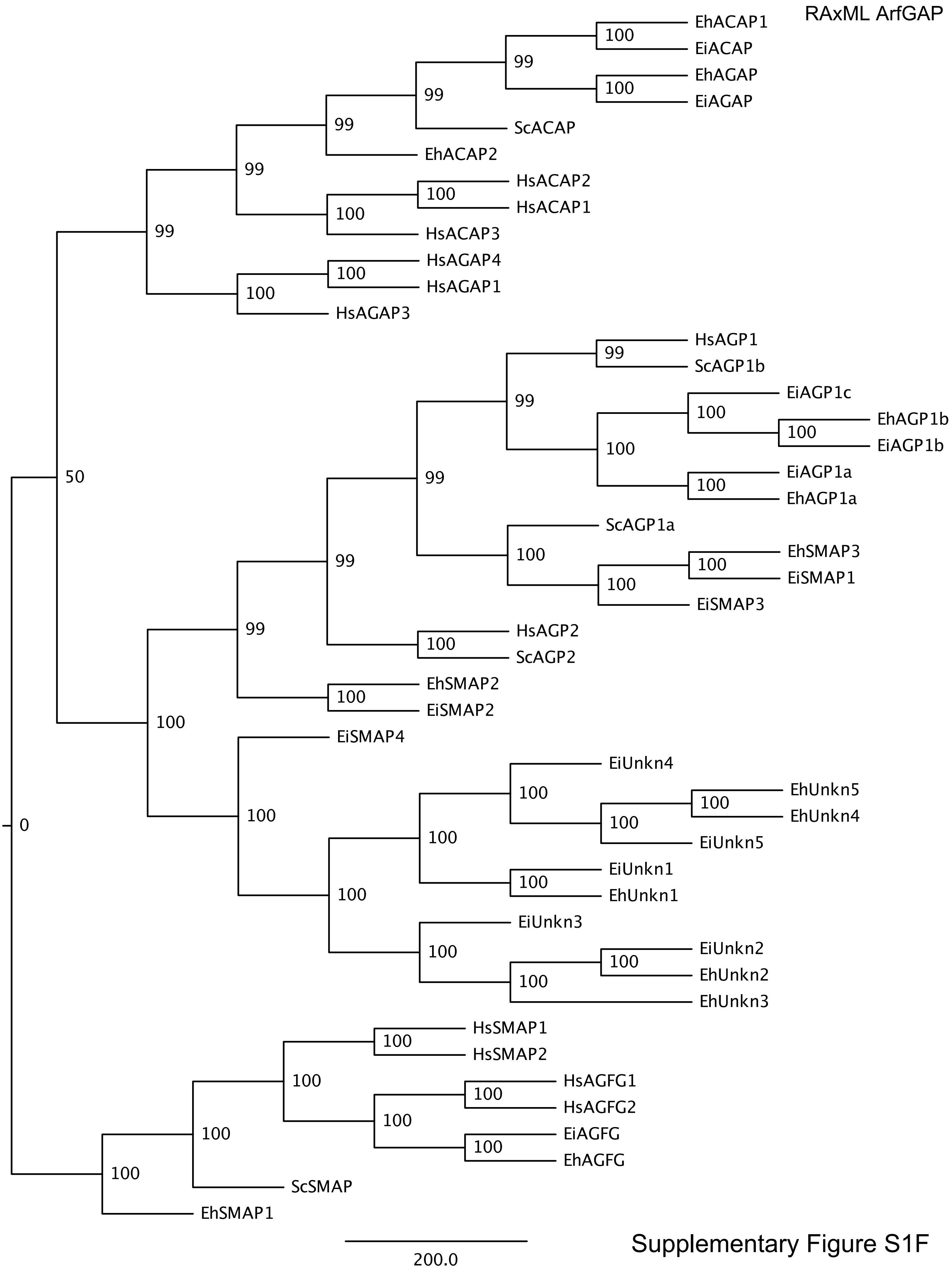

Supplementary Figure S1F

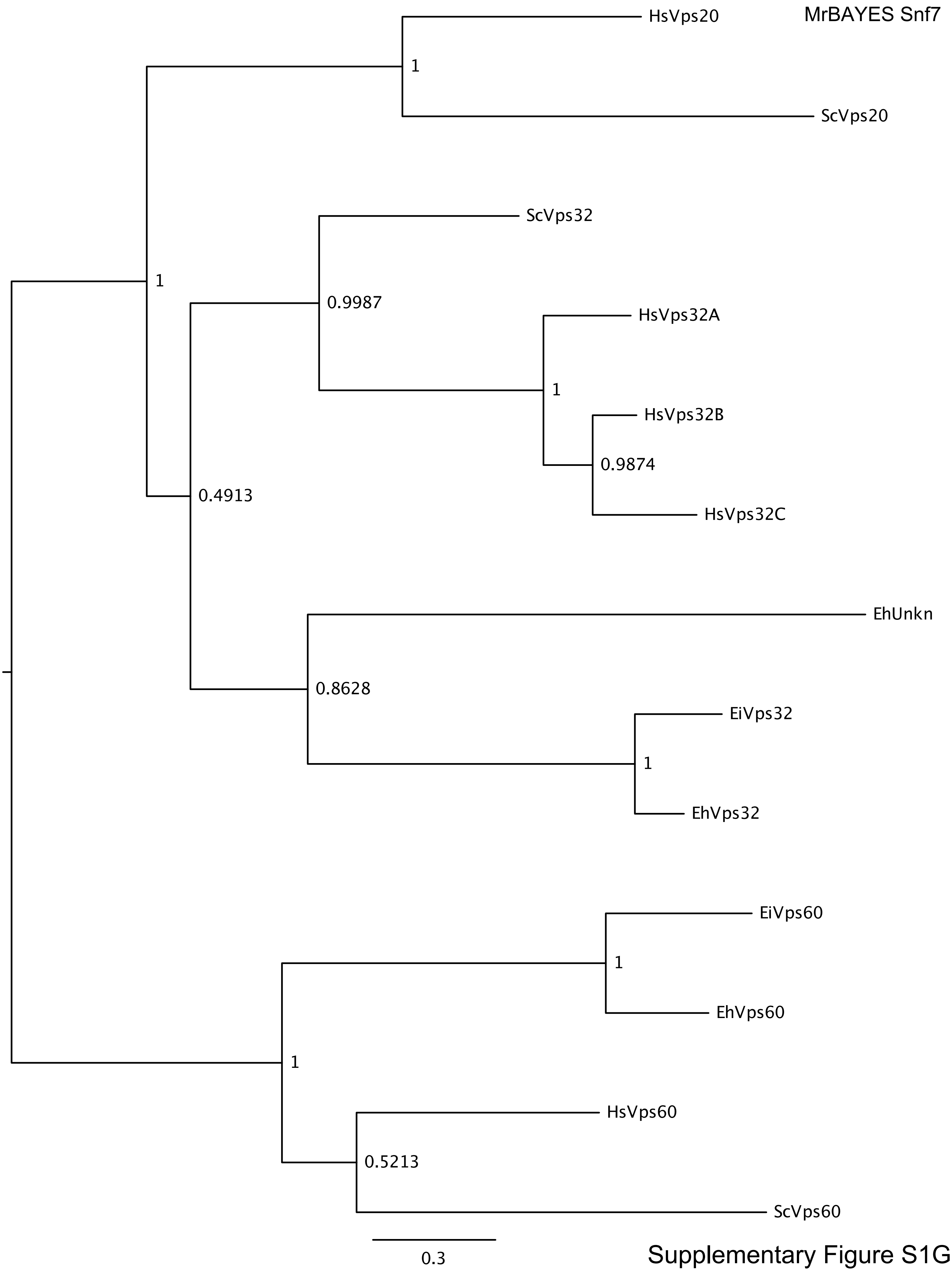

Supplementary Figure S1G

Phylobayes Snf7

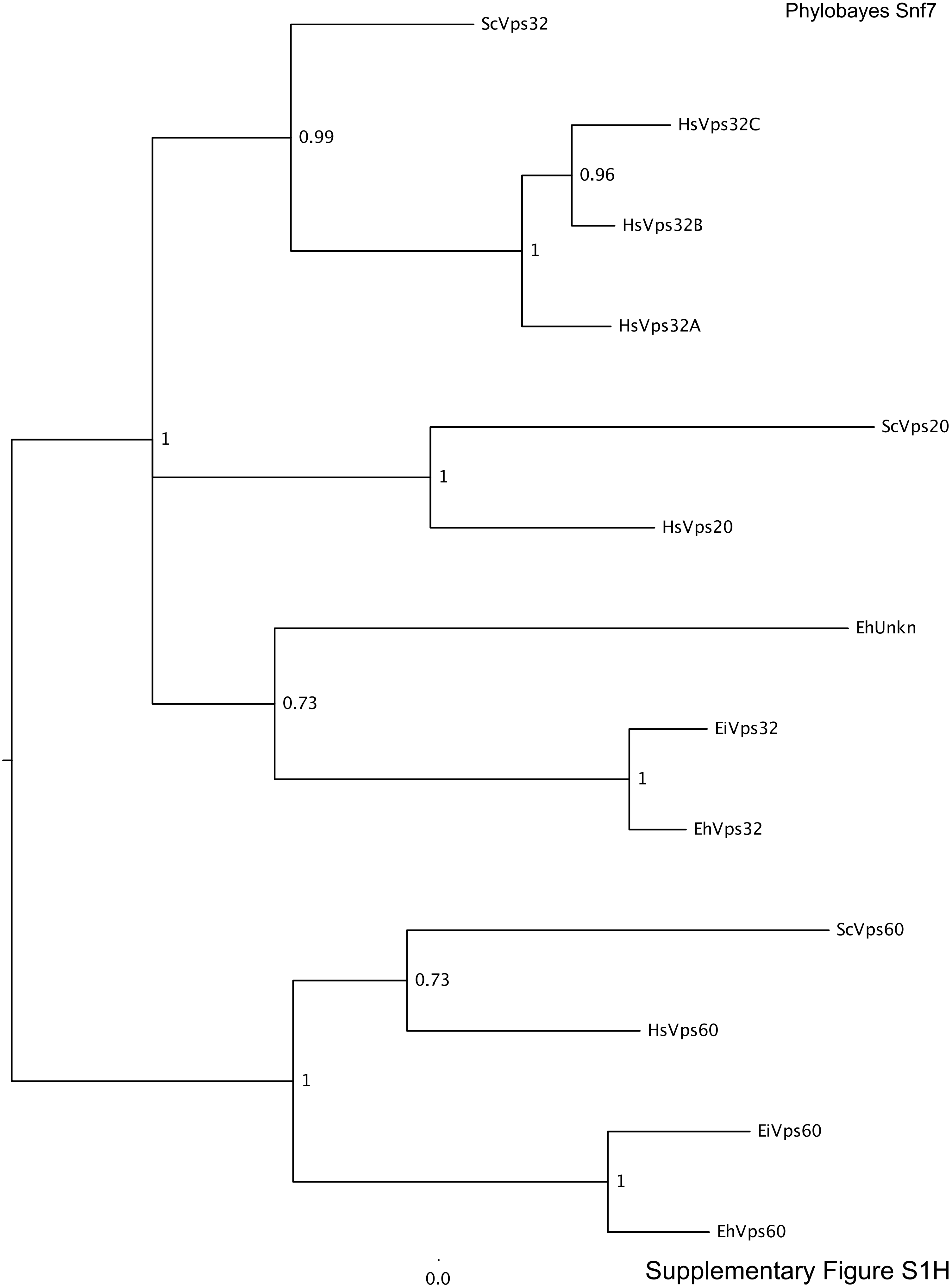

Supplementary Figure S1H

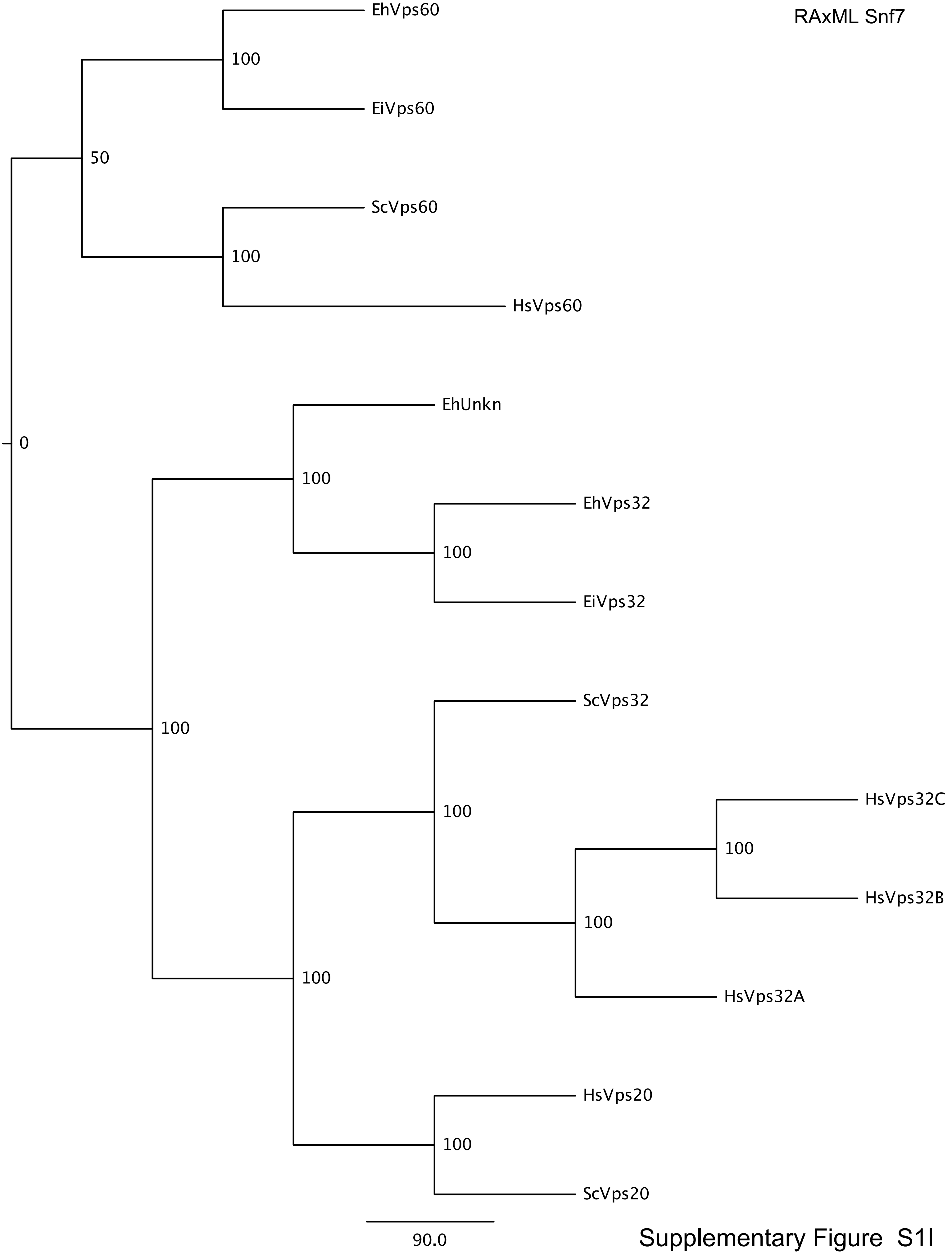

Supplementary Figure S1I

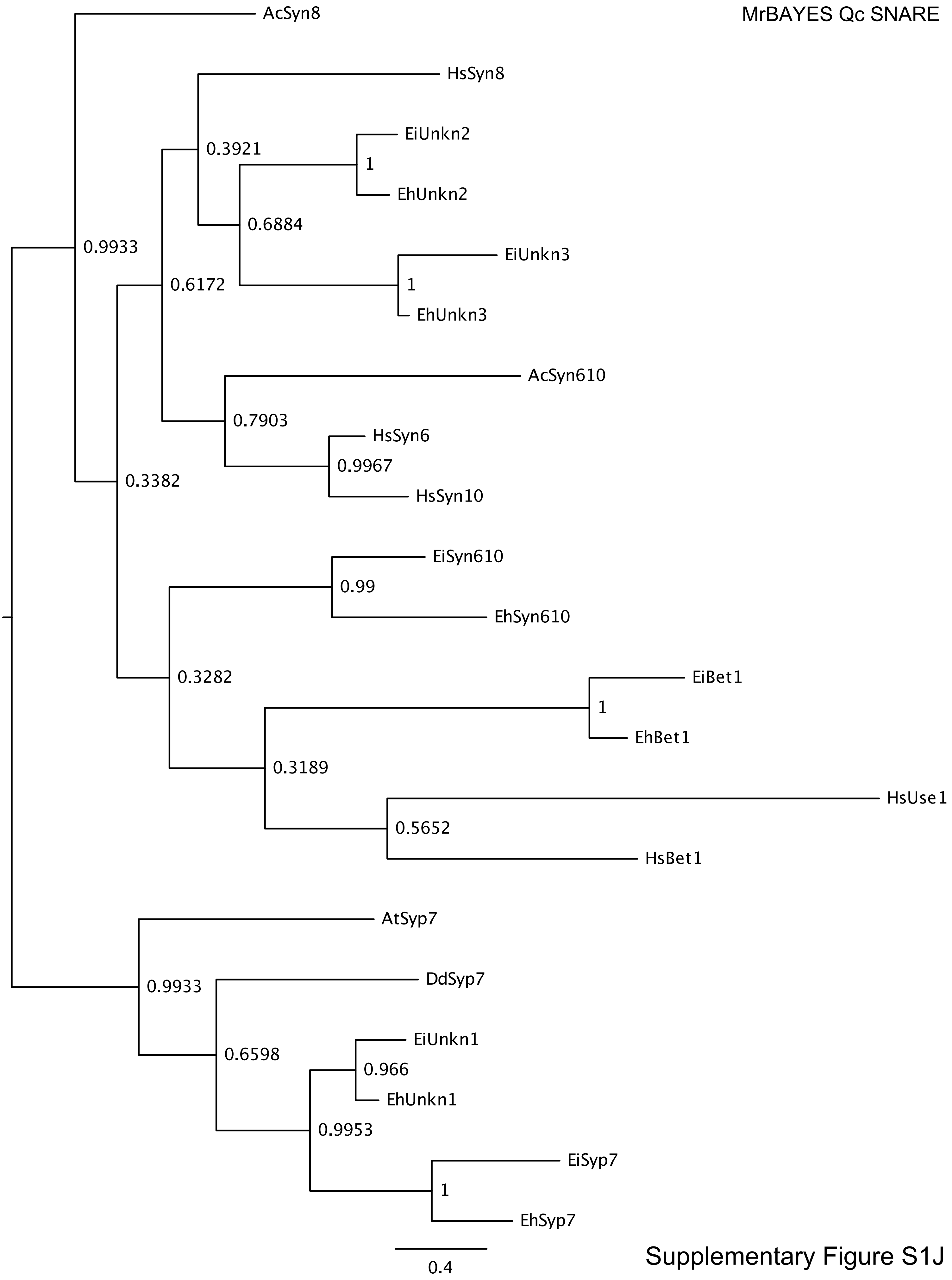

Supplementary Figure S1J

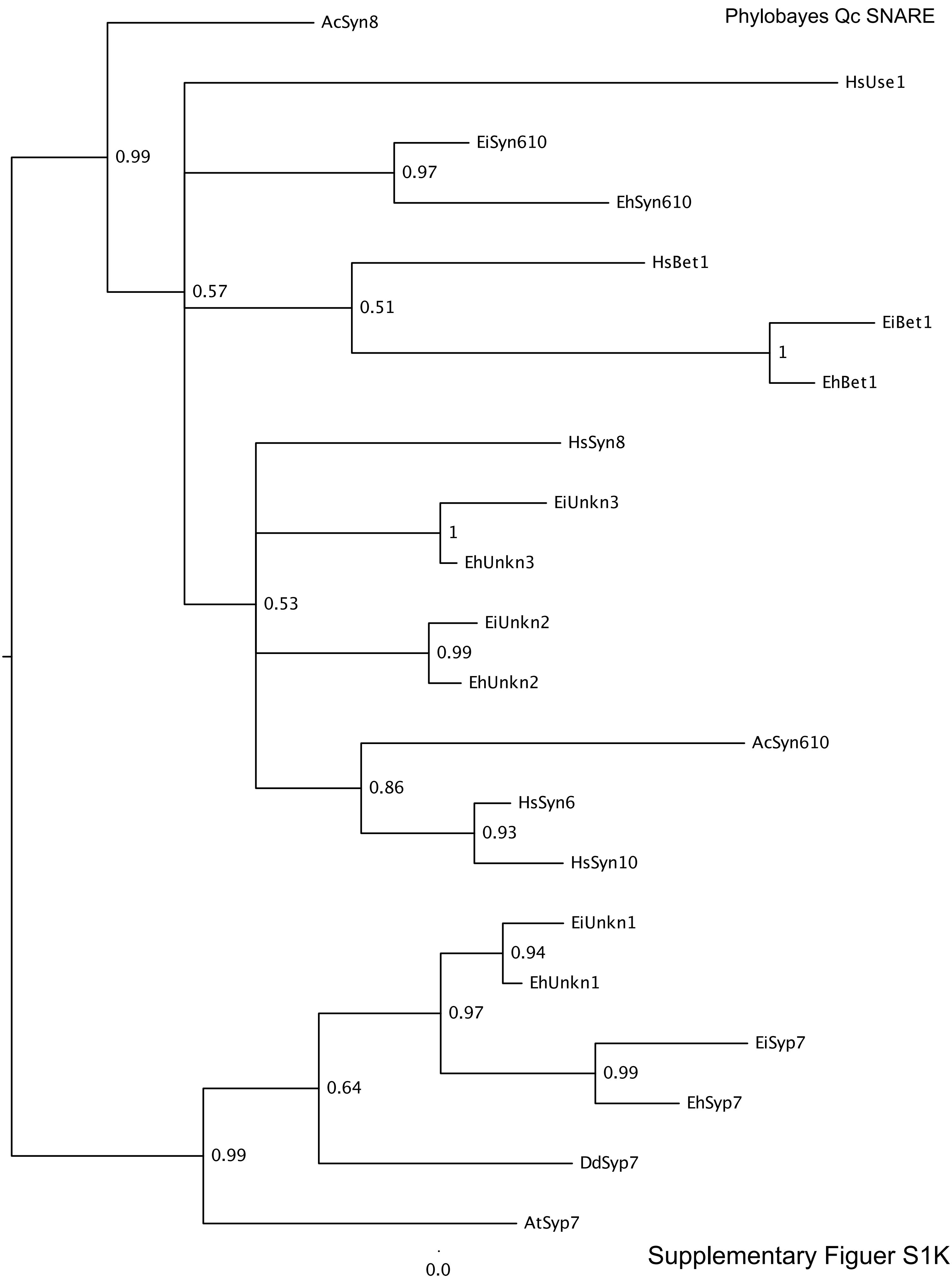

Supplementary Figuer S1K

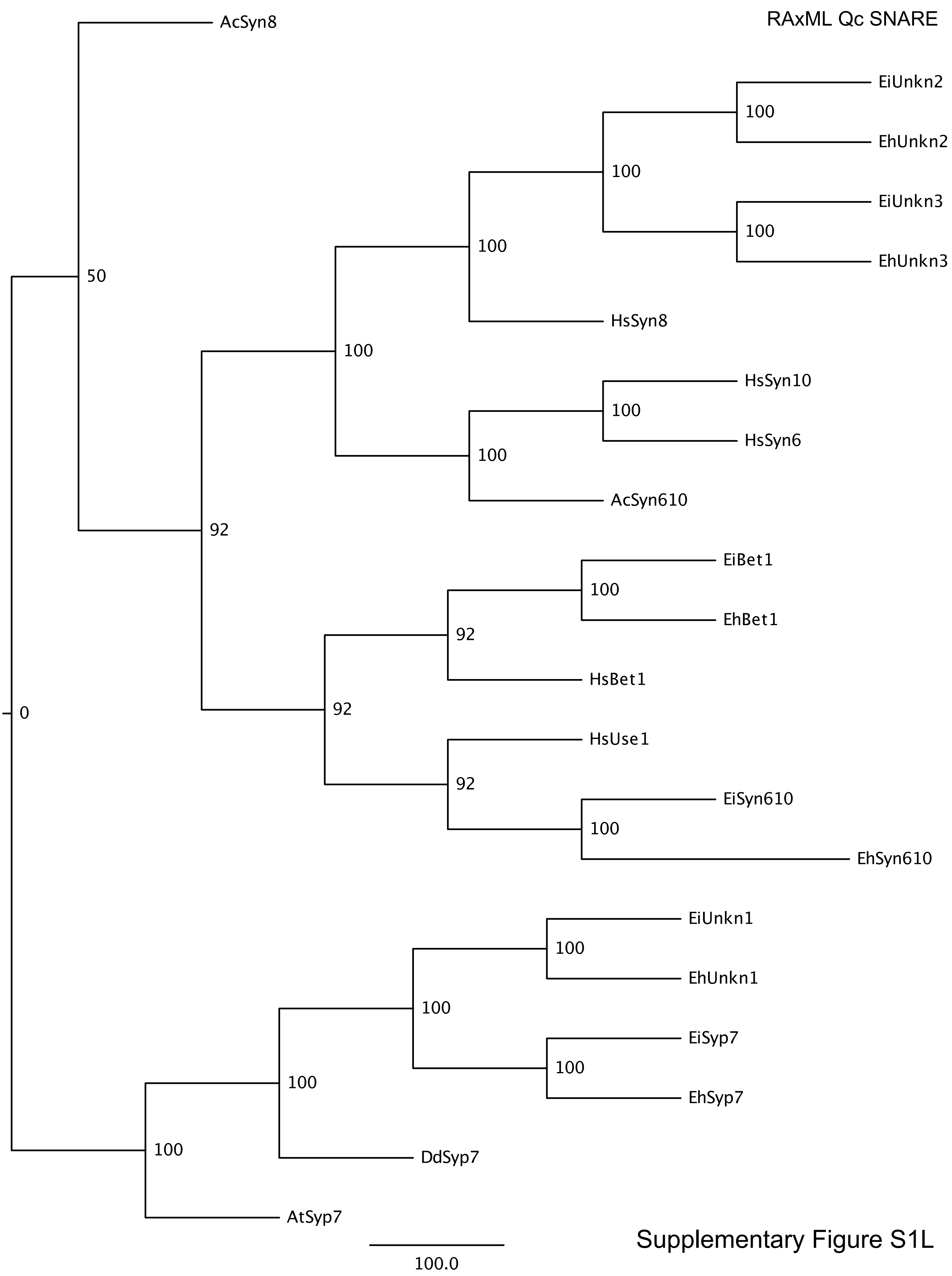

Supplementary Figure S1L

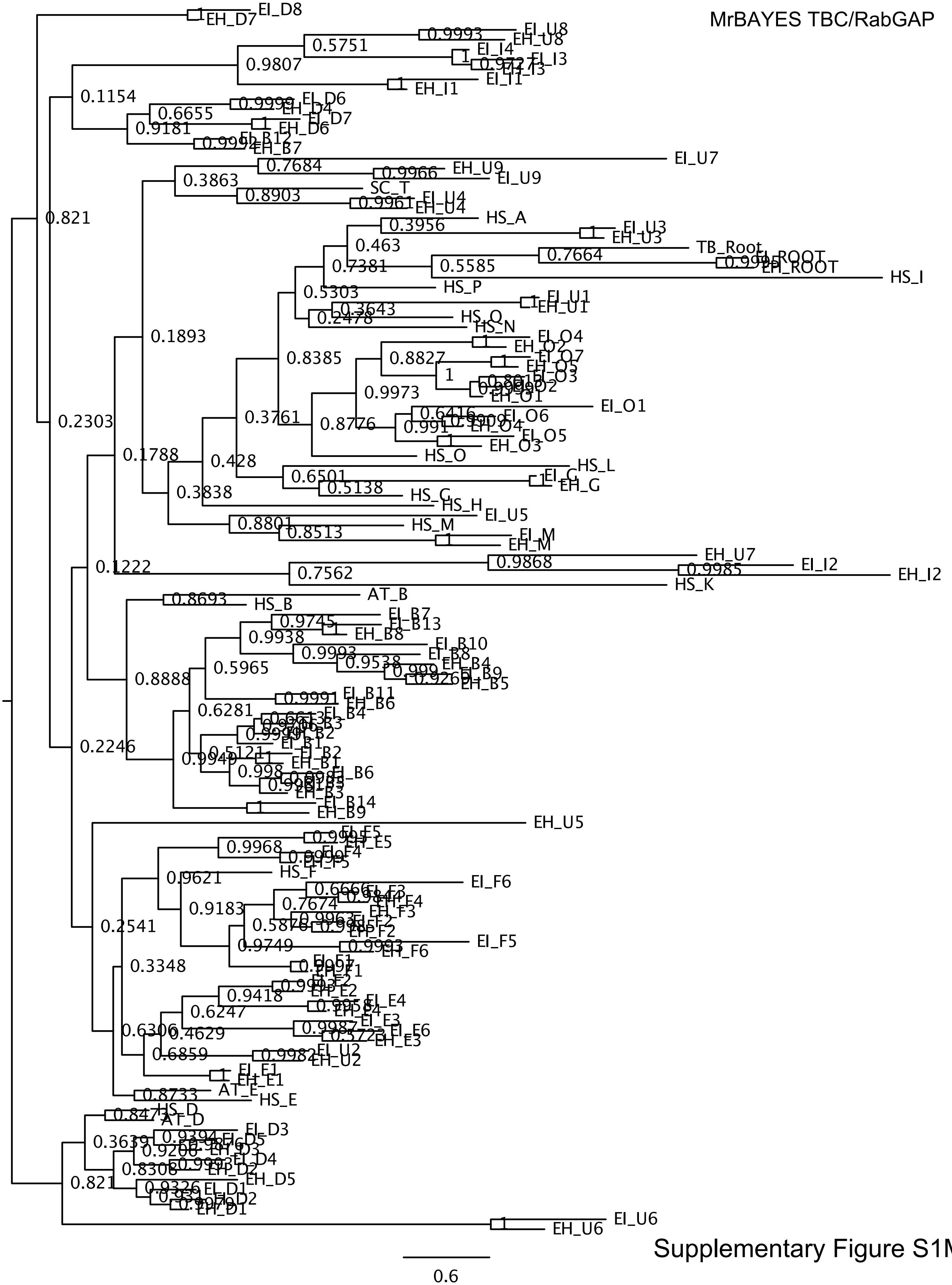

Supplementary Figure S1M

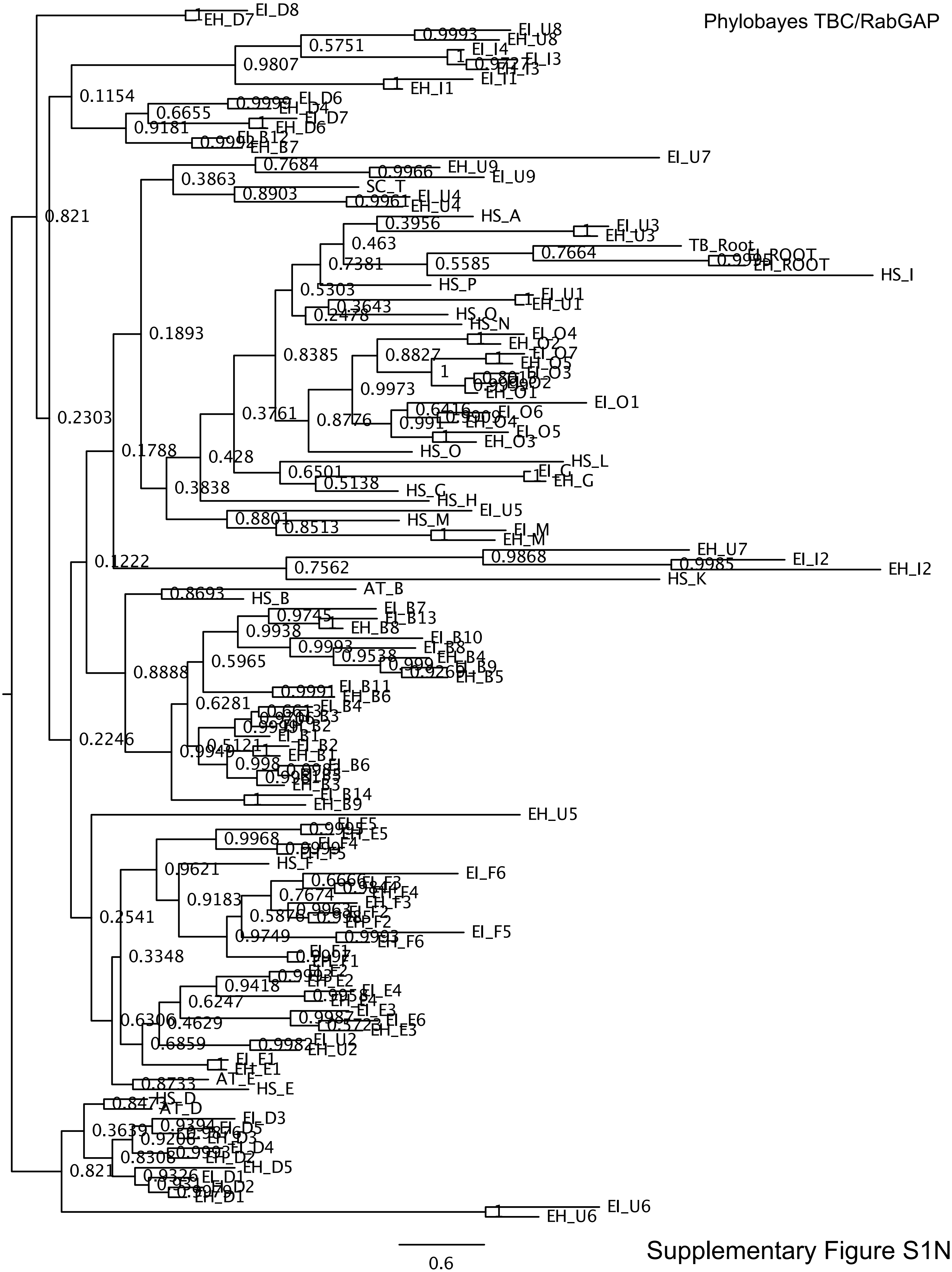

Supplementary Figure S1N

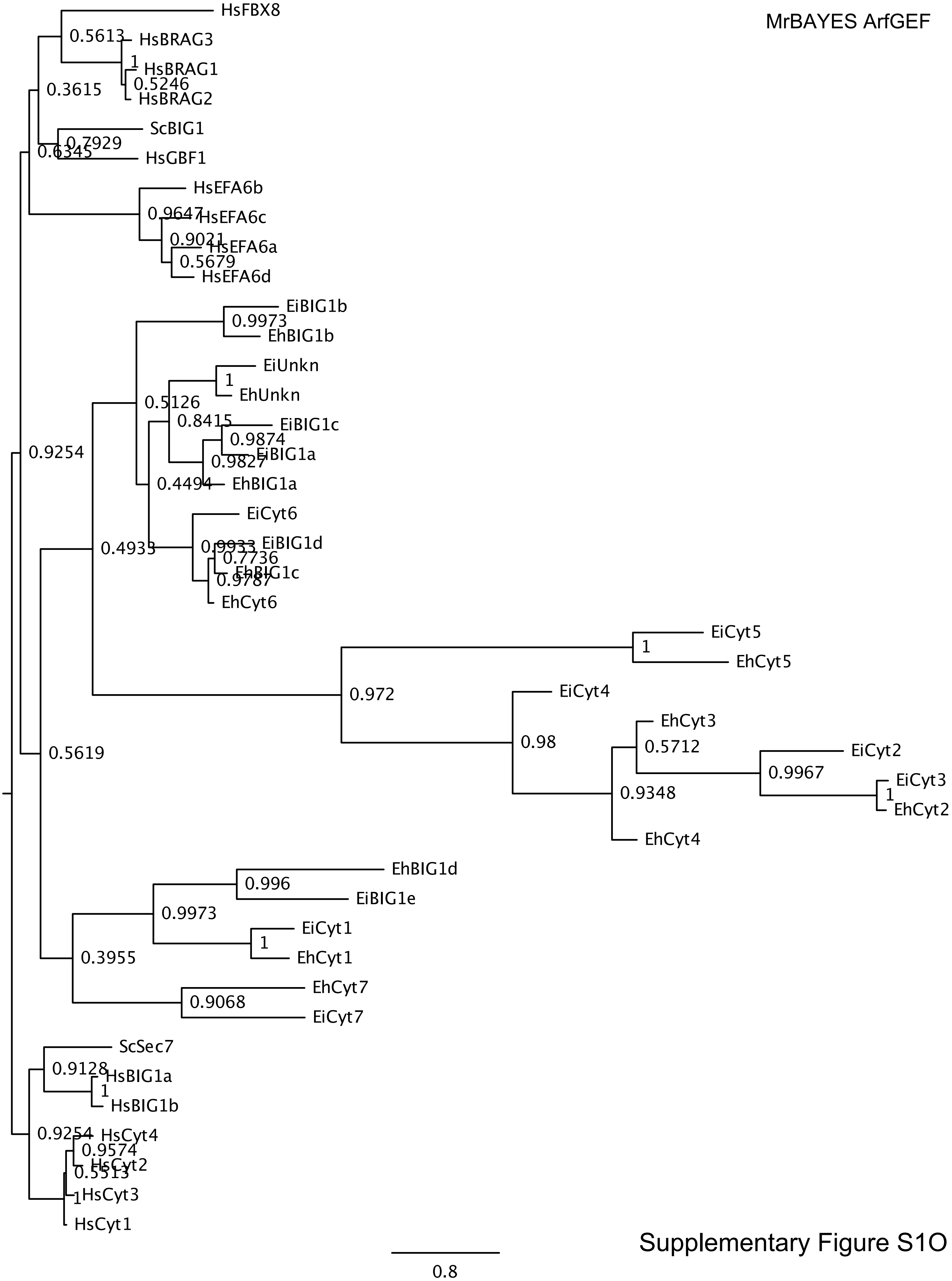

Supplementary Figure S1O

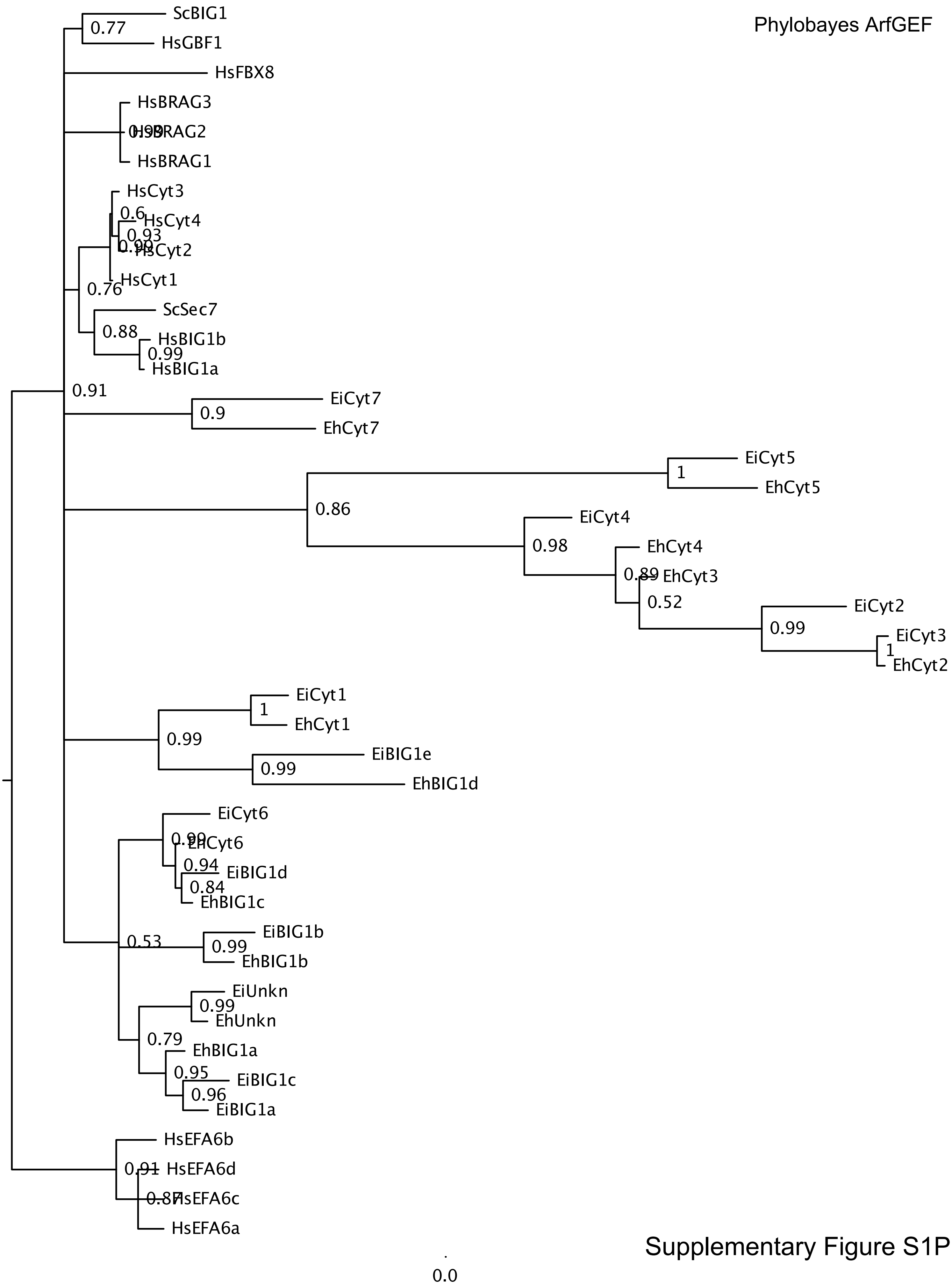

Supplementary Figure S1P

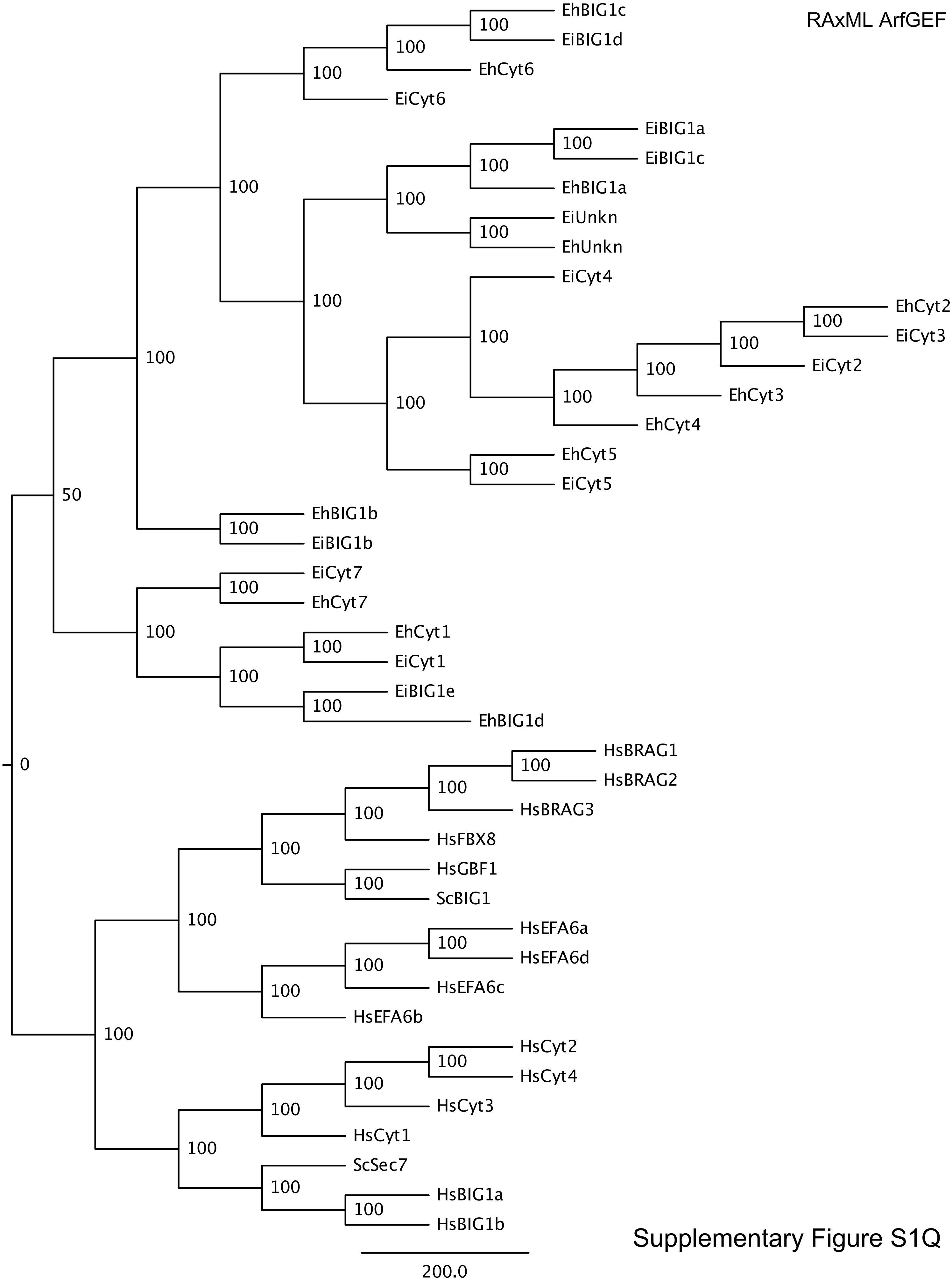

Supplementary Figure S1Q

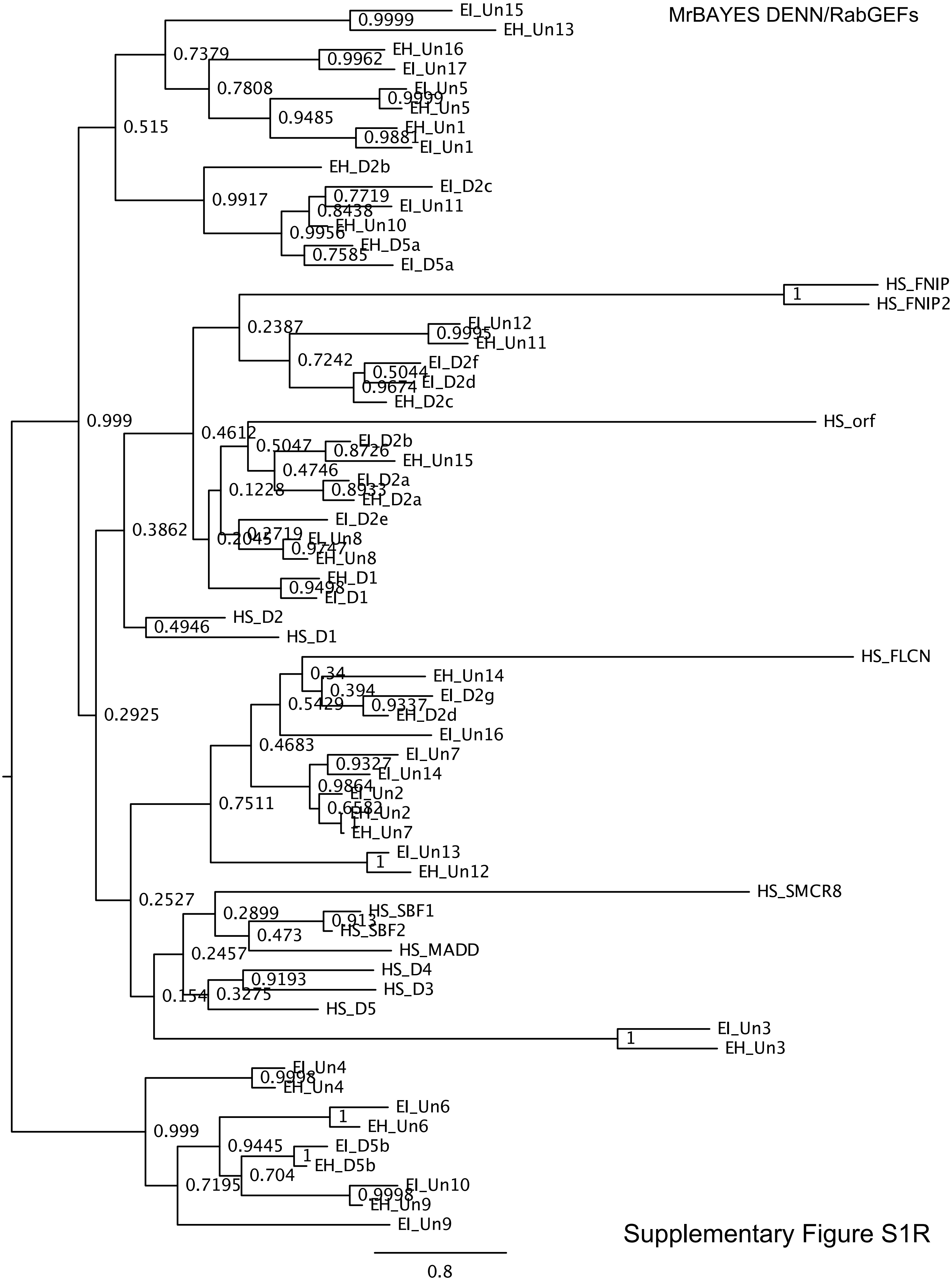

Supplementary Figure S1R

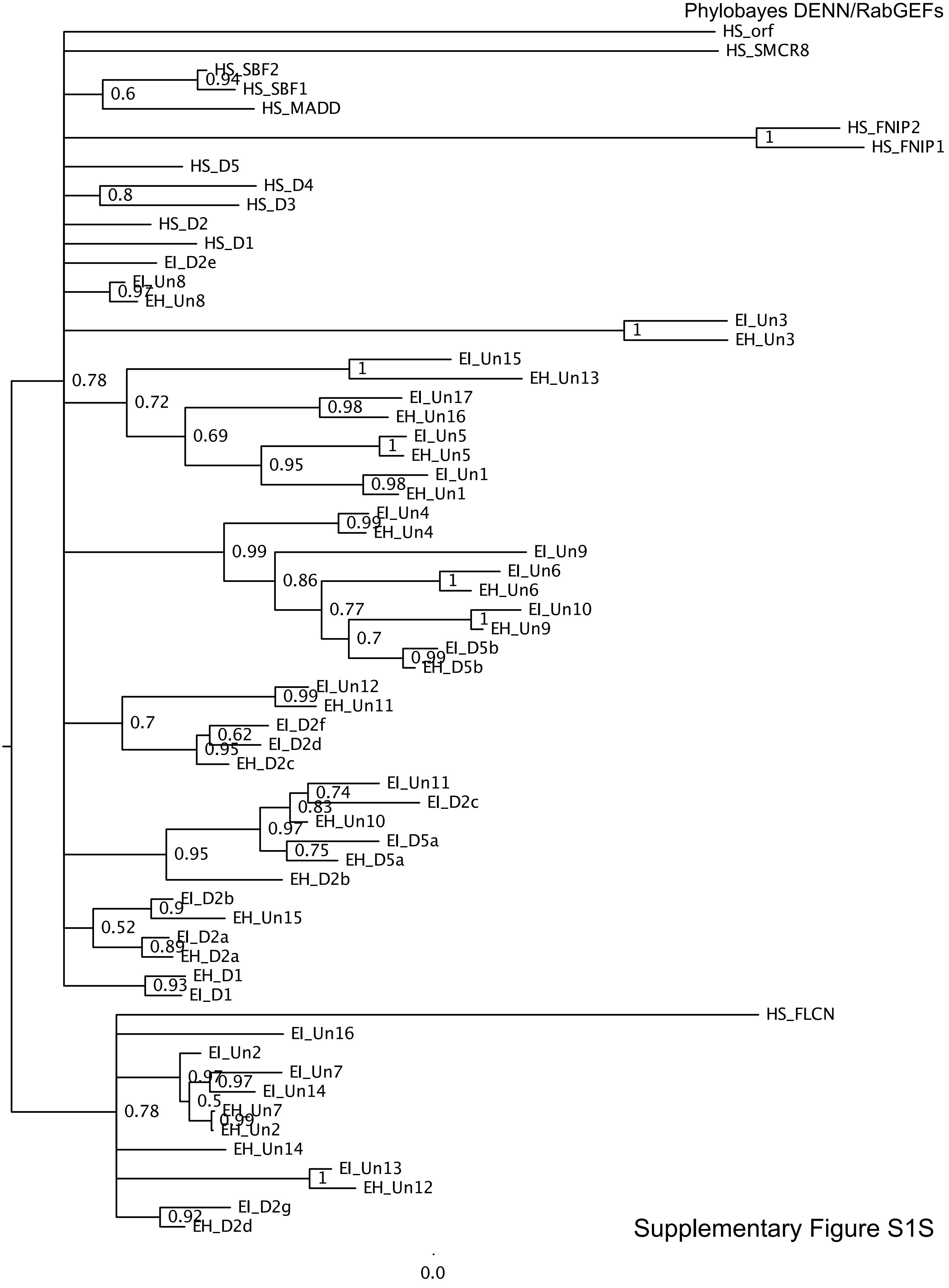

Supplementary Figure S1S

RAxML DENN/RabGEFs

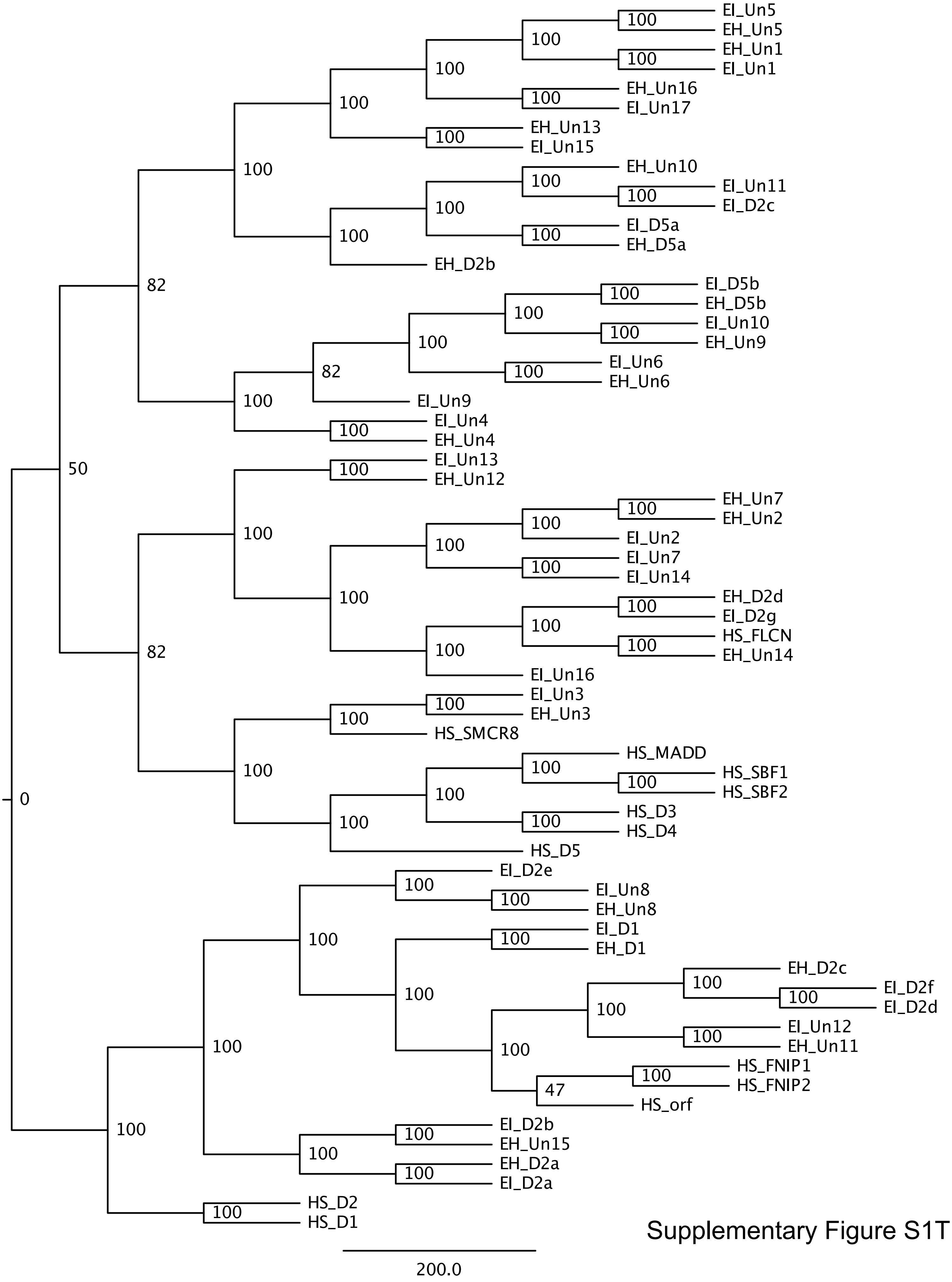

Supplementary Figure S1T

MrBAYES Arf GTPase

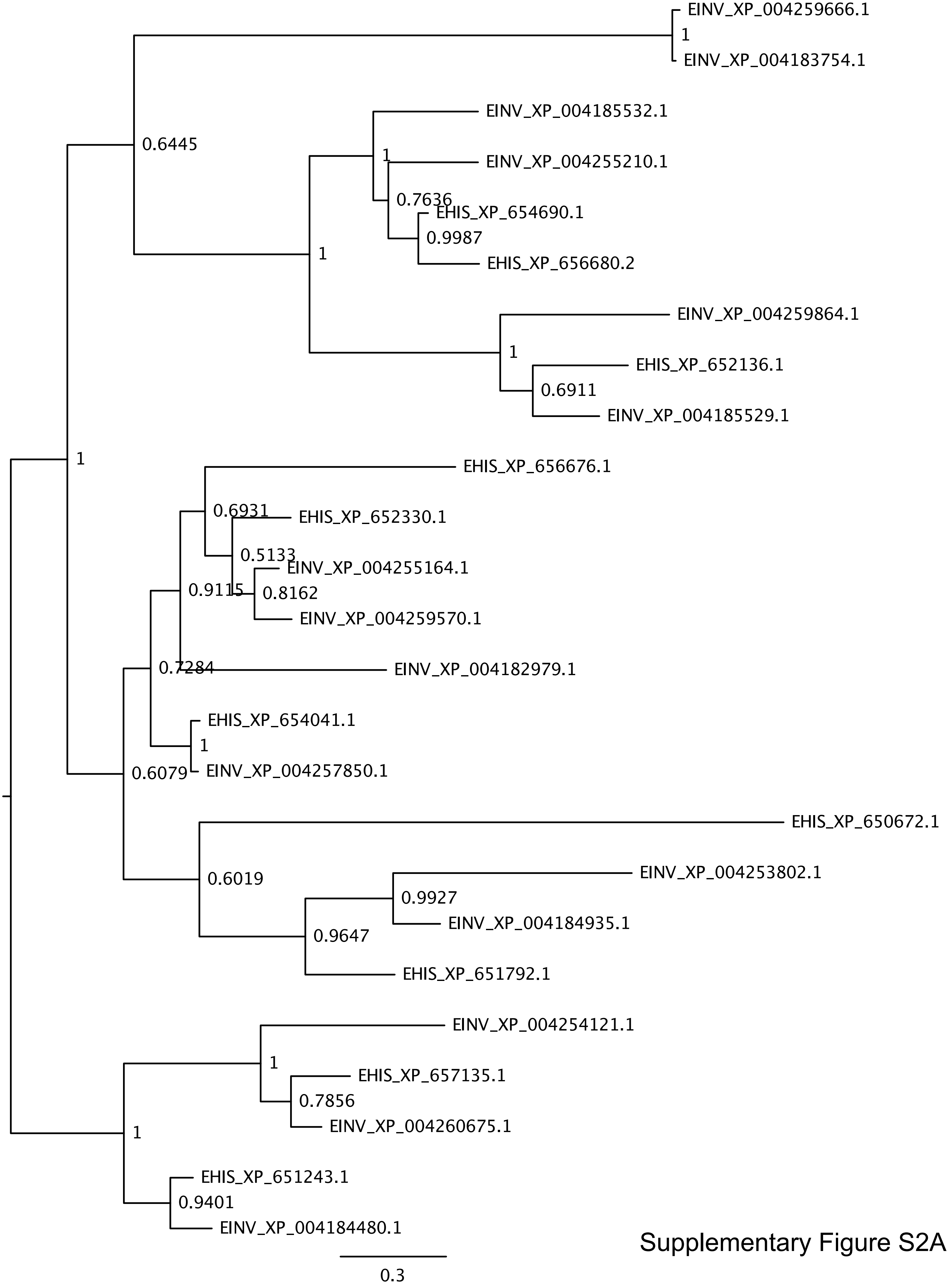

Supplementary Figure S2A

RAxML Arf GTPase

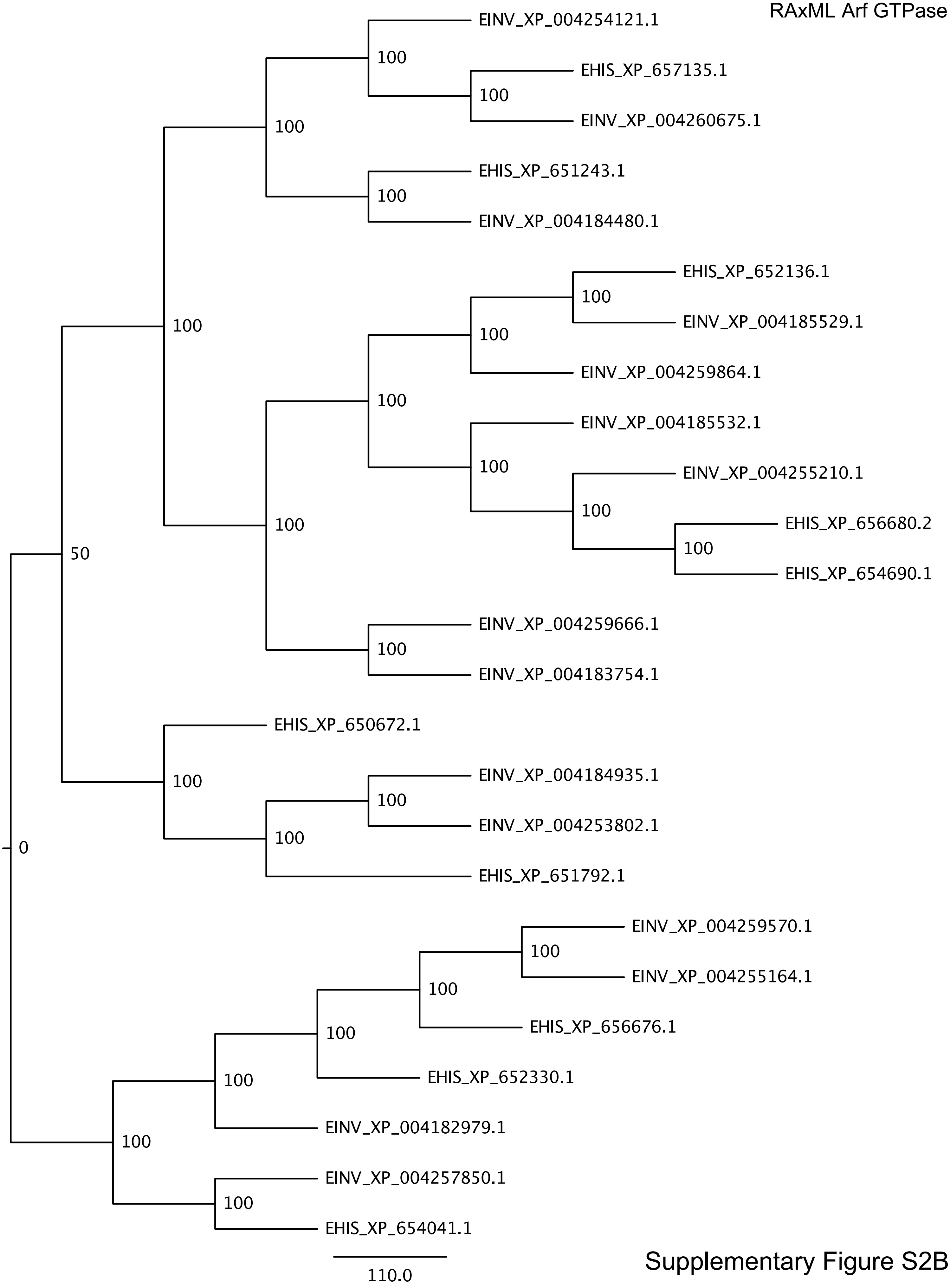

Supplementary Figure S2B

Bioplot of gene expression cluster principal components

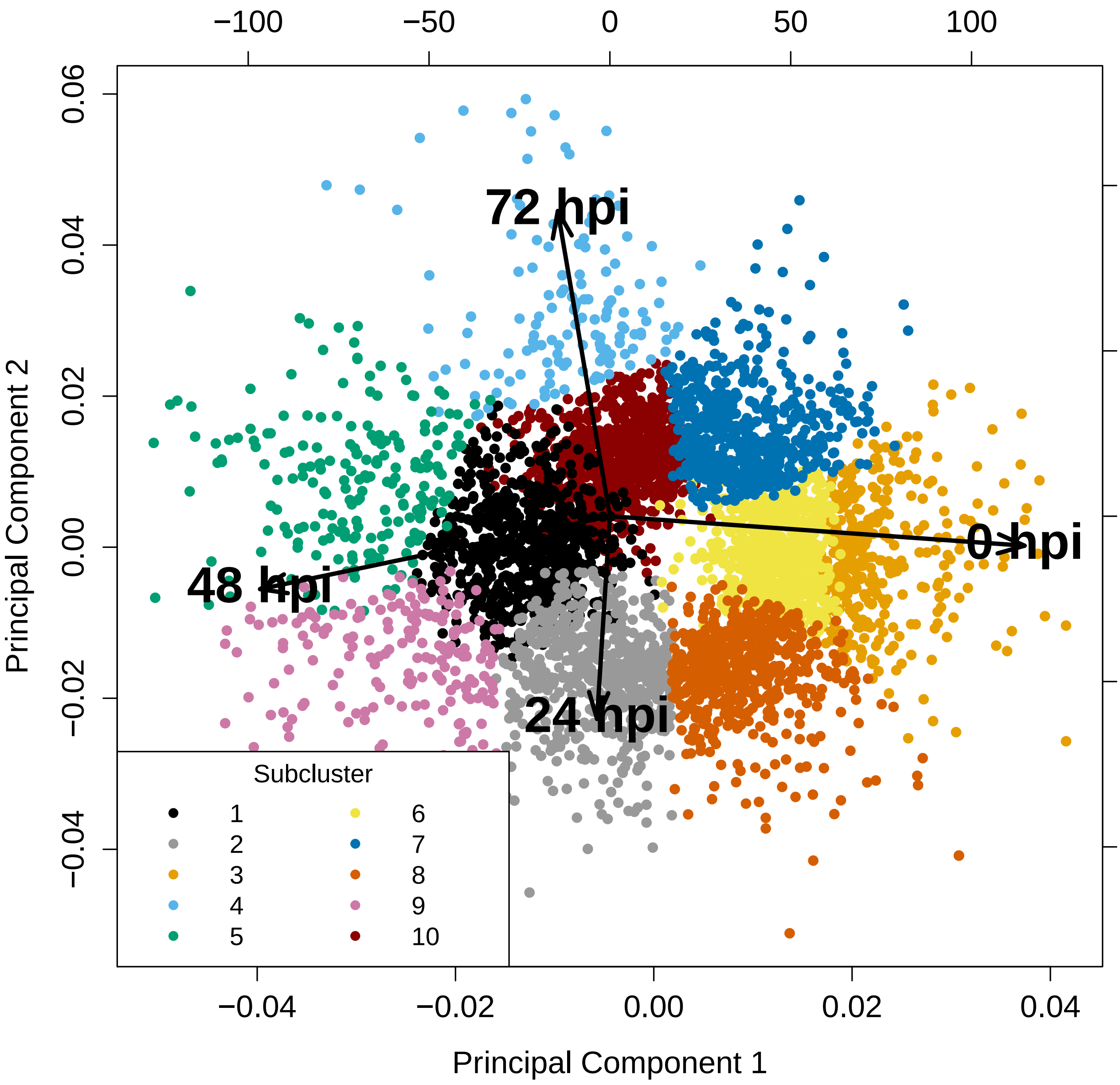

Supplementary Figure S3

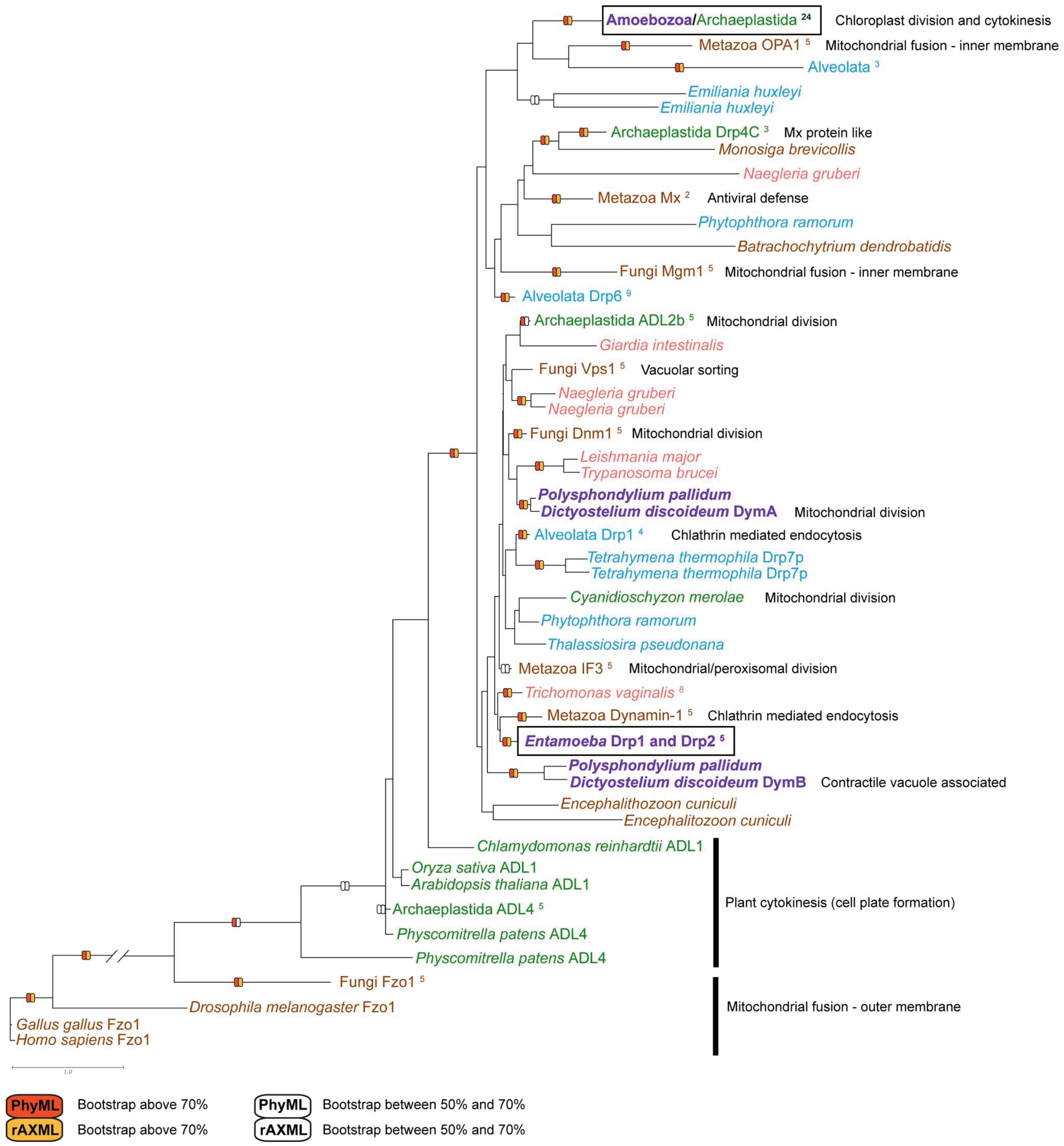

Supplementary Figure S4

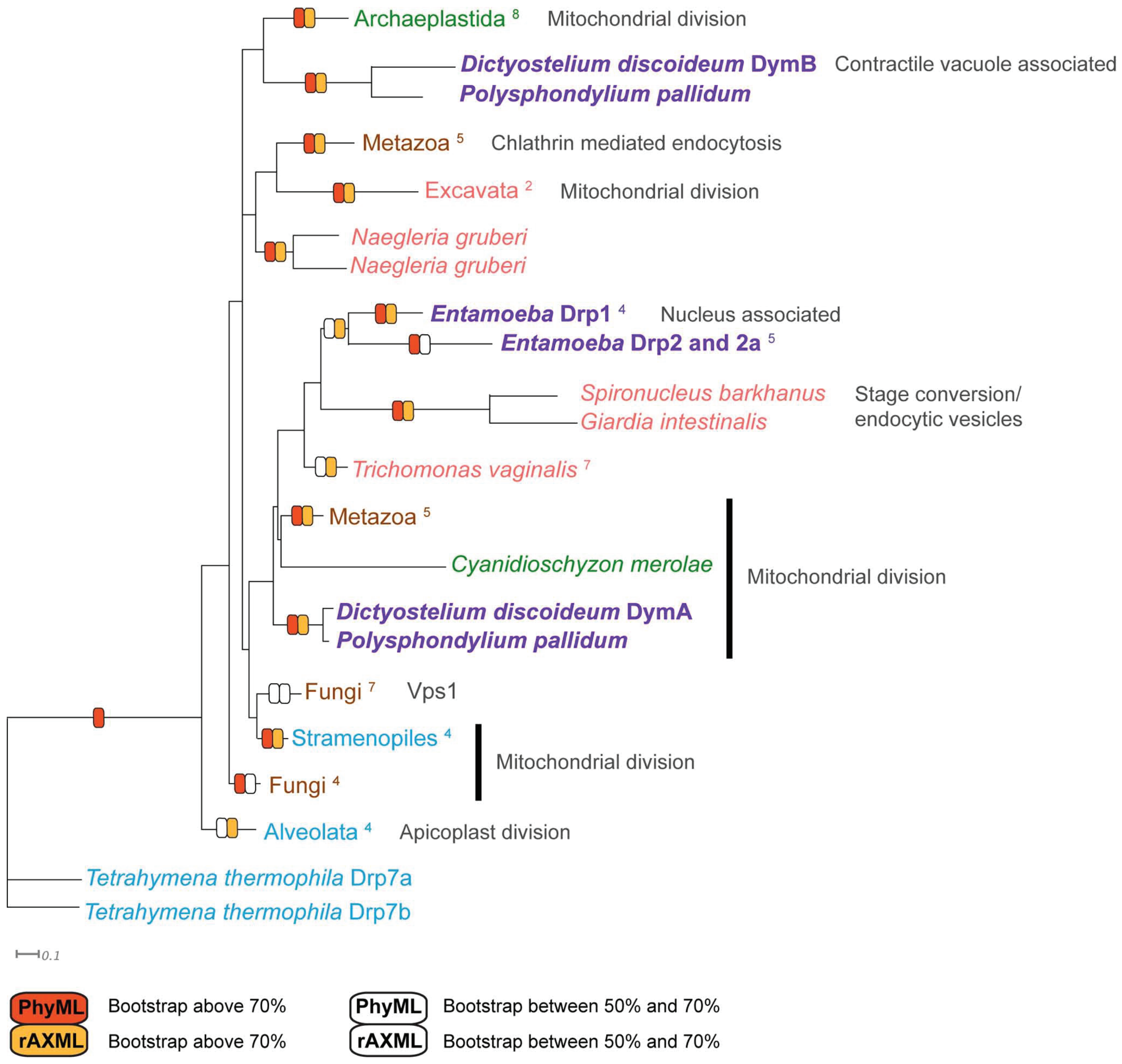

Supplementary Figure S5

Cyst formation in *Entamoeba invadens*

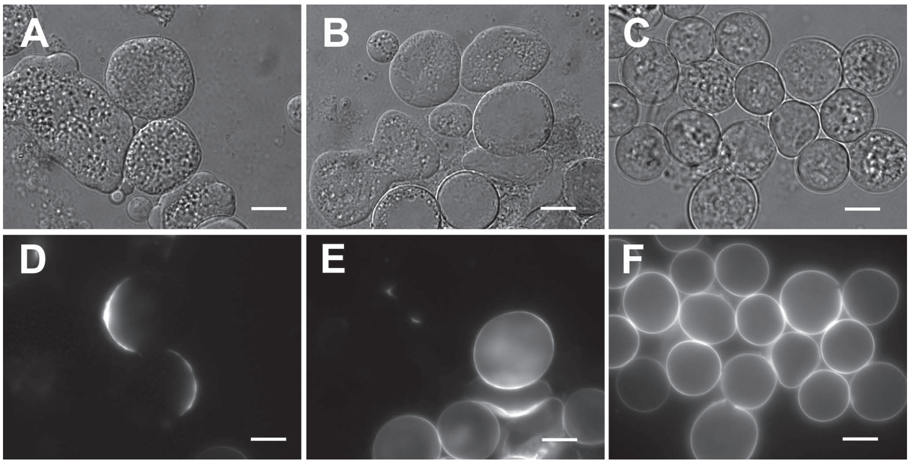

Supplementary Figure S6

Semi-quantitative RT-PCR analysis of *E. invadens* chitinase expression during encystation

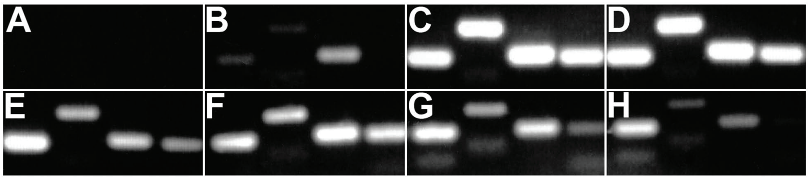

Supplementary Figure S7

Coomassie stained gel and Western blot analysis of total *E. invadens* protein using *Dictyostelium discoidum* anti-DlpA antibody

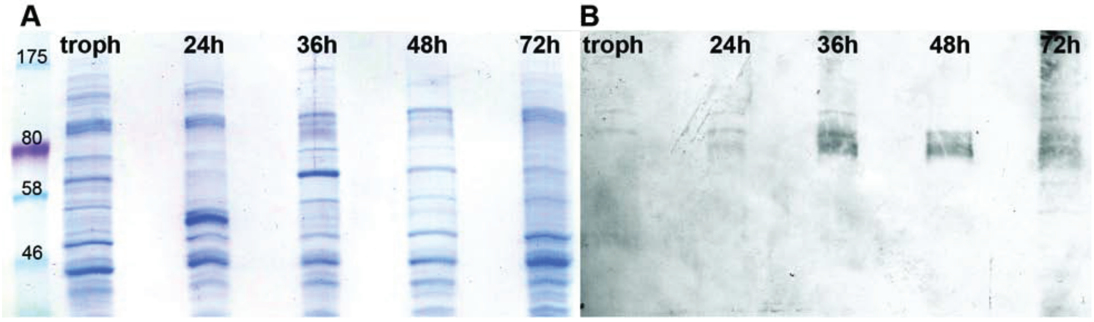

Supplementary Figure S8

Subcellular localization of Drp3 and Drp4

Drp3																
	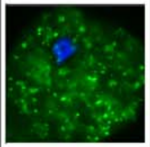		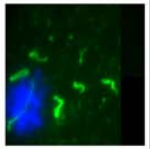		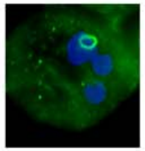		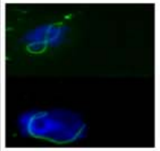		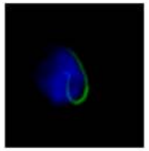		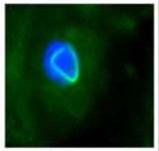		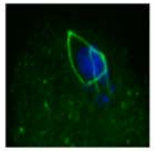		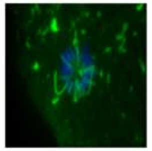	
Time point	No.	%	No.	%	No.	%	No.	%	No.	%	No.	%	No.	%	No.	%
0 h	67	56	28	24	20	17	0	0	0	0	3	2.5	1	0.8	0	0
24 h	54	46	30	25	16	14	3	2.5	13	11	2	1.5	0	0	0	0
28 h	38	25	23	15	21	14	12	8	24	16	18	12	14	9	2	1.5
32 h	13	9	25	16	32	21	11	7	37	24	26	17	8	5	0	0
36 h	18	14	31	24	14	11	0	0	16	12	30	23	12	9	8	6
40 h	25	21	28	24	14	12	0	0	29	25	10	8	11	9	1	0.8
44 h	21	16	42	32	20	15	0	0	22	17	11	8	15	11	0	0
72 h	11	9	8	7	17	14	15	12	38	31	16	13	17	14	1	0.8

Drp4																
	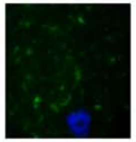		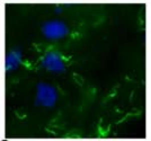		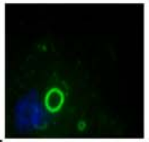		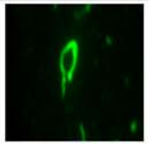		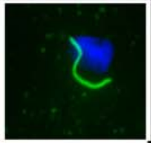		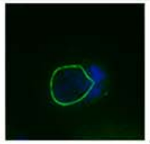		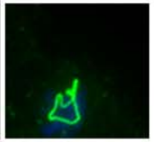		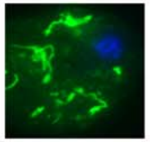	
Time point	No.	%	No.	%	No.	%	No.	%	No.	%	No.	%	No.	%	No.	%
0 h	24	20	60	49	23	19	7	6	8	7	1	0.8	0	0	0	0
24 h	27	21	56	43	15	12	5	4	19	15	3	2	1	0.7	3	2
28 h	16	13	33	28	20	17	9	8	26	22	4	3	3	2.5	4	3
32 h	13	9	35	25	12	8	30	21	18	13	7	5	11	8	16	11
36 h	12	9	37	28	24	18	6	4.5	7	5	9	7	6	4.5	31	23
40 h	15	11	44	32	17	12	10	7	20	14	19	14	14	10	0	0
44 h	21	15	50	36	9	7	15	11	16	12	4	3	4	3	18	13
72 h	4	3	6	5	9	8	13	11	34	29	22	19	20	17	9	8

Supplementary Figure S9

Calcofluor staining of cyst walls

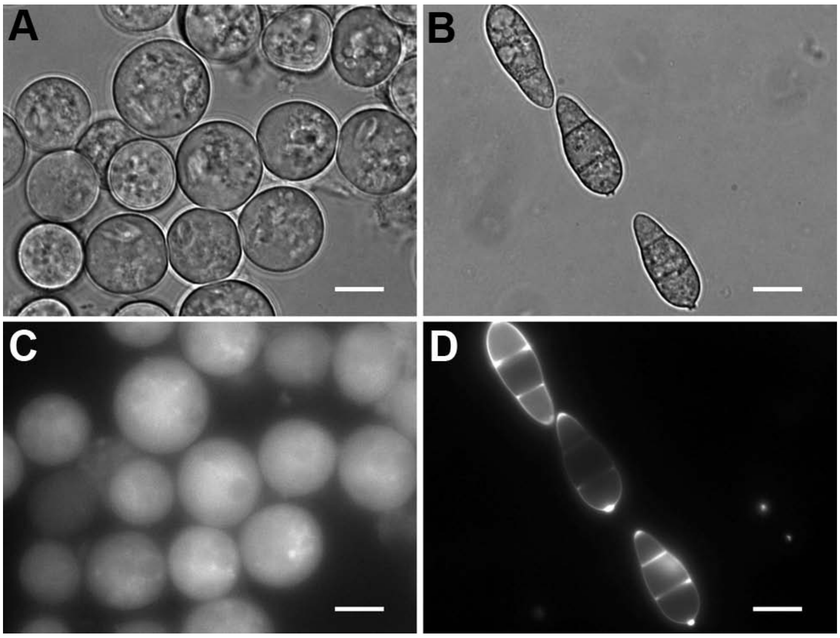

Supplementary Figure S10
