# Supplementary material for: Membrane Trafficking Modulation during Entamoeba Encystation
Source: Sci Rep. 2017 Oct 9;7:12854. doi: 10.1038/s41598-017-12875-6 (PMC5634486; doi:10.1038/s41598-017-12875-6)
Supplement: Supplementary file 5 — Table S4 [file 41598_2017_12875_MOESM5_ESM.doc]

Supplementary Table S4. Primers for Drp and control genes for RT-PCR and qPCR.

| **Primer Name** | **Primer sequence (5'-to-3')** | **Purpose** |
| --- | --- | --- |
|  |  |  |
| EiDrp1RTPCR_F | TACGGGAAAATTGCGGATAG | E. invadens specific RT-PCR primer |
| EiDrp1RTPCR_R | CCTCCAATCAGCTCGTGTTT | E. invadens specific RT-PCR primer |
| EiDrp2RTPCR_F | GACGAAATTAAACCGGACGA | E. invadens specific RT-PCR primer |
| EiDrp2RTPCR_R | AAACATGGTCGTGTCCCACT | E. invadens specific RT-PCR primer |
| EiDrp2aRTPCR_F | TCCGAATGTCGTTGATTTGA | E. invadens specific RT-PCR primer |
| EiDrp2aRTPCR_R | TCGAGTTGGTTCCTTTGTCC | E. invadens specific RT-PCR primer |
| EiDrp3RTPCR_F | GAGCCTTTCGACCAGTCTTG | E. invadens specific RT-PCR primer |
| EiDrp3RTPCR_R | TATTTCGCGGCTCAGGTACT | E. invadens specific RT-PCR primer |
| EiDrp4RTPCR_F | AATTATCGAGGCGATTGTCG | E. invadens specific RT-PCR primer |
| EiDrp4RTPCR_R | GTCGATCAAACACGATGGAA | E. invadens specific RT-PCR primer |
| EiActinRTPCR_F | CGAGCTGTCTTCCCATCAAT | E. invadens specific RT-PCR primer |
| EiActinRTPCR_R | TTAGCCTTTGGGTTCATTGG | E. invadens specific RT-PCR primer |
| qPCR primer |  |  |
| EiDrp3q_F | CAGAGCGACGGCAAAAGT | E. invadens quantitative PCR primer |
| EiDrp3q_R | GCAGAATCAACGGACGAC | E. invadens quantitative PCR primer |
| EiDrp4q_F | CGAAAGCAGCACAAGCAC | E. invadens quantitative PCR primer |
| EiDrp4q_R | TCCATCGCACCAATAGCA | E. invadens quantitative PCR primer |
| EiSKIPdomain_F | TGCCAGAACAACCCACAA | E. invadens quantitative PCR primer |
| EiSKIPdomain_R | TCAAACACGGGAGAACCAA | E. invadens quantitative PCR primer |
