# Supplementary material for: Membrane Trafficking Modulation during Entamoeba Encystation
Source: Sci Rep. 2017 Oct 9;7:12854. doi: 10.1038/s41598-017-12875-6 (PMC5634486; doi:10.1038/s41598-017-12875-6)
Supplement: Supplementary file 7 — Table S6 [file 41598_2017_12875_MOESM7_ESM.doc]

Supplementary Table S6. Membrane trafficking phylogeny metadata

GADEZ adaptin tree

| **Name** | **Species** | **Accession** | **Tree Round** | **Tree name** | **Orthology** |
| --- | --- | --- | --- | --- | --- |
| AP1G | H. sapiens | NP_001119.3 | R1 | HsAP1G | AP1G |
| AP2A | H. sapiens | NP_036437.1 | R1 | HsAP2A | AP2A |
| AP3D | H. sapiens | NP_003929.4 | R1 | HsAP3D | AP3D |
| AP4E | H. sapiens | NP_031373.2 | R1 | HsAP4E | AP4E |
| AP5Z | H. sapiens | NP_055670.1 | R1 | HsAP5Z | AP5Z |
| AP1G | S. cerevisiae | NP_015354.1 | R1 | ScAP1G | AP1G |
| AP2A | S. cerevisiae | NP_009516.1 | R1 | ScAP2A | AP2A |
| AP3D | S. cerevisiae | NP_015129.1 | R1 | ScAP3D | AP3D |
| AP1G | E. invadens | EIN_177790 | R1 | EiAP1G1 | AP1G |
| AP1G | E. invadens | EIN_388420 | R1 | EiAP1G2 | AP1G |
| AP2A | E. invadens | EIN_309570 | R1 | EiAP2A1 | AP2A |
| AP2A | E. invadens | EIN_079610 | R1 | EiAP2A2 | AP2A |
| AP3D | E. invadens | EIN_378020 | R1 | EiAP3D | AP3D |
| AP5Z | E. invadens | EIN_059810 | R1 | EiAP5Z | AP5Z |
| Unknown | E. invadens | EIN_406340 | R1 | EiUnkn | AP4E |
| AP1G | E. histolytica | EHI_118480 | R1 | EhAP1G1 | AP1G |
| AP1G | E. histolytica | EHI_196890 | R1 | EhAP1G2 | AP1G |
| AP2A | E. histolytica | EHI_153240 | R1 | EhAP2A1 | AP2A |
| AP2A | E. histolytica | EHI_045710 | R1 | EhAP2A2 | AP2A |
| AP2A | E. histolytica | EHI_011780 | R1 | EhAP2A3 | AP2A |
| AP2A | E. histolytica | EHI_014390 | R1 | EhAP2A4 | AP2A |
| AP3D | E. histolytica | EHI_164810 | R1 | EhAP3D | AP3D |
| AP5Z | E. histolytica | EHI_178870 | R1 | EhAP5Z | AP5Z |
| Unknown | E. histolytica | EHI_049590 | R1 | EhUnkn | AP4E |

Alignment metadata

24 taxa, 423 informative positions

Best model according to AIC: LG+G+F

α: 3.266

ArfGAP tree

| **Name** | **Species** | **Accession** | **Tree round** | **Tree name** | **Orthology** | **Ununsual domain structure** |
| --- | --- | --- | --- | --- | --- | --- |
| ACAP | H. sapiens | NP_055531 | R2 | HsACAP1 | ACAP |  |
| ACAP | H. sapiens | NP_036419 | R2 | HsACAP2 | ACAP |  |
| ACAP | H. sapiens | NP_085152 | R2 | HsACAP3 | ACAP |  |
| ADAP | H. sapiens | NP_006860 | R1 | HsADAP1 | ADAP |  |
| ADAP | H. sapiens | NP_060874 | R1 | HsADAP2 | ADAP |  |
| AGAP | H. sapiens | NP_055729 | R2 | HsAGAP1 | AGAP |  |
| AGAP | H. sapiens | NP_114152 | R2 | HsAGAP3 | AGAP |  |
| AGAP | H. sapiens | NP_597703 | R2 | HsAGAP4 | AGAP |  |
| AGFG | H. sapiens | NP_004495 | R2 | HsAGFG1 | AGFG |  |
| AGFG | H. sapiens | NP_006067 | R2 | HsAGFG2 | AGFG |  |
| ArfGAP1 | H. sapiens | NP_060679 | R2 | HsAGP1 | ArfGAP1 |  |
| ArfGAP2 | H. sapiens | NP_115765 | R2 | HsAGP2 | ArfGAP2 |  |
| ASAP | H. sapiens | NP_060952 | R1 | HsASAP1 | ASAP |  |
| ASAP | H. sapiens | NP_003878 | R1 | HsASAP2 | ASAP |  |
| ASAP | H. sapiens | NP_060177 | R1 | HsASAP3 | ASAP |  |
| SMAP | H. sapiens | NP_068759 | R2 | HsSMAP1 | SMAP |  |
| SMAP | H. sapiens | NP_073570 | R2 | HsSMAP2 | SMAP |  |
| ACAP | S. cerevisiae | NP_010812 | R2 | ScACAP | ACAP |  |
| ArfGAP1 | S. cerevisiae | NP_010055 | R2 | ScAGP1a | ArfGAP1 |  |
| ArfGAP1 | S. cerevisiae | NP_014195 | R2 | ScAGP1b | ArfGAP1 |  |
| ArfGAP2 | S. cerevisiae | NP_011048 | R2 | ScAGP2 | ArfGAP2 |  |
| SMAP | S. cerevisiae | NP_011334 | R2 | ScSMAP | SMAP |  |
| AGFG | E. invadens | EIN_291320 | R2 | EiAGFG | AGFG |  |
| ACAP | E. invadens | EIN_033800 | R2 | EiACAP | ACAP |  |
| AGAP | E. invadens | EIN_175880 | R2 | EiAGAP | ACAP |  |
| ArfGAP1 | E. invadens | EIN_409850 | R2 | EiAGP1a | ArfGAP1/2 |  |
| ArfGAP1 | E. invadens | EIN_155290 | R2 | EiAGP1b | ArfGAP1/2 |  |
| ArfGAP1 | E. invadens | EIN_155620 | R2 | EiAGP1c | ArfGAP1/2 |  |
| SMAP | E. invadens | EIN_032330, EIN_032440 | R2 | EiSMAP1 | ArfGAP1/2 |  |
| Unknown | E. invadens | EIN_064750 | R2 | EiUnkn1 | Uncharacterised | calponin, RhoGEF, pH, ArfGAP |
| SMAP | E. invadens | EIN_296020 | R2 | EiSMAP2 | ArfGAP1/2 |  |
| Unknown | E. invadens | EIN_252870 | R2 | EiUnkn2 | Uncharacterised | calponin, RhoGEF, pH, ArfGAP |
| Unknown | E. invadens | EIN_525070 | R2 | EiUnkn3 | Uncharacterised | calponin, RhoGEF, pH, ArfGAP |
| SMAP | E. invadens | EIN_250770 | R2 | EiSMAP3 | ArfGAP1/2 |  |
| Unknown | E. invadens | EIN_059750 | R2 | EiUnkn4 | Uncharacterised | calponin, RhoGEF, pH, ArfGAP |
| Unknown | E. invadens | EIN_495550 | R2 | EiUnkn5 | Uncharacterised | calponin, RhoGEF, pH, ArfGAP |
| SMAP | E. invadens | EIN_151890 | R2 | EiSMAP4 | Unknown |  |
| AGFG | E. histolytica | EHI_095050 | R2 | EhAGFG | AGFG |  |
| ACAP | E. histolytica | EHI_138360 | R2 | EhACAP1 | ACAP |  |
| ACAP | E. histolytica | EHI_152090 | R2 | EhACAP2 | ACAP |  |
| AGAP | E. histolytica | EHI_045400 | R2 | EhAGAP | ACAP |  |
| ArfGAP1 | E. histolytica | EHI_004810 | R2 | EhAGP1a | ArfGAP1/2 |  |
| ArfGAP1 | E. histolytica | EHI_069440 | R2 | EhAGP1b | ArfGAP1/2 |  |
| SMAP | E. histolytica | EHI_140140 | R2 | EhSMAP1 | SMAP |  |
| Unknown | E. histolytica | EHI_051820 | R2 | EhUnkn1 | Uncharacterised | calponin, RhoGEF, pH, ArfGAP |
| SMAP | E. histolytica | EHI_189140 | R2 | EhSMAP2 | ArfGAP1/2 |  |
| Unknown | E. histolytica | EHI_145730 | R2 | EhUnkn2 | Uncharacterised | calponin, RhoGEF, pH, ArfGAP |
| Unknown | E. histolytica | EHI_035770 | R2 | EhUnkn3 | Uncharacterised | calponin, RhoGEF, pH, ArfGAP |
| Unknown | E. histolytica | EHI_137680 | R2 | EhUnkn4 | Uncharacterised | calponin, RhoGEF, pH, ArfGAP |
| Unknown | E. histolytica | EHI_194630 | R2 | EhUnkn5 | Uncharacterised | calponin, RhoGEF, pH, ArfGAP |
| SMAP | E. histolytica | EHI_012490 | R2 | EhSMAP3 | ArfGAP1/2 |  |

Alignment metadata

46 taxa, 91 informative positions

Best model according to AIC: WAG+G+F

α: 3.266

Snf7 family tree

| **Name** | **Species** | **Accession** | **Tree Round** | **Tree name** | **Orthology** |
| --- | --- | --- | --- | --- | --- |
| Vps20 | H. sapiens | NP_078867 | R1 | HsVps20 | Vps20 |
| Vps32A | H. sapiens | NP_054888 | R1 | HsVps32A | Vps32A |
| Vps32B | H. sapiens | NP_789782 | R1 | HsVps32B | Vps32B |
| Vps32C | H. sapiens | NP_689497 | R1 | HsVps32C | Vps32C |
| Vps60 | H. sapiens | NP_057494 | R1 | HsVps60 | Vps60 |
| Vps20 | S. cerevisiae | NP_013794 | R1 | ScVps20 | Vps20 |
| Vps32 | S. cerevisiae | NP_013125 | R1 | ScVps32 | Vps32 |
| Vps60 | S. cerevisiae | NP_010774 | R1 | ScVps60 | Vps60 |
| Vps32 | E. invadens | EIN_250190 | R1 | EiVps32 | Vps32 |
| Vps60 | E. invadens | EIN_403050 | R1 | EiVps60 | Vps60 |
| Vps32 | E. histolytica | EHI_169820 | R1 | EhVps32 | Vps32 |
| Vps60 | E. histolytica | EHI_114790 | R1 | EhVps60 | Vps60 |
| Unknown | E. histolytica | EHI_048690 | R1 | EhUnkn | Vps32 |

Alignment metadata

13 taxa, 172 informative positions

Best model according to AIC: LG+G+F

α: 2.341

Qc family tree

| **Gene** | **Species** | **Accession** | **Tree Round** | **Tree Name** | **Orthology** |
| --- | --- | --- | --- | --- | --- |
| Syn8 | H. sapiens | NP_004844 | R2 | HsSyn8 | Syn8 |
| Syn6 | H. sapiens | NP_005810 | R2 | HsSyn6 | Syn6 |
| Syn10 | H. sapiens | NP_003756 | R2 | HsSyn10 | Syn10 |
| Bet1 | H. sapiens | NP_005859 | R2 | HsBet1 | Bet1 |
| Use1 | H. sapiens | NP_060937 | R2 | HsUse1 | Use1 |
| Syp7 | A. thaliana | NP_566354 | R2 | AtSyp7 | Syp7 |
| Syp7 | D. discoideum | XP_636414 | R2 | DdSyp7 | Syp7 |
| Syn8 | A. castellanii | XP_004336630 | R2 | AcSyn8 | Syn8 |
| Syn6/10 | A. castellanii | XP_004336724 | R2 | AcSyn610 | Syn6/10 |
| Syp7 | E. invadens | EIN_097080 | R2 | EiSyp7 | Syp7 |
| Bet1 | E. invadens | EIN_335210 | R2 only | EiBet1 | Bet1 |
| Unknown | E. invadens | EIN_253190 | R2 | EiUnkn1 | Syp7 |
| Unknown | E. invadens | EIN_084450 | R2 | EiUnkn2 | Syn6/10 |
| Unknown | E. invadens | EIN_056750 | R2 | EiUnkn3 | Syn6/10 |
| Syn6/10 | E. invadens | EIN_095140 | R2 only | EiSyn610 | Syn6/10 |
| Syp7 | E. histolytica | EHI_122770 | R2 | EhSyp7 | Syp7 |
| Bet1 | E. histolytica | EHI_086200 | R2 only | EhBet1 | Bet1 |
| Unknown | E. histolytica | EHI_107040 | R2 | EhUnkn1 | Syp7 |
| Unknown | E. histolytica | EHI_106240 | R2 | EhUnkn2 | Syn6/10 |
| Unknown | E. histolytica | EHI_155680 | R2 | EhUnkn3 | Syn6/10 |
| Syn6/10 | E. histolytica | EHI_025340 | R2 only | EhSyn610 | Syn6/10 |

Alignment metadata

21 taxa, 104 informative positions

Best model according to AIC: LG+G

α: 3.671

TBC tree

| Gene | Species | Accession | Tree Name | Tree Round | Orthology (BAYES) |
| --- | --- | --- | --- | --- | --- |
| A | Homo sapiens | NP_060222.2 | HS_A | R2 | A |
| B | Homo sapiens | NP_061893.2 | HS_B | R2 | B |
| B | Arabidopsis thaliana | NP_181877.2 | AT_B | R2 | B |
| B | E. invadens | EIN_312800 | EI_B1 | R2 | B |
| B | E. invadens | EIN_408100, EIN_408210 | EI_B2 | R2 | B |
| B | E. invadens | EIN_424290 | EI_B3 | R2 | B |
| B | E. invadens | EIN_468110 | EI_B4 | R2 | B |
| B | E. invadens | EIN_391440 | EI_B5 | R2 | B |
| B | E. invadens | EIN_173500 | EI_B6 | R2 | B |
| B | E. invadens | EIN_084610 | EI_B7 | R2 | B |
| B | E. invadens | EIN_181560 | EI_B8 | R2 | B |
| B | E. invadens | EIN_153140 | EI_B9 | R2 | B |
| B | E. invadens | EIN_523780 | EI_B10 | R2 | B |
| B | E. invadens | EIN_409980 | EI_B11 | R2 | B |
| B | E. invadens | EIN_066090 | EI_B12 | R2 | Unknown |
| B | E. invadens | EIN_023440 | EI_B13 | R2 | B |
| B | E. invadens | EIN_054060, EIN_054170 | EI_B14 | R2 | B |
| B | E. histolytica | EHI_121850 | EH_B1 | R2 | B |
| B | E. histolytica | EHI_110550 | EH_B2 | R2 | B |
| B | E. histolytica | EHI_124440 | EH_B3 | R2 | B |
| B | E. histolytica | EHI_094140 | EH_B4 | R2 | B |
| B | E. histolytica | EHI_045210 | EH_B5 | R2 | B |
| B | E. histolytica | EHI_081670 | EH_B6 | R2 | B |
| B | E. histolytica | EHI_198700 | EH_B7 | R2 | Unknown |
| B | E. histolytica | EHI_035150 | EH_B8 | R2 | B |
| B | E. histolytica | EHI_052170 | EH_B9 | R2 | B |
| C | Homo sapiens | NP_699187 | HS_C | R1 | C |
| D | Homo sapiens | NP_055161.1 | HS_D | R2 | D |
| D | Arabidopsis thaliana | NP_565706.1 | AT_D | R2 | D |
| D | E. invadens | EIN_344790 | EI_D1 | R2 | D |
| D | E. invadens | EIN_185080 | EI_D2 | R2 | D |
| D | E. invadens | EIN_177730 | EI_D3 | R2 | D |
| D | E. invadens | EIN_058720 | EI_D4 | R2 | D |
| D | E. invadens | EIN_173180 | EI_D5 | R2 | D |
| D | E. invadens | EIN_059460 | EI_D6 | R2 | Unknown |
| D | E. invadens | EIN_151350 | EI_D7 | R2 | Unknown |
| D | E. invadens | EIN_229420 | EI_D8 | R2 | Unknown |
| D | E. histolytica | EHI_196990 | EH_D1 | R2 | D |
| D | E. histolytica | EHI_151210 | EH_D2 | R2 | D |
| D | E. histolytica | EHI_166020 | EH_D3 | R2 | D |
| D | E. histolytica | EHI_069260 | EH_D4 | R2 | Unknown |
| D | E. histolytica | EHI_064580 | EH_D5 | R2 | D |
| D | E. histolytica | EHI_035140 | EH_D6 | R2 | Unknown |
| D | E. histolytica | EHI_060420 | EH_D7 | R2 | Unknown |
| E | Homo sapiens | NP_666364.1 | HS_E | R2 | E |
| E | Arabidopsis thaliana | AT1G04830.2 | AT_E | R2 | E |
| E | E. invadens | EIN_250610 | EI_E1 | R2 | E |
| E | E. invadens | EIN_162190 | EI_E2 | R2 | E |
| E | E. invadens | EIN_151320 | EI_E3 | R2 | E |
| E | E. invadens | EIN_082980 | EI_E4 | R2 | E |
| E | E. invadens | EIN_062690 | EI_E5 | R2 | F |
| E | E. invadens | KB206244-3-61299-61688 (found on unplaced genomic scaffold) | EI_E6 | R2 | E |
| E | E. histolytica | EHI_200780 | EH_E1 | R2 | E |
| E | E. histolytica | EHI_142920 | EH_E2 | R2 | E |
| E | E. histolytica | EHI_187060 | EH_E3 | R2 | E |
| E | E. histolytica | EHI_040270 | EH_E4 | R2 | E |
| E | E. histolytica | EHI_152910 | EH_E5 | R2 | F |
| F | Homo sapiens | NP_055559.1 | HS_F | R2 | F |
| F | E. invadens | EIN_023590 | EI_F1 | R2 | F |
| F | E. invadens | EIN_066110 | EI_F2 | R2 | F |
| F | E. invadens | EIN_229360 | EI_F3 | R2 | F |
| F | E. invadens | EIN_096710 | EI_F4 | R2 | F |
| F | E. invadens | EIN_487780 | EI_F5 | R2 | F |
| F | E. invadens | EIN_018740 | EI_F6 | R2 | F |
| F | E. histolytica | EHI_169850 | EH_F1 | R2 | F |
| F | E. histolytica | EHI_148860 | EH_F2 | R2 | F |
| F | E. histolytica | EHI_006780 | EH_F3 | R2 | F |
| F | E. histolytica | EHI_170310 | EH_F4 | R2 | F |
| F | E. histolytica | EHI_068360 | EH_F5 | R2 | F |
| F | E. histolytica | EHI_009970 | EH_F6 | R2 | F |
| G | Homo sapiens | NP_149106.2 | HS_G | R2 | G |
| G | E. invadens | EIN_390780 | EI_G | R2 | G |
| G | E. histolytica | EHI_038840 | EH_G | R2 | G |
| H | Homo sapiens | NP_060787.2 | HS_H | R2 | H |
| I | Homo sapiens | NP_060779.2 | HS_I | R2 | I |
| I | E. invadens | EIN_176880 | EI_I1 | R2 | Unknown |
| I | E. invadens | EIN_080920 | EI_I2 | R2 | K |
| I | E. invadens | EIN_086640 | EI_I3 | R2 | Unknown |
| I | E. invadens | EIN_175590 | EI_I4 | R2 | Unknown |
| I | E. histolytica | EHI_126040 | EH_I1 | R2 | Unknown |
| I | E. histolytica | EHI_175400 | EH_I2 | R2 | K |
| I | E. histolytica | EHI_054250 | EH_I3 | R2 | Unknown |
| J | Homo sapiens | NP_057579.1 | HS_J | R1 | J |
| K | Homo sapiens | NP_065756.1 | HS_K | R2 | K |
| L | Homo sapiens | NP_663622.2 | HS_L | R2 | L |
| M | Homo sapiens | NP_653229.1 | HS_M | R2 | M |
| M | E. invadens | EIN_152810 | EI_M | R2 | M |
| M | E. histolytica | EHI_015260 | EH_M | R2 | M |
| N | Homo sapiens | NP_056003.1 | HS_N | R2 | N |
| O | Homo sapiens | NP_055503.1 | HS_O | R2 | O |
| O | E. invadens | EIN_182950 | EI_O1 | R2 | O |
| O | E. invadens | EIN_283550 | EI_O2 | R2 | O |
| O | E. invadens | EIN_487650 | EI_O3 | R2 | O |
| O | E. invadens | EIN_380040 | EI_O4 | R2 | O |
| O | E. invadens | EIN_186660 | EI_O5 | R2 | O |
| O | E. invadens | EIN_184560 | EI_O6 | R2 | O |
| O | E. invadens | EIN_207540 | EI_O7 | R2 | O |
| O | E. histolytica | EHI_010690 | EH_O1 | R2 | O |
| O | E. histolytica | EHI_009910 | EH_O2 | R2 | O |
| O | E. histolytica | EHI_049830 | EH_O3 | R2 | O |
| O | E. histolytica | EHI_165290 | EH_O4 | R2 | O |
| O | E. histolytica | EHI_149900 | EH_O5 | R2 | O |
| P | Homo sapiens | NP_056342.3 | HS_P | R2 | P |
| Q | Homo sapiens | NP_055647.2 | HS_Q | R2 | Q |
| R | Homo sapiens | NP_078995.2 | HS_R | R1 | R |
| S | Homo sapiens | NP_056520.2 | HS_S | R1 | S |
| V | Homo sapiens | NP_056094.1 | HS_V | R1 | V |
| X | Homo sapiens | NP_653173.1 | HS_X | R1 | X |
| T | Saccharomyces cerevisiae | NP_013771.1 | SC_T | R2 | T |
| W | Saccharomyces cerevisiae | NP_012491.3 | SC_W | R1 | W |
| RootA | Trypanosoma brucei | Tb10.6k15.2930 | TB_Root | R2 | RootA |
| RootA | E. invadens | EIN_411080 | EI_ROOT | R2 | Root |
| RootA | E. histolytica | EHI_009510 | EH_ROOT | R2 | Root |
| PlA | Arabidopsis thaliana | NP_566323.1 | AT_PlA | R1 | PlA |
| PlB | Arabidopsis thaliana | NP_181460.3 | AT_PlB | R1 | PlB |
| ExA | Naegleria gruberi | XP_002677809.1 | NG_ExA | R1 | ExA |
| Unknown | E. invadens | EIN_047680 | EI_U1 | R2 | Potential Q |
| Unknown | E. invadens | EIN_172080 | EI_U2 | R2 | E |
| Unknown | E. invadens | EIN_308420 | EI_U3 | R2 | Potential A |
| Unknown | E. invadens | EIN_274660 | EI_U4 | R2 | T |
| Unknown | E. invadens | EIN_462240 | EI_U5 | R2 | M |
| Unknown | E. invadens | EIN_060150 | EI_U6 | R2 | D |
| Unknown | E. invadens | EIN_182960 | EI_U7 | R2 | Unknown |
| Unknown | E. invadens | EIN_129360 | EI_U8 | R2 | Unknown |
| Unknown | E. invadens | EIN_175830 | EI_U9 | R2 | Unknown |
| Unknown | E. histolytica | EHI_010540 | EH_U1 | R2 | Potential Q |
| Unknown | E. histolytica | EHI_091080 | EH_U2 | R2 | E |
| Unknown | E. histolytica | EHI_189120 | EH_U3 | R2 | Potential A |
| Unknown | E. histolytica | EHI_135170 | EH_U4 | R2 | T |
| Unknown | E. histolytica | EHI_038630 | EH_U5 | R2 | Unknown |
| Unknown | E. histolytica | EHI_152630 | EH_U6 | R2 | D |
| Unknown | E. histolytica | EHI_181530 | EH_U7 | R2 | K |
| Unknown | E. histolytica | EHI_177300 | EH_U8 | R2 | Unknown |
| Unknown | E. histolytica | EHI_182050 | EH_U9 | R2 | Unknown |

Alignment metadata

134 taxa, 165 informative positions

Best model according to AIC: LG+G+F

α: 2.160

NB: the maximum discrepancy across bipartitions reached 0.19, which is considered by the makers of Phylobayes to be "acceptable: gives a good qualitative picture of the posterior consensus." The effective sample sizes exceeded 100 with the exception of nocc and allocent statistics.

ArfGEF tree

| **Gene** | **Species** | **Accession** | **Tree Name** | **Orthology** | **Domain structure of Entamoeba sequences** |
| --- | --- | --- | --- | --- | --- |
| GBF1 | Homo sapiens | NP_004184.1 | HsGBF1 | GBF1 |  |
| BIG1 | Homo sapiens | NP_006412.2 | HsBIG1a | BIG1 |  |
| BIG1 | Homo sapiens | NP_006411.2 | HsBIG1b | BIG1 |  |
| Cytohesin | Homo sapiens | NP_004753.1 | HsCyt1 | Cytohesin |  |
| Cytohesin | Homo sapiens | NP_059431.1 | HsCyt2 | Cytohesin |  |
| Cytohesin | Homo sapiens | NP_004218.1 | HsCyt3 | Cytohesin |  |
| Cytohesin | Homo sapiens | NP_037517.1 | HsCyt4 | Cytohesin |  |
| EFA6 | Homo sapiens | NP_002770.3 | HsEFA6a | EFA6 |  |
| EFA6 | Homo sapiens | NP_036587.2 | HsEFA6b | EFA6 |  |
| EFA6 | Homo sapiens | NP_115665.1 | HsEFA6c | EFA6 |  |
| EFA6 | Homo sapiens | NP_996792.1 | HsEFA6d | EFA6 |  |
| BRAG1 | Homo sapiens | NP_055890.1 | HsBRAG1 | BRAG1 |  |
| BRAG2 | Homo sapiens | NP_055684.3 | HsBRAG2 | BRAG2 |  |
| BRAG3 | Homo sapiens | NP_056047.1 | HsBRAG3 | BRAG3 |  |
| FBX8 | Homo sapiens | NP_036312.2 | HsFBX8 | FBX8 |  |
| BIG1 | E. invadens | EIN_224400 | EiBIG1a | BIG/GBF-like | Sec7 |
| BIG1 | E. invadens | EIN_467870 | EiBIG1b | BIG/GBF-like | Sec7 |
| BIG1 | E. invadens | EIN_167960 | EiBIG1c | BIG/GBF-like | Sec7 |
| BIG1 | E. invadens | EIN_085970 | EiBIG1d | BIG/GBF-like | Sec7 |
| BIG1 | E. invadens | EIN_407440 | EiBIG1e | BIG/GBF-like | Sec7 |
| Unknown | E. invadens | EIN_046880 | EiUnkn | BIG/GBF-like | Sec7 |
| Cytohesin | E. invadens | EIN_165270 | EiCyt1 | BIG/GBF-like | Sec7 |
| Cytohesin | E. invadens | EIN_274500 | EiCyt2 | cytohesin-like | PH |
| Cytohesin | E. invadens | EIN_168830 | EiCyt3 | cytohesin-like | PH |
| Cytohesin | E. invadens | EIN_168630 | EiCyt4 | cytohesin-like | PH |
| Cytohesin | E. invadens | EIN_430410 | EiCyt5 | cytohesin-like | PH |
| Cytohesin | E. invadens | EIN_054410 | EiCyt6 | BIG/GBF-like | Sec7 |
| Cytohesin | E. invadens | EIN_416730 | EiCyt7 | BIG/GBF-like | Sec7 |
| BIG1 | E. histolytica | XP_652288 | EhBIG1a | BIG/GBF-like | Sec7 |
| BIG1 | E. histolytica | XP_655381 | EhBIG1b | BIG/GBF-like | Sec7 |
| BIG1 | E. histolytica | XP_652701 | EhBIG1c | BIG/GBF-like | Sec7 |
| BIG1 | E. histolytica | XP_651947 | EhBIG1d | BIG/GBF-like | Sec7 |
| Unknown | E. histolytica | XP_656257 | EhUnkn | BIG/GBF-like | Sec7 |
| Cytohesin | E. histolytica | XP_653354 | EhCyt1 | BIG/GBF-like | Sec7 |
| Cytohesin | E. histolytica | XP_656064 | EhCyt2 | cytohesin-like | PH |
| Cytohesin | E. histolytica | XP_654091 | EhCyt3 | cytohesin-like | PH |
| Cytohesin | E. histolytica | XP_655295 | EhCyt4 | cytohesin-like | PH |
| Cytohesin | E. histolytica | XP_653366 | EhCyt5 | cytohesin-like | PH |
| Cytohesin | E. histolytica | XP_656882 | EhCyt6 | BIG/GBF-like | Sec7 |
| Cytohesin | E. histolytica | XP_654240 | EhCyt7 | BIG/GBF-like | Sec7 |
| BIG1 | S. cerevisiae | NP_010892 | ScBIG1 | BIG1 |  |
| Sec7 | S. cerevisiae | NP_010454 | ScSec7 | Sec7 |  |

Alignment metadata

42 taxa, 132 informative positions

Best model according to AIC: LG+G

α: 0.733

DENN trees

| Gene | Species | Accession | Tree name |
| --- | --- | --- | --- |
| DENN1 | Homo sapiens | NP_065997 | HS_D1 |
| DENN2 | Homo sapiens | NP_056504.3 | HS_D2 |
| DENN3 | Homo sapiens | NP_055772.2 | HS_D3 |
| DENN4 | Homo sapiens | NP_005839.3 | HS_D4 |
| DENN5 | Homo sapiens | NP_056028.2 | HS_D5 |
| SBF1 | Homo sapiens | NP_002963.2 | HS_SBF1 |
| SBF2 | Homo sapiens | NP_112224.1 | HS_SBF2 |
| MADD | Homo sapiens | NP_569826.2 | HS_MADD |
| c9ORF72 | Homo sapiens | NP_060795.1 | HS_orf |
| FNIP1 | Homo sapiens | NP_588613.2 | HS_FNIP1 |
| FNIP2 | Homo sapiens | NP_065891.1 | HS_FNIP2 |
| FLCN | Homo sapiens | NP_659434.2 | HS_FLCN |
| SMCR8 | Homo sapiens | NP_658988.2 | HS_SMCR8 |
| DENN1 | E. invadens | EIN_247110 | EI_D1 |
| DENN2 | E. invadens | EIN_229350 | EI_D2a |
| DENN2 | E. invadens | EIN_065240 | EI_D2b |
| DENN2 | E. invadens | EIN_132330 | EI_D2c |
| DENN2 | E. invadens | EIN_409110 | EI_D2d |
| DENN2 | E. invadens | EIN_226480 | EI_D2e |
| DENN2 | E. invadens | EIN_033640 | EI_D2f |
| DENN2 | E. invadens | EIN_312910 | EI_D2g |
| DENN5 | E. invadens | EIN_057210 | EI_D5a |
| DENN5 | E. invadens | EIN_344580 | EI_D5b |
| Unknown | E. invadens | EIN_425080 | EI_Un1 |
| Unknown | E. invadens | EIN_379960 | EI_Un2 |
| Unknown | E. invadens | EIN_397640 | EI_Un3 |
| Unknown | E. invadens | EIN_054470 | EI_Un4 |
| Unknown | E. invadens | EIN_359150 | EI_Un5 |
| Unknown | E. invadens | EIN_406200 | EI_Un6 |
| Unknown | E. invadens | EIN_369370 | EI_Un7 |
| Unknown | E. invadens | EIN_155240 | EI_Un8 |
| Unknown | E. invadens | EIN_085070 | EI_Un9 |
| Unknown | E. invadens | EIN_284020 | EI_Un10 |
| Unknown | E. invadens | EIN_310000 | EI_Un11 |
| Unknown | E. invadens | EIN_084150 | EI_Un12 |
| Unknown | E. invadens | EIN_284770 | EI_Un13 |
| Unknown | E. invadens | EIN_410170 | EI_Un14 |
| Unknown | E. invadens | EIN_369580 | EI_Un15 |
| Unknown | E. invadens | EIN_054610 | EI_Un16 |
| Unknown | E. invadens | EIN_397630 | EI_Un17 |
| DENN1 | E. histolytica | XP_655410 | EH_D1 |
| DENN2 | E. histolytica | XP_650065 | EH_D2a |
| DENN2 | E. histolytica | XP_655631 | EH_D2b |
| DENN2 | E. histolytica | XP_651655 | EH_D2c |
| DENN2 | E. histolytica | XP_654679 | EH_D2d |
| DENN5 | E. histolytica | XP_649481 | EH_D5a |
| DENN5 | E. histolytica | XP_654342 | EH_D5b |
| Unknown | E. histolytica | XP_647978 | EH_Un1 |
| Unknown | E. histolytica | XP_001913916 | EH_Un2 |
| Unknown | E. histolytica | XP_650798 | EH_Un3 |
| Unknown | E. histolytica | XP_648702 | EH_Un4 |
| Unknown | E. histolytica | XP_656256 | EH_Un5 |
| Unknown | E. histolytica | XP_654833 | EH_Un6 |
| Unknown | E. histolytica | XP_653340 | EH_Un7 |
| Unknown | E. histolytica | XP_655112 | EH_Un8 |
| Unknown | E. histolytica | XP_654827 | EH_Un9 |
| Unknown | E. histolytica | XP_656962 | EH_Un10 |
| Unknown | E. histolytica | XP_651359 | EH_Un11 |
| Unknown | E. histolytica | XP_652794 | EH_Un12 |
| Unknown | E. histolytica | XP_651679 | EH_Un13 |
| Unknown | E. histolytica | XP_656473 | EH_Un14 |
| Unknown | E. histolytica | XP_650334 | EH_Un15 |
| Unknown | E. histolytica | XP_655061 | EH_Un16 |

Alignment metadata

63 taxa, 196 informative positions

Best model according to AIC: LG+G+F

α: 2.680

Arf Orthology Tree (Supplementary Figure S7)

See Supplementary Figure S2A and S2B - taxon labels include species and NCBI accession

Alignment metadata

25 taxa, 156 informative positions

Best model according to AIC: LG+G+F

α: 1.806

NB: The MrBAYES tree had an average standard deviation of splits frequencies of 0.011, slightly higher than our 0.01 cutoff.

Rab Orthology Tree (Supplementary File 3 and Supplementary File 4)

| Classification in Nakada-Tsukui et al. 2010 | Species | Tree name | AmoebaDB accession | Orthology group based on MrBAYES node support > 0.8 posterior probability |
| --- | --- | --- | --- | --- |
| Rab1 | E. invadens | EinRab1A | EIN_171560 | Rab1 |
| Rab1 | E. invadens | EinRab1B | EIN_051580 | Rab1 |
| Rab1 | E. histolytica | EhiRab1A | EHI_108610 | Rab1 |
| Rab1 | E. histolytica | EhiRab1B | EHI_146510 | Rab1 |
| Rab2 | E. invadens | EinRab2A | EIN_340720 | Rab2 |
| Rab2 | E. invadens | EinRab2B | EIN_470040 | Rab2 |
| Rab2 | E. histolytica | EhiRab2A | EHI_146320 | Rab2 |
| Rab2 | E. histolytica | EhiRab2B | EHI_046390 | Rab2 |
| Rab2 | E. histolytica | EhiRab2C | EHI_067850 | Rab2 |
| Rab5 | E. invadens | EinRab5A | EIN_467890 | Rab5 |
| Rab5 | E. invadens | EinRab5B | EIN_081810 | Rab5 |
| Rab5 | E. histolytica | EhiRab5 | EHI_025420 | Rab5 |
| Rab7 | E. invadens | EinRab7A | EIN_186960 | Rab7 |
| Rab7 | E. invadens | EinRab7B | EIN_344190 | Rab7 |
| Rab7 | E. invadens | EinRab7C | EIN_154400 | Rab7 |
| Rab7 | E. invadens | EinRab7D | EIN_222060 | Rab7 |
| Rab7 | E. invadens | EinRab7E | EIN_253180 | Rab7 |
| Rab7 | E. invadens | EinRab7F | EIN_397580 | Rab7 |
| Rab7 | E. invadens | EinRab7G1 | EIN_020760 | Rab7 |
| Rab7 | E. invadens | EinRab7G2 | EIN_503260 | Rab7 |
| Rab7 | E. invadens | EinRab7H | EIN_485870 | Rab7 |
| Rab7 | E. invadens | EinRab7I | EIN_335590 | Rab7 |
| Rab7 | E. histolytica | EhiRab7A | EHI_192810 | Rab7 |
| Rab7 | E. histolytica | EhiRab7B | EHI_081330 | Rab7 |
| Rab7 | E. histolytica | EhiRab7C | EHI_189990 | Rab7 |
| Rab7 | E. histolytica | EhiRab7D | EHI_082070 | Rab7 |
| Rab7 | E. histolytica | EhiRab7E | EHI_169280 | Rab7 |
| Rab7 | E. histolytica | EhiRab7F | EHI_192130 | Rab7 |
| Rab7 | E. histolytica | EhiRab7G | EHI_187090 | Rab7 |
| Rab7 | E. histolytica | EhiRab7H | EHI_005900 | Rab7 |
| Rab7 | E. histolytica | EhiRab7I | EHI_189100 | Rab7 |
| RAB8 | E. invadens | EinRab8A | EIN_424250 | RAB8 |
| RAB8 | E. invadens | EinRab8B | EIN_253330 | RAB8 |
| RAB8 | E. histolytica | EhiRab8A | EHI_199820 | RAB8 |
| RAB8 | E. histolytica | EhiRab8B | EHI_127380 | RAB8 |
| RAB11 | E. invadens | EinRab11A | EIN_281310 | RAB11 |
| RAB11 | E. invadens | EinRab11B | EIN_177590 | RAB11 |
| RAB11 | E. invadens | EinRab11C | EIN_018350 | RAB11 |
| RAB11 | E. invadens | EinRab11D | EIN_080290 | RAB11 |
| RAB11 | E. histolytica | EhiRab11A | EHI_005460 | RAB11 |
| RAB11 | E. histolytica | EhiRab11B | EHI_107250 | RAB11 |
| RAB11 | E. histolytica | EhiRab11C | EHI_161030 | RAB11 |
| RAB11 | E. histolytica | EhiRab11D | EHI_056100 | RAB11 |
| RAB21 | E. histolytica | EhiRab21 | EHI_129330 | RAB21 |
| RABX31 | E. histolytica | EhiRabX31 | EHI_040310 | RAB21 |
| RABX31A | E. invadens | EinRabX31A | EIN_212140 | RAB21 |
| RABX31B | E. invadens | EinRabX31B | EIN_442200 | RAB21 |
| RABX31C | E. invadens | EinRabX31C | EIN_327220 | RAB21 |
| RABX31D | E. invadens | EinRabX31D | EIN_337120 | RAB21 |
| RABZ13 | E. invadens | EinRabZ13 | EIN_025950 | RAB21 |
| RABL1 | E. invadens | EinRabL1 | EIN_131900 | Rab32 |
| RABL2 | E. invadens | EinRabL2 | EIN_098580 | Rab32 |
| RabL1 | E. histolytica | EhiRabL1 | EHI_169090 | Rab32 |
| RabL2 | E. histolytica | EhiRab32a | EHI_069500 | Rab32 |
| RabX11 | E. histolytica | EhiRabX11 | EHI_177520 | Rab32 |
| RabX11 | E. invadens | EinRabX11A | EIN_020100 | Rab32 |
| RabX11 | E. invadens | EinRabX11B | EIN_096000 | Rab32 |
| RabX11 | E. invadens | EinRabX11C | EIN_150070 | Rab32 |
| RABA | E. invadens | EinRabA | EIN_474290 | RabA |
| RABA | E. histolytica | EhiRabA | EHI_168600 | RabA |
| RABH | E. invadens | EinRabH | EIN_267510 | RabA |
| RABH | E. histolytica | EhiRabH | EHI_133100 EHI_128180 | RabA |
| RABB | E. invadens | EinRabB | EIN_183080 | RabB |
| RABB | E. histolytica | EhiRabB | EHI_181240 | RabB |
| RABC | E. invadens | EinRabC1 | EIN_095580 | RabC1 |
| RABC | E. histolytica | EhiRabC1 | EHI_153690 | RabC1 |
| RABC | E. invadens | EinRabC3a | EIN_152450 | RabC1 |
| RABC | E. histolytica | EhiRabC3 | EHI_143650 | RabC1 |
| RABC | E. invadens | EinRabC3b | EIN_146120 | RabC1 |
| RABC | E. invadens | EinRabC4 | EIN_117950 | RabC1 |
| RABC | E. histolytica | EhiRabC4 | EHI_096220 | RabC1 |
| RABZ3 | E. invadens | EinRabZ3 | EIN_327740 | RabC1 |
| RABZ18 | E. invadens | EinRabZ18 | EIN_032570 | RabC1 |
| RABZ2A | E. invadens | EinRabZ2A | EIN_487230 | RabC1 |
| RABZ2B | E. invadens | EinRabZ2B | EIN_480990 | RabC1 |
| RABC | E. invadens | EinRabC2 | EIN_154550 | RabC2 |
| RABC | E. histolytica | EhiRabC2 | EHI_045550 | RabC2 |
| RABC | E. invadens | EinRabC5 | EIN_019940 | RabC5 |
| RABC | E. histolytica | EhiRabC5 | EHI_122730 | RabC5 |
| RABC | E. histolytica | EhiRabC6 | EHI_194280 | RabC6 |
| RABC | E. invadens | EinRabC7 | EIN_368780 | RabC6 |
| RABC | E. histolytica | EhiRabC7 | EHI_079890 | RabC6 |
| RABC | E. invadens | EinRabC8 | EIN_284900 | RabC6 |
| RABC | E. histolytica | EhiRabC8 | EHI_170390 | RabC6 |
| RabD | E. invadens | EinRabD | EIN_312790 | RabD |
| RabD | E. histolytica | EhiRabD1 | EHI_059670 | RabD |
| RabD | E. histolytica | EhiRabD2 | EHI_164900 | RabD |
| RabF | E. invadens | EinRabF1 | EIN_404670 | RabF1 |
| RabF | E. histolytica | EhiRabF2 | EHI_129740 | RabF1 |
| RabF | E. invadens | EinRabF5 | EIN_387130 | RabF1 |
| RabF | E. histolytica | EhiRabF5 | EHI_117960 | RabF1 |
| RabF | E. invadens | EinRabF2 | EIN_173940 | RabF2 |
| RabF | E. invadens | EinRabF3 | EIN_187370 | RabF2 |
| RabF | E. invadens | EinRabF4 | EIN_404600 | RabF2 |
| RabF | E. histolytica | EhiRabF2 | EHI_182030 | RabF2 |
| RabF | E. histolytica | EhiRabF3 | EHI_164880 | RabF2 |
| RabF | E. histolytica | EhiRabF4 | EHI_122870 | RabF2 |
| RabF | E. invadens | EinRabX9 | EIN_411160 | RabF2 |
| RabF | E. histolytica | EhiRabX9 | EHI_140770 | RabF2 |
| RabI | E. invadens | EinRabI | EIN_018410 | RabI |
| RabI | E. histolytica | EhiRabI1 | EHI_177550 | RabI |
| RabI | E. histolytica | EhiRabI2 | EHI_053420 | RabI |
| RabK | E. invadens | EinRabK1 | EIN_524790 | RabK |
| RabK | E. invadens | EinRabK2 | EIN_410790 | RabK |
| RabK | E. invadens | EinRabK4 | EIN_095980 | RabK |
| RabK | E. invadens | EinRabK5 | EIN_046430 | RabK |
| RabK | E. histolytica | EhiRabK1 | EHI_024680 | RabK |
| RabK | E. histolytica | EhiRabK2 | EHI_040450 | RabK |
| RabK | E. histolytica | EhiRabK3 | EHI_082550 | RabK |
| RabK | E. histolytica | EhiRabK4 | EHI_128110 | RabK |
| RabK | E. histolytica | EhiRabK5 | EHI_012380 | RabK |
| RabZ14 | E. invadens | EinRabZ14 | EIN_098590 | RabK |
| RabM | E. invadens | EinRabM1 | EIN_087180 | RabM |
| RabM | E. invadens | EinRabM3 | EIN_083840 | RabM |
| RabM | E. histolytica | EhiRabM1 | EHI_005010 | RabM |
| RabM | E. histolytica | EhiRabM2 | EHI_004380 | RabM |
| RabM | E. histolytica | EhiRabM3 | EHI_068230 | RabM |
| RabN | E. invadens | EinRabN1 | EIN_229080 | RabN |
| RabN | E. invadens | EinRabN2 | EIN_087490 | RabN |
| RabN | E. histolytica | EhiRabN1 | EHI_048250 | RabN |
| RabN | E. histolytica | EhiRabN2 | EHI_097650 | RabN |
| RabP | E. invadens | EinRabP | EIN_503120 | RabP |
| RabP | E. histolytica | EhiRabP1 | EHI_114210 | RabP |
| RabP | E. histolytica | EhiRabP2 | EHI_117890 | RabP |
| RabX30 | E. invadens | EinRabX30 | EIN_252610 | RabP |
| RabX30 | E. histolytica | EhiRabX30 | EHI_082230 | RabP |
| RabX18 | E. histolytica | EhiRabX18 | EHI_145720 | RabX18 |
| RabX39 | E. invadens | EinRabX39 | EIN_403070 | RabX39 |
| RabX39 | E. histolytica | EhiRabX39 | EHI_094110 | RabX39 |
| RabX15 | E. invadens | EinRabX15 | EIN_403340 | RabX39 |
| RabX15 | E. histolytica | EhiRabX15 | EHI_177390 | RabX39 |
| RabZ25 | E. invadens | EinRabZ25 | EIN_186350 | RabX39 |
| RabX1 | E. invadens | EinRabX1A | EIN_505160 | RabX1 |
| RabX1 | E. invadens | EinRabX1B | EIN_185820 | RabX1 |
| RabX1 | E. histolytica | EhiRabX1 | EHI_017740 | RabX1 |
| RabX3 | E. invadens | EinRabX3 | EIN_226630 | RabX3 |
| RABX4 | E. invadens | EinRabX4 | EIN_406050 | RabX4 |
| RABX4 | E. histolytica | EhiRabX4 | EHI_100090 | RabX4 |
| RAXX12 | E. histolytica | EhiRaxX12 | EHI_125980 | RabX4 |
| RabX5 | E. histolytica | EhiRabX5 | EHI_148660 | RabX5 |
| RabX6 | E. invadens | EinRabX6 | EIN_205430 | RabX6 |
| RabX6 | E. histolytica | EhiRabX6 | EHI_008350 | RabX6 |
| RabX7 | E. invadens | EinRabX7 | EIN_316560 | RabX6 |
| RabX7 | E. histolytica | EhiRabX7 | EHI_118920 | RabX6 |
| RabX29 | E. invadens | EinRabX29 | EIN_081940 | RabX6 |
| RabX29 | E. histolytica | EhiRabX29 | EHI_184670 | RabX6 |
| RabZ12 | E. invadens | EinRabZ12 | EIN_061290 | RabX6 |
| RabZ26 | E. invadens | EinRabZ26 | EIN_412340 | RabX6 |
| RabZ8 | E. invadens | EinRabZ8A | EIN_457360 | RabX6 |
| RabZ8 | E. invadens | EinRabZ8B | EIN_440750 | RabX6 |
| RabX41 | E. invadens | EinRabX41 | EIN_317000 | RabX6 |
| RabX8 | E. histolytica | EhiRabX8 | EHI_178040 | RabX8 |
| RabX10 | E. invadens | EinRabX10 | EIN_274260 | RabX10 |
| RabX10 | E. histolytica | EhiRabX10 | EHI_096440 | RabX10 |
| RabX17 | E. invadens | EinRabZ17 | EIN_341200 | RabX10 |
| RAXX7 | E. histolytica | EhiRaXX7 | EHI_040330 | RabX10 |
| RabX12 | E. invadens | EinRabX12 | EIN_424850 | RabX12 |
| RabX12 | E. histolytica | EhiRabX12 | EHI_021210 | RabX12 |
| RabZ9 | E. invadens | EinRabZ9 | EIN_476180 | RabX13 |
| RabX13 | E. histolytica | EhiRabX13 | EHI_065790 | RabX13 |
| RabX14 | E. invadens | EinRabX14A | EIN_438500 | RabX14 |
| RabX14 | E. invadens | EinRabX14B | EIN_250160 | RabX14 |
| RabX14 | E. histolytica | EhiRabX14 | EHI_053150 | RabX14 |
| RabX17 | E. invadens | EinRabX17A | EIN_118210 | RabX17 |
| RabX17 | E. invadens | EinRabX17B | EIN_359260 | RabX17 |
| RabX17 | E. invadens | EinRabX17C | EIN_175550 | RabX17 |
| RabX17 | E. histolytica | EhiRabX17 | EHI_042250 | RabX17 |
| RabZ16 | E. invadens | EinRabZ16 | EIN_117070 | RabX17 |
| RabX19 | E. invadens | EinRabX19 | EIN_485590 | RabX19 |
| RabX19 | E. histolytica | EhiRabX19 | EHI_183080 | RabX19 |
| RabX26 | E. invadens | EinRabX26A | EIN_380930 | RabX19 |
| RabX26 | E. invadens | EinRabX26B | EIN_097100 | RabX19 |
| RabX26 | E. histolytica | EhiRabX26 | EHI_151610 | RabX19 |
| RabX20 | E. histolytica | EhiRabX20 | EHI_003020, EHI_008640 | RabX20 |
| RabX21 | E. histolytica | EhiRabX21 | EHI_021480 | RabX21 |
| RAXX13 | E. histolytica | EhiRaxX13 | EHI_118020 | RabX21 |
| RAXX14 | E. histolytica | EhiRaxX14 | EHI_094000 | RabX21 |
| RAXX15 | E. histolytica | EhiRaxX15 | EHI_009450 | RabX21 |
| RAXX16 | E. histolytica | EhiRaxX16 | EHI_091090 | RabX21 |
| RABX22 | E. invadens | EinRabX22A | EIN_223700 | RabX22 |
| RABX22 | E. invadens | EinRabX22B | EIN_122260 | RabX22 |
| RABX22 | E. invadens | EinRabX22C | EIN_227490 | RabX22 |
| RABX22 | E. histolytica | EhiRabX22 | EHI_157890, EHI_014060 | RabX22 |
| RabX23 | E. invadens | EinRabX23 | EIN_408640 | RabX22 |
| RabX23 | E. histolytica | EhiRabX23 | EHI_107140 | RabX22 |
| RabX24 | E. histolytica | EhiRabX24 | EHI_038680 | RabX24 |
| RabX25 | E. invadens | EinRabX25 | EIN_411530 | RabX25 |
| RabX25 | E. histolytica | EhiRabX25 | EHI_010660 | RabX25 |
| RabX27 | E. histolytica | EhiRabX27 | EHI_158170 | RabX27 |
| RabX32 | E. histolytica | EhiRabX32 | EHI_079230 | RabX32 |
| RabX33 | E. histolytica | EhiRabX33 | EHI_083390, EHI_135940 | RabX33 |
| RabZ10 | E. invadens | EinRabZ10 | EIN_172650 | RabX33 |
| RabX100 | E. histolytica | EhiRabX100 | EHI5A_131260 | RabX33 |
| RabX34 | E. invadens | EinRabX34A | EIN_476200 | RabX34 |
| RabX34 | E. invadens | EinRabX34B | EIN_344150 | RabX34 |
| RabX34 | E. histolytica | EhiRabX34 | EHI_114640 | RabX34 |
| RabX35 | E. invadens | EinRabX35 | EIN_267670 | RabX35 |
| RabX35 | E. histolytica | EhiRabX35 | EHI_130670 | RabX35 |
| RabX40 | E. invadens | EinRabX40 | EIN_173190 | RabX40 |
| RabX40 | E. histolytica | EhiRabX40 | EHI_027640 | RabX40 |
| RabZ15 | E. invadens | EinRabZ15 | EIN_080230 | RabZ15 |
| RaxX10 | E. histolytica | EhiRaxX10 | EHI_045600 | RabZ15 |
| RabZ20 | E. invadens | EinRabZ20 | EIN_402870 | RabZ20 |
| RAXX11 | E. nuttali | EhiRaxX11 | ENU1_124830 | RabZ20 |
| RabZ4 | E. invadens | EinRabZ4A | EIN_501180 | RabZ4 |
| RabZ4 | E. invadens | EinRabZ4B | EIN_395670 | RabZ4 |
| RabZ19 | E. invadens | EinRabZ19 | EIN_026450 | RabZ19 |
| RabZ21 | E. invadens | EinRabZ21A | EIN_145520 | RabZ21 |
| RabZ22 | E. invadens | EinRabZ22 | EIN_280860 | RabZ22 |
| RabZ24 | E. invadens | EinRabZ24 | EIN_088900 | RabZ24 |
| RabX2 | E. invadens | EinRabX2A | EIN_224320 | RabX2 |
| RabX2 | E. invadens | EinRabX2B | EIN_160940 | RabX2 |
| RabX2 | E. histolytica | EhiRabX2 | EHI_001870 | RabX2 |
| RabX36 | E. histolytica | EhiRabX36 | EHI_110300 | RabX36 |
| RabX16 | E. invadens | EinRabX16 | EIN_080860 | RabX16 |
| RabX16 | E. histolytica | EhiRabX16 | EHI_131170 | RabX16 |
| RabZ5 | E. invadens | EinRabZ5 | EIN_062760 | RabZ5 |
| RabZ1 | E. invadens | EinRabZ1 | EIN_080140 | RabZ1 |
| RabZ6 | E. invadens | EinRabZ6 | EIN_080620 | RabZ6 |
| RabZ7 | E. invadens | EinRabZ7 | EIN_095070 | RabZ7 |
| RabZ23 | E. invadens | EinRabZ23 | EIN_265150 | RabZ23 |
| RabZ11 | E. invadens | EinRabZ11 | EIN_431200 | RabZ11 |

Alignment metadata

225 taxa, 150 informative positions

Best model according to AIC: LG+G+F

α: 1.137
