# Supplementary material for: Membrane Trafficking Modulation during Entamoeba Encystation
Source: Sci Rep. 2017 Oct 9;7:12854. doi: 10.1038/s41598-017-12875-6 (PMC5634486; doi:10.1038/s41598-017-12875-6)
Supplement: Supplementary file 8 — Table S7 [file 41598_2017_12875_MOESM8_ESM.doc]

Supplementary Table S7. Sequences used in global dynamin phylogeny, and in Drp1/Drp2 and Drp3/Drp4 phylogenies

Global dynamin phylogeny

| **Organism** | **Accession** | **Database** | **Assigned Function** | **Supergroup** |
| --- | --- | --- | --- | --- |
| Arabidopsis thaliana | NP_176252 | NCBI | Mx-protein | Archaeplastida |
| Arabidopsis thaliana | NP_851120 | NCBI | Cell plate formation | Archaeplastida |
| Arabidopsis thaliana | NP_172936 | NCBI | Cell plate formation | Archaeplastida |
| Arabidopsis thaliana | NP_567094 | NCBI | Disease resistance | Archaeplastida |
| Arabidopsis thaliana | NP_565363 | NCBI | Mitochondrial division | Archaeplastida |
| Arabidopsis thaliana | NP_850615 | NCBI | Chloroplast division | Archaeplastida |
| Arabidopsis thaliana | NP_175722 | NCBI | Cytokinesis | Archaeplastida |
| Batrachochytrium dendrobatidis | 89027 | JGI | Unknown function | Opisthokonta |
| Batrachochytrium dendrobatidis | 10517 | JGI | Mitochondrial fusion inner membrane | Opisthokonta |
| Batrachochytrium dendrobatidis | 35777 | JGI | Vacuolar sorting | Opisthokonta |
| Batrachochytrium dendrobatidis | 34336 | JGI | Mitochondrial fusion inner membrane | Opisthokonta |
| Chlamydomonas reinhardtii | XP_001700931 | NCBI | Cell plate formation | Archaeplastida |
| Chlamydomonas reinhardtii | XP_001697229 | NCBI | Mitochondrial division | Archaeplastida |
| Chlamydomonas reinhardtii | XP_001702662 | NCBI | Chloroplast division | Archaeplastida |
| Chlamydomonas reinhardtii | XP_001696853 | NCBI | Cytokinesis | Archaeplastida |
| Ciona intestinalis | XP_002130018 | NCBI | Clathrin mediated endocytosis | Opisthokonta |
| Ciona intestinalis | XP_002129 | NCBI | Mitochondrial and peroxisomal division | Opisthokonta |
| Ciona intestinalis | XP_002131 | NCBI | Mitochondrial fusion outer membrane | Opisthokonta |
| Cryptococcus neoformans | XP_569803 | NCBI | Mitochondrial fusion inner membrane | Opisthokonta |
| Cryptococcus neoformans | XP_566870 | NCBI | Vacuolar sorting | Opisthokonta |
| Cryptococcus neoformans | XP_569513 | NCBI | Mitochondrial division | Opisthokonta |
| Cryptococcus neoformans | XP_569513 | NCBI | Mitochondrial division | Opisthokonta |
| Cryptococcus neoformans | XP_571622 | NCBI | Mitochondrial fusion inner membrane | Opisthokonta |
| Cryptosporidium parvum | XP_627103 | NCBI | Apicoplast division clade | Alveolata |
| Cryptosporidium parvum | XP_001388141 | NCBI | Secretory pathway clade | Alveolata |
| Cryptosporidium parvum | XP_001388234 | NCBI | Unknown function | Alveolata |
| Cyanidioschyzon merolae | AAO23012 | NCBI | Mitochondrial division | Archaeplastida |
| Cyanidioschyzon merolae | BAC55068 | NCBI | Chloroplast division | Archaeplastida |
| Dictyostelium discoideum | DDB0215390 | Dictybase | Cytoskeleton | Amoebozoa |
| Dictyostelium discoideum | DDB0216177 | Dictybase | Mitochondrial division | Amoebozoa |
| Dictyostelium discoideum | DDB0229901 | Dictybase | Cytokinesis | Amoebozoa |
| Dictyostelium discoideum | DDB0302371 | Dictybase | Cytokinesis | Amoebozoa |
| Dictyostelium discoideum | DDB0302372 | Dictybase | Cytokinesis | Amoebozoa |
| Drosophila melanogaster | NP_732840 | NCBI | Mitochondrial fusion inner membrane | Opisthokonta |
| Drosophila melanogaster | NP_001162768 | NCBI | Clathrin mediated endocytosis | Opisthokonta |
| Drosophila melanogaster | AAN71025 | NCBI | Mitochondrial and peroxisomal division | Opisthokonta |
| Drosophila melanogaster | NP_610941 | NCBI | Mitochondrial fusion outer membrane | Opisthokonta |
| Emiliania huxleyi | 451602 | JGI | Unknown | Stramenopiles |
| Emiliania huxleyi | 461229 | JGI | No assigned function | Stramenopiles |
| Emiliania huxleyi | 96578 | JGI | Chloroplast division | Stramenopiles |
| Encephalitozoon cuniculi | NP_586287 | NCBI | Unknown function | Opisthokonta |
| Encephalitozoon cuniculi | XP_965959 | NCBI | Unknown function | Opisthokonta |
| Entamoeba histolytica | XP_651634 | NCBI | Unknown function | Amoebozoa |
| Entamoeba histolytica | XP_649650 | NCBI | Nuclear associated | Amoebozoa |
| Entamoeba histolytica | XP_653348 | NCBI | Cytokinesis clade | Amoebozoa |
| Entamoeba histolytica | XP_651307 | NCBI | Cytokinesis clade | Amoebozoa |
| Entamoeba invadens | EIN_114780 | AmoebaDB | Unknown function | Amoebozoa |
| Entamoeba invadens | EIN_428080 | AmoebaDB | Unknown function | Amoebozoa |
| Entamoeba invadens | EIN_081190 | AmoebaDB | Unknown function | Amoebozoa |
| Entamoeba invadens | EIN_080030 | AmoebaDB | Cytokinesis clade | Amoebozoa |
| Entamoeba invadens | EIN_376410 | AmoebaDB | Cytokinesis clade | Amoebozoa |
| Gallus gallus | XP_417640 | NCBI | Mitochondrial fusion inner membrane | Opisthokonta |
| Gallus gallus | BAC06346 | NCBI | Mx-protein | Opisthokonta |
| Gallus gallus | XP_415501 | NCBI | Clathrin mediated endocytosis | Opisthokonta |
| Gallus gallus | NP_001073190 | NCBI | Mitochondrial and peroxisomal division | Opisthokonta |
| Gallus gallus | NP_001034398 | NCBI | Mitochondrial fusion outer membrane | Opisthokonta |
| Giardia intestinalis | XP_00170496 | NCBI | Encystation specific vesicles | Excavata |
| Homo sapiens | NP_0055681 | NCBI | Mitochondrial fusion inner membrane | Opisthokonta |
| Homo sapiens | NP_002454 | NCBI | Mx-protein | Opisthokonta |
| Homo sapiens | NP_001005336 | NCBI | Clathrin mediated endocytosis | Opisthokonta |
| Homo sapiens | NP_005681 | NCBI | Mitochondrial and peroxisomal division | Opisthokonta |
| Homo sapiens | NP_056375 | NCBI | Mitochondrial fusion outer membrane | Opisthokonta |
| Leishmania major | XP_847885 | NCBI | Mitochondrial division | Excavata |
| Monosiga brevicollis | XP_001750100 | NCBI | Unknown function | Opisthokonta |
| Monosiga brevicollis | XP_001749319 | NCBI | Clathrin mediated endocytosis | Opisthokonta |
| Monosiga brevicollis | XP_001750431 | NCBI | Mitochondrial and peroxisomal division | Opisthokonta |
| Monosiga brevicollis | XP_001746637 | NCBI | Mitochondrial fusion outer membrane | Opisthokonta |
| Naegleria gruberi | XP_002670416 | NCBI | Unknown function | Excavata |
| Naegleria gruberi | XP_002683124 | NCBI | Unknown function | Excavata |
| Naegleria gruberi | XP_002681690 | NCBI | Unknown function | Excavata |
| Naegleria gruberi | XP_002683545 | NCBI | Cytokinesis clade | Excavata |
| Oryza sativa | NP_001049617 | NCBI | Mx-protein | Archaeplastida |
| Oryza sativa | CAE02378 | NCBI | Mx-protein | Archaeplastida |
| Oryza sativa | NP_001064002 | NCBI | Cell plate formation | Archaeplastida |
| Oryza sativa | NP_001064002 | NCBI | Cell plate formation | Archaeplastida |
| Oryza sativa | AC090882 | NCBI | Cell plate formation | Archaeplastida |
| Oryza sativa | NP_001065436 | NCBI | Cell plate formation | Archaeplastida |
| Oryza sativa | NP_001052600 | NCBI | Mitochondrial division | Archaeplastida |
| Oryza sativa | NP_001045220 | NCBI | Mitochondrial division | Archaeplastida |
| Oryza sativa | EEE52865 | NCBI | Chloroplast division | Archaeplastida |
| Oryza sativa | BAD86966 | NCBI | Cytokinesis | Archaeplastida |
| Phycomyces blakesleeanus | 62295 | JGI | Mitochondrial fusion inner membrane | Opisthokonta |
| Phycomyces blakesleeanus | 41775 | JGI | Mitochondrial fusion inner membrane | Opisthokonta |
| Phycomyces blakesleeanus | 30271 | JGI | Mitochondrial fusion inner membrane | Opisthokonta |
| Phycomyces blakesleeanus | 37829 | JGI | Vacuolar sorting | Opisthokonta |
| Phycomyces blakesleeanus | 57948 | JGI | Vacuolar sorting | Opisthokonta |
| Phycomyces blakesleeanus | 32212 | JGI | Mitochondrial division | Opisthokonta |
| Phycomyces blakesleeanus | 20253 | JGI | Mitochondrial division | Opisthokonta |
| Phycomyces blakesleeanus | 10554 | JGI | Mitochondrial fusion inner membrane | Opisthokonta |
| Physcomitrella patens | XP_001773410 | NCBI | Cell plate formation | Archaeplastida |
| Physcomitrella patens | XP_001766173 | NCBI | Mitochondrial division | Archaeplastida |
| Physcomitrella patens | XP_001758540 | NCBI | Mitochondrial division | Archaeplastida |
| Physcomitrella patens | XP_001779783 | NCBI | Chloroplast division | Archaeplastida |
| Physcomitrella patens | XP_001781521 | NCBI | Chloroplast division | Archaeplastida |
| Physcomitrella patens | XP_001759610 | NCBI | Chloroplast division | Archaeplastida |
| Physcomitrella patens | XP_001752691 | NCBI | Cytokinesis | Archaeplastida |
| Phythophtora ramorum | 86760 | JGI | Unknown function | Alveolata |
| Phythophtora ramorum | 72119 | JGI | Unknown function | Alveolata |
| Plasmodium falciparum | XP_001350587 | NCBI | Unknown function | Alveolata |
| Plasmodium falciparum | XP_001348132 | NCBI | Kinetoplast division clade | Alveolata |
| Plasmodium falciparum | XP_001347652 | NCBI | Secretory pathway clade | Alveolata |
| Polysphondylium pallidum | EFA84816 | NCBI | Cytoskeleton | Amoebozoa |
| Polysphondylium pallidum | EFA75434 | NCBI | Mitochondrial division | Amoebozoa |
| Polysphondylium pallidum | EFA84098 | NCBI | Cytokinesis | Amoebozoa |
| Polysphondylium pallidum | EFA76312 | NCBI | Cytokinesis | Amoebozoa |
| Polysphondylium pallidum | EFA76188 | NCBI | Cytokinesis | Amoebozoa |
| Saccharomyces cerevisiae | NP_014854 | NCBI | Mitochondrial fusion inner membrane | Opisthokonta |
| Saccharomyces cerevisiae | NP_012926 | NCBI | Vacuolar sorting | Opisthokonta |
| Saccharomyces cerevisiae | NP_013100 | NCBI | Mitochondrial division | Opisthokonta |
| Saccharomyces cerevisiae | NP_009738 | NCBI | Mitochondrial fusion inner membrane | Opisthokonta |
| Tetrahymena thermophila | ABB13594 | NCBI | Macronuclear remodelling | Alveolata |
| Tetrahymena thermophila | ABB13593 | NCBI | Macronuclear remodelling | Alveolata |
| Tetrahymena thermophila | XP_001029982 | NCBI | Macronuclear remodelling | Alveolata |
| Tetrahymena thermophila | XP_001029980 | NCBI | Macronuclear remodelling | Alveolata |
| Tetrahymena thermophila | ABB13592 | NCBI | Unknown function | Alveolata |
| Tetrahymena thermophila | ABB13595 | NCBI | Clathrin mediated endocytosis | Alveolata |
| Tetrahymena thermophila | XP_001009829 | NCBI | Clathrin mediated endocytosis | Alveolata |
| Thalassiosira pseudonana | XP_002296064 | NCBI | Mitochondrial division | Stramenopiles |
| Thalassiosira pseudonana | XP_002290986 | NCBI | Chloroplast division | Stramenopiles |
| Theileria annulata | XP_952965 | NCBI | Apicoplast division clade | Alveolata |
| Theileria annulata | XP_764712 | NCBI | Secretory pathway clade | Alveolata |
| Toxoplasma gondii | EEA98594 | NCBI | Unknown function | Alveolata |
| Toxoplasma gondii | EEB01708 | NCBI | Apicoplast division | Alveolata |
| Toxoplasma gondii | EEB04563 | NCBI | Secretory pathway | Alveolata |
| Trichomonas vaginalis | XP_001581 | NCBI | Unknown function | Excavata |
| Trichomonas vaginalis | XP_001308 | NCBI | Unknown function | Excavata |
| Trichomonas vaginalis | XP_001316 | NCBI | Unknown function | Excavata |
| Trichomonas vaginalis | XP_001579 | NCBI | Unknown function | Excavata |
| Trichomonas vaginalis | XP_001305 | NCBI | Unknown function | Excavata |
| Trichomonas vaginalis | XP_001311 | NCBI | Unknown function | Excavata |
| Trichomonas vaginalis | XP_001324 | NCBI | Unknown function | Excavata |
| Trichomonas vaginalis | XP_00132 | NCBI | Unknown function | Excavata |
| Trypanosoma brucei | XP_844064 | NCBI | Mitochondrial division | Excavata |

Drp1/Drp2 phylogeny

| **Organism** | **Accession** | **Database** | **Assigned Function** | **Supergroup** |
| --- | --- | --- | --- | --- |
| Giardia intestinalis | XP_001704962 | NCBI | Stage conversion/endocytosis | Excavata |
| Spironucleus barkhanus | ABI15596 | NCBI | Stage conversion/endocytosis | Excavata |
| Trichomonas vaginalis | XP_001308653 | NCBI | Unknown | Excavata |
| Trichomonas vaginalis | XP_001316433 | NCBI | Unknown | Excavata |
| Trichomonas vaginalis | XP_001579617 | NCBI | Unknown | Excavata |
| Trichomonas vaginalis | XP_001305587 | NCBI | Unknown | Excavata |
| Trichomonas vaginalis | XP_001311094 | NCBI | Unknown | Excavata |
| Trichomonas vaginalis | XP_001324893 | NCBI | Unknown | Excavata |
| Trichomonas vaginalis | XP_001324471 | NCBI | Unknown | Excavata |
| Plasmodium falciparum | XP_001347652 | NCBI | Mitochondrial division | Alveolata |
| Leishmania major | XP_847885 | NCBI | Mitochondrial division | Alveolata |
| Trypanosoma brucei | AAN05457 | NCBI | Mitochondrial division and endocytosis | Alveolata |
| Entamoeba histolytica | XP_649650 | NCBI | Unknown function | Amoebozoa |
| Entamoeba histolytica | XP_651634 | NCBI | Unknown function | Amoebozoa |
| Entamoeba moshkovskii | mosh054c06.p1k + mosh030g01.p1k | NCBI GSS database | Unknown function | Amoebozoa |
| Entamoeba invadens | EIN_051430 | AmoebaDB | Unknown function | Amoebozoa |
| Entamoeba invadens | EIN_070060 | AmoebaDB | Unknown function | Amoebozoa |
| Entamoeba invadens | EIN_254810 | AmoebaDB | Unknown function | Amoebozoa |
| Entamoeba terrapinae | terra244d12.q1k | NCBI GSS database | Unknown function | Amoebozoa |
| Entamoeba dispar | XP_001734031 | NCBI | Unknown function | Amoebozoa |
| Entamoeba dispar | XP_001735320 | NCBI | Unknown function | Amoebozoa |
| Tetrahymena thermophila | ABB13595 | NCBI | Clathrin mediated endoyctosis | Alveolata |
| Tetrahymena thermophila | XP_001009829 | NCBI | Clathrin mediated endocytosis | Alveolata |
| Chlamydomonas reinhardtii | XP_001697229 | NCBI | Mitochondrial division | Archaeplastida |
| Monosiga brevicollis | XP_001749319 | NCBI | Clathrin mediated endocytosis | Opisthokonta |
| Drosophila melanogaster | NP_001162768 | NCBI | Clathrin mediated endocytosis | Opisthokonta |
| Ciona intestinalis | XP_002130319 | NCBI | Clathrin mediated endocytosis | Opisthokonta |
| Homo sapiens | NP_001005336 | NCBI | Clathrin mediated endocytosis | Opisthokonta |
| Gallus gallus | XP_415501 | NCBI | Clathrin mediated endocytosis | Opisthokonta |
| Cryptosporidium parvum | XP_001388234 | NCBI | Unknown function | Opisthokonta |
| Polysphondylium pallidum | EFA84816 | NCBI | Cytoskeleton | Amoebozoa |
| Dictyostelium discoideum | XP_642447 | NCBI | Cytoskeleton | Amoebozoa |
| Encephalitozoon cuniculi | NP_586287 | NCBI | Vacuolar sorting clade | Opisthokonta |
| Neurospora crassa | XP_002995380 | NCBI | Vacuolar sorting clade | Opisthokonta |
| Monosiga brevicollis | XP_001750431 | NCBI | Mitochondrial and peroxisomal division | Opisthokonta |
| Cyanidioschyzon merolae | AAO23012 | NCBI | Mitochondrial division | Archaeplastida |
| Oryza sativa | NP_001052600 | NCBI | Mitochondrial division | Archaeplastida |
| Physcomitrella patens | XP_001766173 | NCBI | Mitochondrial division | Archaeplastida |
| Physcomitrella patens | XP_001777446 | NCBI | Mitochondrial division | Archaeplastida |
| Physcomitrella patens | XP_001775158 | NCBI | Mitochondrial division | Archaeplastida |
| Physcomitrella patens | XP_001761534 | NCBI | Mitochondrial division | Archaeplastida |
| Arabidopsis thaliana | NP_565363 | NCBI | Mitochondrial division | Archaeplastida |
| Oryza sativa | NP_001045220 | NCBI | Mitochondrial division | Archaeplastida |
| Theileria annulata | XP_764712 | NCBI | Secretory pathway | Alveolata |
| Toxoplasma gondii | EEE27889 | NCBI | Secretory pathway | Alveolata |
| Saccharomyces cerevisiae | NP_012926 | NCBI | Vacuolar sorting | Opisthokonta |
| Cryptococcus neoformans | XP_566870 | NCBI | Vacuolar sorting | Opisthokonta |
| Batrachochytrium dendrobatidis | 35777 | JGI | Vacuolar sorting | Opisthokonta |
| Phycomyces blakesleeanus | 37829 | JGI | Vacuolar sorting | Opisthokonta |
| Phycomyces blakesleeanus | 57948 | JGI | Vacuolar sorting | Opisthokonta |
| Saccharomyces cerevisiae | NP_013100 | NCBI | Mitochondrial division | Opisthokonta |
| Cryptococcus neoformans | XP_569513 | NCBI | Mitochondrial division | Opisthokonta |
| Phycomyces blakesleeanus | 32212 | JGI | Mitochondrial division | Opisthokonta |
| Phycomyces blakesleeanus | 20253 | JGI | Mitochondrial division | Opisthokonta |
| Ciona intestinalis | XP_002130018 | NCBI | Clathrin mediated endocytosis | Opisthokonta |
| Drosophila melanogaster | AAN71025 | NCBI | Mitochondrial and peroxisomal division | Opisthokonta |
| Gallus gallus | NP_001073190 | NCBI | Mitochondrial and peroxisomal division | Opisthokonta |
| Homo sapiens | NP_005681 | NCBI | Mitochondrial and peroxisomal division | Opisthokonta |
| Pinnularia infestans | XP_002908808 | NCBI | Mitochondrial division | Stramenopiles |
| Naegleria gruberi | XP_002683124 | NCBI | Unknown function | Excavata |
| Naegleria gruberi | XP_002681690 | NCBI | Unknown function | Excavata |
| Polysphondylium pallidum | EFA75434 | NCBI | Mitochondrial division | Amoebozoa |
| Dictyostelium discoideum | DDB_G0277849 | Dictybase | Mitochondrial division | Amoebozoa |
| Thalassiosira pseudonana | XP_002296064 | NCBI | Mitochondrial division | Stramenopiles |
| Phytophthora ramorum | XP_002908808 | NCBI | Mitochondrial division | Stramenopiles |
| Phaeodactylum tricornutum | XP_002181636 | NCBI | Mitochondrial division | Stramenopiles |

Drp3/Drp4 phylogeny

| **Organism** | **Accession** | **Database** | **Assigned Function** | **Supergroup** |
| --- | --- | --- | --- | --- |
| Entamoeba histolytica Drp3 | XP_653348 | NCBI | Unknown function | Amoebozoa |
| Entamoeba histolytica Drp4 | XP_651307 | NCBI | Unknown function | Amoebozoa |
| Entamoeba moshkovskii Drp3 | mosh088g11 | NCBI GSS | Unknown function | Amoebozoa |
|  | mosh088g11 | NCBI GSS |  |  |
|  | mosh077g07 | NCBI GSS |  |  |
|  | mosh049f11 | NCBI GSS |  |  |
| Entamoeba moshkovskii Drp4 | EMO_007670 | AmoebaDB | Unknown function | Amoebozoa |
| Entamoeba invadens Drp3 | EIN_376410 | AmoebaDB | Unknown function | Amoebozoa |
| Entamoeba invadens Drp4 | EIN_080030 | AmoebaDB | Unknown function | Amoebozoa |
| Entamoeba dispar Drp3 | XP_001740444 | NCBI | Unknown function | Amoebozoa |
| Entamoeba dispar Drp4 | XP_001733630 | NCBI | Unknown function | Amoebozoa |
| Chlamydomonas reinhardtii Drp5A | XP_001700931 | NCBI | Cytokinesis | Archaeplastida |
| Chlamydomonas reinhardtii Drp5B | XP_001702662 | NCBI | Chloroplast division | Archaeplastida |
| Polysphondylium pallidum DlpA | EFA84098 | NCBI | Cytokinesis | Amoebozoa |
| Polysphondylium pallidum DlpB | EFA76312 | NCBI | Cytokinesis | Amoebozoa |
| Polysphondylium pallidum DlpC | EFA76188 | NCBI | Cytokinesis | Amoebozoa |
| Dictyostelium discoideum DlpA | DDBG0268592 | Dictybase | Cytokinesis | Amoebozoa |
| Dictyostelium discoideum DlpB | DDBG0285931 | Dictybase | Cytokinesis | Amoebozoa |
| Dictyostelium discoideum DlpC | DDBG0271628 | Dictybase | Cytokinesis | Amoebozoa |
| Cyanidioschyzon merolae Drp5B | BAC55068 | NCBI | Chloroplast division | Archaeplastida |
| Oryza sativa Drp5A | BAD86966 | NCBI | Cytokinesis | Archaeplastida |
| Oryza sativa Drp5B | ABA96623 | NCBI | Chloroplast division | Archaeplastida |
| Physcomitrella patens Drp5A | XP_001773410 | NCBI | Cytokinesis | Archaeplastida |
| Physcomitrella patens | XP_001759610 | NCBI | Chloroplast division | Archaeplastida |
| Physcomitrella patens | XP_001781521 | NCBI | Chloroplast division | Archaeplastida |
| Physcomitrella patens | XP_001779783 | NCBI | Chloroplast division | Archaeplastida |
| Arabidopsis thaliana Drp5A | NP_175722 | NCBI | Cytokinesis | Archaeplastida |
| Arabidopsis thaliana Drp5B | NP_850615 | NCBI | Chloroplast division | Archaeplastida |
| Naegleria gruberi | XP_002683545 | NCBI | Unknown function | Excavata |
| Thalassiosira pseudonana Drp5B | XP_002290986 | NCBI | Chloroplast division | Stramenopiles |
| Phaeodactylum tricornutum Drp5B | XP_002181459 | NCBI | Chloroplast division | Stramenopiles |
| Ectocarpus siliculosus Drp5B | CBN78455 | NCBI | Chloroplast division | Stramenopiles |
