# Supplementary material for: Membrane Trafficking Modulation during Entamoeba Encystation
Source: Sci Rep. 2017 Oct 9;7:12854. doi: 10.1038/s41598-017-12875-6 (PMC5634486; doi:10.1038/s41598-017-12875-6)
Supplement: Supplementary file 9 — Python script [file 41598_2017_12875_MOESM9_ESM.pdf]

```
###Trim.slidingWindow.pl script

#!/usr/bin/perl -w

# AUTHOR: Joseph Fass
# LAST REVISED: September 2010
#
# The Bioinformatics Core at UC Davis Genome Center
# http://bioinformatics.ucdavis.edu
# Copyright (c) 2010 The Regents of University of California, Davis Campus.
# All rights reserved.

# trims Sanger fastq sequences using a sliding window

use Getopt::Std;
$usage = "\nusage: cat <sequences.fastq> | perl $0\n\n".
         "$0 uses a sliding window of length (int(seqLength/10)) to quality-trim Illumina read 3' ends\n\n".
         "options:\n".
         "-q #     trim starting at first base with quality < #, in first sliding window with mean quality < #\n".
         "-l #     discard trimmed sequences shorter than length #\n\n";
getopts('q:l:') or die $usage;
if (!defined($opt_q) or !($opt_q =~ /^[0-9]+$/)) { $opt_q = 20 }
if (!defined($opt_l) or !($opt_l =~ /^[0-9]+$/)) { $opt_l = 20 }

#print STDERR "q $opt_q\tl $opt_l\n";

READ: while (<>) {
        $head1 = $_;
        $seq = <>;
        # stop processing read if it's too short anyway
        next READ if (length($seq)-1 <= $opt_l);  # -1 to account for newline
        $head2 = <>;
        $qual = <>;
        chomp $qual;
        @Qchr = split(//,$qual);
        undef @Qval;
        # create array of base PHRED scores
        for ($i=0; $i<=$#Qchr; $i++) {
                push @Qval, ord($Qchr[$i]) - 33;
        }

# print join("\t",@Qval)."\n";
        # define window length
        $winLength = int( length($qual) / 10 );
        # advance window, testing for mean quality lower than cutoff
        # for read length 20, $#Qval would be 19
        # and for winLength 5, last window should start at 16
        # thus $i <= 19 - 5 + 1 = 15 (index of 16th array element)
        WIN: for ($i=0; $i<=$#Qval-$winLength+1; $i++) {
                $meanPHRED = 0;
                $trimStart = -1;
                # sum PHRED scores in window, and set possible local trim point
                for ($j=$i; $j<=$i+$winLength-1; $j++) {
                        $meanPHRED += $Qval[$j];
                        # set position of first bad base in this window
                        if ($trimStart<0 and $Qval[$j]<$opt_q) {
                                $trimStart = $j;  # index of first base to trim
                        }
                }
                $meanPHRED = $meanPHRED / $winLength;
#print "$meanPHRED\n";
                # terminate loop if bad window detected
                last WIN if ($meanPHRED < $opt_q);
        }
        # if and only if $trimStart was set, the loop must have terminated on a bad window
        if ($trimStart >= 0) {
                # if read woudn't be trimmed shorter than cutoff length ...
                # note zero-indexing trickiness ... if $trimStart = 5
```

```
		# that means 6th base and beyond should be trimmed
		# leaving a 5 base read (base positions 0,1,2,3,4)
		# so comparison should be: if ($trimStart-1+1 >= $opt_l) ...
		if ($trimStart >= $opt_l) {
			print $head1;

# print $seq;
			print substr($seq,0,$trimStart)."\n";
			print $head2;
# print $qual."\n";
			print substr($qual,0,$trimStart)."\n";
		}
	}
	# else there must have been no bad windows
	else {
		# check in this last window for possible local trim point
		$trimStart = -1;
		LAST: for ($j=$#Qval-$winLength+1; $j<=$#Qval; $j++) {
			if ($Qval[$j]<$opt_q) {
				$trimStart = $j;  # index of first bad base in window
				last LAST;  # end for loop with first bad base detected
			}
		}
		# now, if there's a local trim point
		if ($trimStart >= 0) {
			if ($trimStart >= $opt_l) {
				print $head1;
				print substr($seq,0,$trimStart)."\n";
				print $head2;
				print substr($qual,0,$trimStart)."\n";
			}
		}
		# no local trim
		else { print $head1.$seq.$head2.$qual."\n" }
	}
}
```
