# Supplementary material for: Membrane Trafficking Modulation during Entamoeba Encystation
Source: Sci Rep. 2017 Oct 9;7:12854. doi: 10.1038/s41598-017-12875-6 (PMC5634486; doi:10.1038/s41598-017-12875-6)
Supplement: Supplementary file 11 — Tree file [file 41598_2017_12875_MOESM11_ESM.pdf]

```
#NEXUS
begin taxa;
    dimensions ntax=225;
    taxlabels
        EinRabZ14
        EhiRaxX16
        EhiRaxX15
        EhiRaxX13
        EhiRaxX14
        EhiRaxX12
        EhiRaxX10
        EhiRaxX11
        EinRabX3
        EinRabZ11
        EhiRaxX9
        EhiRabX36
        EinRabZ20
        EhiRabX29
        EinRabX29
        EinRabZ24
        EinRabZ17
        EhiRaxX7
        EhiRabX8
        EhiRabX27
        EhiRabX4
        EinRabX4
        EinRabZ12
        EinRabZ26
        EinRabZ8B
        EinRabZ8A
        EinRabX7
        EhiRabX7
        EhiRabX6
        EinRabX6
        EinRabZ16
        EinRabX17C
        EinRabX17B
        EhiRabX17
        EinRab17A
        EinRabX40
        EhiRabX21
        EinRabX41
        EinRabZ22
        EinRabZ21A
        EinRabZ4B
        EinRabZ4A
        EinRabZ15
        EinRabZ10
        EhiRabX100
        EhiRabX33
        EhiRabX32
        EinRabZ23
        EhiRabX10
        EinRabX10
        EhiRabX5
        EinRabZ13
        EinRabX31C
        EinRabX31D
        EhiRabX31
        EinRabX31A
        EinRabX31B
        EhiRAN
        EinRabZ1
        EinRabL2
        EinRabL1
        EhiRab32a
        EhiRabL1
        EinRabX11C
        EinRabX11B
        EhiRabX11
        EinRabX11A
```

EhiRabX35
EinRabX35
EhiRabX16
EinRabX16
EhiRabX20
EhiRabX25
EinRabX25
EhiRabI2
EhiRabI3
EhiRabI1
EinRabI
EhiRab21
EhiRabA
EinRabA
EhiRabH
EinRabH
EhiRabK4
EinRabK4
EhiRabK3
EhiRabK1
EinRabK1
EhiRabK2
EinRabK2
EinRabK5
EhiRabK5
EhiRabX30
EinRabX30
EinRabZ18
EinRabP
EhiRabP1
EhiRabP2
EinRabX39
EhiRabP
EinRabZ19
EhiRabX2
EinRabX2B
EinRabX2A
EinRab7H
EhiRab7H
EhiRab7F
EinRab7F
EhiRab7A
EinRab7A
EinRab7G2
EhiRab7G
EinRab7G1
EhiRab7I
EinRab7I
EhiRab7D
EinRab7D
EhiRab7B
EinRab7B
EhiRab7E
EinRab7E
EhiRab7C
EinRab7C1
EinRabZ25
EhiRabX18
EinRabZ5
EinRabX14A
EinRabX14B
EhiRabX14
EhiRabM1
EinRabM1
EhiRabM2
EhiRabM3
EinRabM3
EinRabX19
EhiRabX19
EinRabX26B
EhiRabX26

143 EinRabX26
144 EhiRabX24
145 EhiRabX23
146 EinRabX23
147 EinRabX22C
148 EinRabX22B
149 EhiRabX22
150 EinRabX22A
151 EinRabN2
152 EinRabN1
153 EhiRabN2
154 EhiRabN1
155 EinRab5B
156 EhiRab5
157 EinRab5A
158 EinRabZ2B
159 EinRabZ2A
160 EinRabZ3
161 EhiRabD2
162 EhiRabD3
163 EinRabD
164 EhiRabD1
165 EhiRabB
166 EinRabB
167 EhiRabC8
168 EinRabC8
169 EhiRabC7
170 EinRabC7
171 EhiRabC6
172 EhiRabC2
173 EinRabC2
174 EhiRabC5
175 EinRabC5
176 EhiRabC1
177 EinRabC1
178 EinRabC3B
179 EhiRabC3
180 EinRabC3A
181 EhiRabC4
182 EinRabC4
183 EinRabZ7
184 EinRabZ9
185 EinRab2B
186 EhiRab2B
187 EhiRab2C
188 EhiRab2A
189 EinRab2A
190 EhiRab11C
191 EinRab11C
192 EinRab11D
193 EhiRab11D
194 EhiRab11A
195 EinRab11A
196 EhiRab11B
197 EinRab11B
198 EhiRabX12
199 EinRabX12
200 EhiRabX13
201 EhiRab8A
202 EinRab8A
203 EhiRAB8B
204 EinRab8B
205 EinRabX34B
206 EhiRabX34
207 EinRabX34A
208 EhiRab1A
209 EinRab1A
210 EhiRab1B
211 EinRab1B
212 EinRabX1A
213 EinRabX1B

```
        EhiRabX1
        EhiRabX15
        EinRabX15
        EinRabX9
        EhiRabX9
        EinRabZ6
        EhiRabF5
        EinRabF5
        EhiRabF1
        EinRabF1
        EhiRabF2
        EinRabF2
        EhiRabF3
        EinRabF3
        EhiRabF4
        EinRabF4
        ;
end;
begin trees;
    translate
        1   EinRabZ14,
        2   EhiRaxX16,
        3   EhiRaxX15,
        4   EhiRaxX13,
        5   EhiRaxX14,
        6   EhiRaxX12,
        7   EhiRaxX10,
        8   EhiRaxX11,
        9   EinRabX3,
        10  EinRabZ11,
        11  EhiRaxX9,
        12  EhiRabX36,
        13  EinRabZ20,
        14  EhiRabX29,
        15  EinRabX29,
        16  EinRabZ24,
        17  EinRabZ17,
        18  EhiRaxX7,
        19  EhiRabX8,
        20  EhiRabX27,
        21  EhiRabX4,
        22  EinRabX4,
        23  EinRabZ12,
        24  EinRabZ26,
        25  EinRabZ8B,
        26  EinRabZ8A,
        27  EinRabX7,
        28  EhiRabX7,
        29  EhiRabX6,
        30  EinRabX6,
        31  EinRabZ16,
        32  EinRabX17C,
        33  EinRabX17B,
        34  EhiRabX17,
        35  EinRab17A,
        36  EinRabX40,
        37  EhiRabX21,
        38  EinRabX41,
        39  EinRabZ22,
        40  EinRabZ21A,
        41  EinRabZ4B,
        42  EinRabZ4A,
        43  EinRabZ15,
        44  EinRabZ10,
        45  EhiRabX100,
        46  EhiRabX33,
        47  EhiRabX32,
        48  EinRabZ23,
        49  EhiRabX10,
        50  EinRabX10,
        51  EhiRabX5,
```

```
52  EinRabZ13,
53  EinRabX31C,
54  EinRabX31D,
55  EhiRabX31,
56  EinRabX31A,
57  EinRabX31B,
58  EhiRAN,
59  EinRabZ1,
60  EinRabL2,
61  EinRabL1,
62  EhiRab32a,
63  EhiRabL1,
64  EinRabX11C,
65  EinRabX11B,
66  EhiRabX11,
67  EinRabX11A,
68  EhiRabX35,
69  EinRabX35,
70  EhiRabX16,
71  EinRabX16,
72  EhiRabX20,
73  EhiRabX25,
74  EinRabX25,
75  EhiRabI2,
76  EhiRabI3,
77  EhiRabI1,
78  EinRabI,
79  EhiRab21,
80  EhiRabA,
81  EinRabA,
82  EhiRabH,
83  EinRabH,
84  EhiRabK4,
85  EinRabK4,
86  EhiRabK3,
87  EhiRabK1,
88  EinRabK1,
89  EhiRabK2,
90  EinRabK2,
91  EinRabK5,
92  EhiRabK5,
93  EhiRabX30,
94  EinRabX30,
95  EinRabZ18,
96  EinRabP,
97  EhiRabP1,
98  EhiRabP2,
99  EinRabX39,
100 EhiRabP,
101 EinRabZ19,
102 EhiRabX2,
103 EinRabX2B,
104 EinRabX2A,
105 EinRab7H,
106 EhiRab7H,
107 EhiRab7F,
108 EinRab7F,
109 EhiRab7A,
110 EinRab7A,
111 EinRab7G2,
112 EhiRab7G,
113 EinRab7G1,
114 EhiRab7I,
115 EinRab7I,
116 EhiRab7D,
117 EinRab7D,
118 EhiRab7B,
119 EinRab7B,
120 EhiRab7E,
121 EinRab7E,
122 EhiRab7C,
```

```
123 EinRab7C1,
124 EinRabZ25,
125 EhiRabX18,
126 EinRabZ5,
127 EinRabX14A,
128 EinRabX14B,
129 EhiRabX14,
130 EhiRabM1,
131 EinRabM1,
132 EhiRabM2,
133 EhiRabM3,
134 EinRabM3,
135 EinRabX19,
136 EhiRabX19,
137 EinRabX26B,
138 EhiRabX26,
139 EinRabX26,
140 EhiRabX24,
141 EhiRabX23,
142 EinRabX23,
143 EinRabX22C,
144 EinRabX22B,
145 EhiRabX22,
146 EinRabX22A,
147 EinRabN2,
148 EinRabN1,
149 EhiRabN2,
150 EhiRabN1,
151 EinRab5B,
152 EhiRab5,
153 EinRab5A,
154 EinRabZ2B,
155 EinRabZ2A,
156 EinRabZ3,
157 EhiRabD2,
158 EhiRabD3,
159 EinRabD,
160 EhiRabD1,
161 EhiRabB,
162 EinRabB,
163 EhiRabC8,
164 EinRabC8,
165 EhiRabC7,
166 EinRabC7,
167 EhiRabC6,
168 EhiRabC2,
169 EinRabC2,
170 EhiRabC5,
171 EinRabC5,
172 EhiRabC1,
173 EinRabC1,
174 EinRabC3B,
175 EhiRabC3,
176 EinRabC3A,
177 EhiRabC4,
178 EinRabC4,
179 EinRabZ7,
180 EinRabZ9,
181 EinRab2B,
182 EhiRab2B,
183 EhiRab2C,
184 EhiRab2A,
185 EinRab2A,
186 EhiRab11C,
187 EinRab11C,
188 EinRab11D,
189 EhiRab11D,
190 EhiRab11A,
191 EinRab11A,
192 EhiRab11B,
193 EinRab11B,
```

```
		194 EhiRabX12,
		195 EinRabX12,
		196 EhiRabX13,
		197 EhiRab8A,
		198 EinRab8A,
		199 EhiRAB8B,
		200 EinRab8B,
		201 EinRabX34B,
		202 EhiRabX34,
		203 EinRabX34A,
		204 EhiRab1A,
		205 EinRab1A,
		206 EhiRab1B,
		207 EinRab1B,
		208 EinRabX1A,
		209 EinRabX1B,
		210 EhiRabX1,
		211 EhiRabX15,
		212 EinRabX15,
		213 EinRabX9,
		214 EhiRabX9,
		215 EinRabZ6,
		216 EhiRabF5,
		217 EinRabF5,
		218 EhiRabF1,
		219 EinRabF1,
		220 EhiRabF2,
		221 EinRabF2,
		222 EhiRabF3,
		223 EinRabF3,
		224 EhiRabF4,
		225 EinRabF4
		;
	tree con_all_compat = [&U]
	(1[&prob=1.00000000e+00,prob_stddev=0.00000000e+00,prob_range={1.00000000e+00,1.000
	00000e+00},prob(percent)="100",prob+-sd="100+-0"]:3.269261e+00[&length_mean=3.31465
	504e+00,length_median=3.26926100e+00,length_95%HPD={2.17482700e+00,4.56492600e+00}]
	,(59[&prob=1.00000000e+00,prob_stddev=0.00000000e+00,prob_range={1.00000000e+00,1.0
	0000000e+00},prob(percent)="100",prob+-sd="100+-0"]:5.910569e-01[&length_mean=5.624
	58413e-01,length_median=5.91056900e-01,length_95%HPD={7.71098400e-02,9.42141100e-01
	}],((((60[&prob=1.00000000e+00,prob_stddev=0.00000000e+00,prob_range={1.00000000e+0
	0,1.00000000e+00},prob(percent)="100",prob+-sd="100+-0"]:4.571846e-01[&length_mean=
	4.61764508e-01,length_median=4.57184600e-01,length_95%HPD={2.76746100e-01,6.5159040
	0e-01}],61[&prob=1.00000000e+00,prob_stddev=0.00000000e+00,prob_range={1.00000000e+
	00,1.00000000e+00},prob(percent)="100",prob+-sd="100+-0"]:2.483711e-01[&length_mean
	=2.53390202e-01,length_median=2.48371100e-01,length_95%HPD={1.42208200e-01,3.738706
	00e-01}])[&prob=4.66404479e-01,prob_stddev=1.59313486e-02,prob_range={4.55139315e-0
	1,4.77669644e-01},prob(percent)="47",prob+-sd="47+-2"]:5.263692e-02[&length_mean=5.
	98294867e-02,length_median=5.26369200e-02,length_95%HPD={1.34752500e-04,1.33642200e
	-01}],62[&prob=1.00000000e+00,prob_stddev=0.00000000e+00,prob_range={1.00000000e+00
	,1.00000000e+00},prob(percent)="100",prob+-sd="100+-0"]:1.948590e-01[&length_mean=1
	.99122326e-01,length_median=1.94859000e-01,length_95%HPD={1.03861400e-01,2.98341700
	e-01}])[&prob=4.95933875e-01,prob_stddev=5.42985610e-02,prob_range={4.57538995e-01,
	5.34328756e-01},prob(percent)="50",prob+-sd="50+-5"]:7.192964e-02[&length_mean=7.74
	054754e-02,length_median=7.19296400e-02,length_95%HPD={5.62152300e-03,1.56461300e-0
	1}],63[&prob=1.00000000e+00,prob_stddev=0.00000000e+00,prob_range={1.00000000e+00,1
	.00000000e+00},prob(percent)="100",prob+-sd="100+-0"]:1.931650e-01[&length_mean=1.9
	7744690e-01,length_median=1.93165000e-01,length_95%HPD={8.64644000e-02,3.13666400e-
	01}])[&prob=1.00000000e+00,prob_stddev=0.00000000e+00,prob_range={1.00000000e+00,1.
	00000000e+00},prob(percent)="100",prob+-sd="100+-0"]:2.933142e-01[&length_mean=3.02
	332746e-01,length_median=2.93314200e-01,length_95%HPD={1.35150900e-01,4.90063400e-0
	1}],(64[&prob=1.00000000e+00,prob_stddev=0.00000000e+00,prob_range={1.00000000e+00,
	1.00000000e+00},prob(percent)="100",prob+-sd="100+-0"]:7.375446e-01[&length_mean=7.
	49766863e-01,length_median=7.37544600e-01,length_95%HPD={4.87033500e-01,1.03510200e
	+00}],((65[&prob=1.00000000e+00,prob_stddev=0.00000000e+00,prob_range={1.00000000e+
	00,1.00000000e+00},prob(percent)="100",prob+-sd="100+-0"]:1.517695e-01[&length_mean
	=1.55281672e-01,length_median=1.51769500e-01,length_95%HPD={7.91928000e-02,2.351152
	00e-01}],67[&prob=1.00000000e+00,prob_stddev=0.00000000e+00,prob_range={1.00000000e
	+00,1.00000000e+00},prob(percent)="100",prob+-sd="100+-0"]:7.045540e-02[&length_mea
	n=7.38405983e-02,length_median=7.04554000e-02,length_95%HPD={2.71519600e-02,1.27515
	100e-01}])[&prob=9.66671111e-01,prob_stddev=7.54146680e-03,prob_range={9.61338488e-
```

01,9.72003733e-01},prob(percent)="97",prob+-sd="97+-1"]:7.652705e-02[&length_mean=7
.94147264e-02,length_median=7.65270500e-02,length_95%HPD={1.76116800e-02,1.43716200
e-01}],66[&prob=1.00000000e+00,prob_stddev=0.00000000e+00,prob_range={1.00000000e+0
0,1.00000000e+00},prob(percent)="100",prob+-sd="100+-0"]:3.808592e-02[&length_mean=
4.31561939e-02,length_median=3.80859200e-02,length_95%HPD={3.65835900e-05,9.4825630
0e-02}])[&prob=9.19144114e-01,prob_stddev=1.41402503e-03,prob_range={9.18144247e-01
,9.20143981e-01},prob(percent)="92",prob+-sd="92+-0"]:1.764022e-01[&length_mean=1.8
3102069e-01,length_median=1.76402200e-01,length_95%HPD={3.01372200e-02,3.42212700e-
01}])[&prob=9.99200107e-01,prob_stddev=0.00000000e+00,prob_range={9.99200107e-01,9.
99200107e-01},prob(percent)="100",prob+-sd="100+-0"]:3.075900e-01[&length_mean=3.15
035281e-01,length_median=3.07590000e-01,length_95%HPD={1.06562300e-01,5.28201200e-0
1}])[&prob=9.92667644e-01,prob_stddev=1.50829336e-03,prob_range={9.91601120e-01,9.9
3734169e-01},prob(percent)="99",prob+-sd="99+-0"]:2.522050e-01[&length_mean=2.63404
201e-01,length_median=2.52205000e-01,length_95%HPD={7.17186000e-02,4.71599300e-01}]
)[&prob=5.84055459e-01,prob_stddev=2.58672311e-01,prob_range={4.01146514e-01,7.6696
4405e-01},prob(percent)="58",prob+-sd="58+-26"]:4.443461e-01[&length_mean=4.4701910
0e-01,length_median=4.44346100e-01,length_95%HPD={8.91424100e-02,7.81727500e-01}],(
(((((((((2[&prob=1.00000000e+00,prob_stddev=0.00000000e+00,prob_range={1.00000000e
+00,1.00000000e+00},prob(percent)="100",prob+-sd="100+-0"]:7.670868e-01[&length_mea
n=8.01853341e-01,length_median=7.67086800e-01,length_95%HPD={3.46428900e-01,1.34199
700e+00}],3[&prob=1.00000000e+00,prob_stddev=0.00000000e+00,prob_range={1.00000000e
+00,1.00000000e+00},prob(percent)="100",prob+-sd="100+-0"]:1.165264e+00[&length_mea
n=1.18036109e+00,length_median=1.16526400e+00,length_95%HPD={6.56424800e-01,1.69583
400e+00}])[&prob=9.99866684e-01,prob_stddev=0.00000000e+00,prob_range={9.99866684e-
01,9.99866684e-01},prob(percent)="100",prob+-sd="100+-0"]:7.519581e-01[&length_mean
=7.80265637e-01,length_median=7.51958100e-01,length_95%HPD={2.90862300e-01,1.263496
00e+00}],(4[&prob=1.00000000e+00,prob_stddev=0.00000000e+00,prob_range={1.00000000e
+00,1.00000000e+00},prob(percent)="100",prob+-sd="100+-0"]:1.409007e+00[&length_mea
n=1.43435372e+00,length_median=1.40900700e+00,length_95%HPD={8.61783400e-01,2.09708
400e+00}],5[&prob=1.00000000e+00,prob_stddev=0.00000000e+00,prob_range={1.00000000e
+00,1.00000000e+00},prob(percent)="100",prob+-sd="100+-0"]:1.399489e+00[&length_mea
n=1.42702530e+00,length_median=1.39948900e+00,length_95%HPD={8.11859300e-01,2.04154
800e+00}])[&prob=5.64324757e-01,prob_stddev=1.18023955e-01,prob_range={4.80869217e-
01,6.47780296e-01},prob(percent)="56",prob+-sd="56+-12"]:3.365964e-01[&length_mean=
3.76002699e-01,length_median=3.36596400e-01,length_95%HPD={4.89389100e-04,8.2527560
0e-01}])[&prob=9.25609919e-01,prob_stddev=8.05051581e-02,prob_range={8.68684175e-01
,9.82535662e-01},prob(percent)="93",prob+-sd="93+-8"]:7.487390e-01[&length_mean=7.6
5672477e-01,length_median=7.48739000e-01,length_95%HPD={2.72923800e-01,1.29352500e+
00}],37[&prob=1.00000000e+00,prob_stddev=0.00000000e+00,prob_range={1.00000000e+00,
1.00000000e+00},prob(percent)="100",prob+-sd="100+-0"]:1.195153e+00[&length_mean=1.
21179174e+00,length_median=1.19515300e+00,length_95%HPD={5.60370200e-01,1.87824100e
+00}])[&prob=4.09878683e-01,prob_stddev=2.15874487e-02,prob_range={3.94614051e-01,4
.25143314e-01},prob(percent)="41",prob+-sd="41+-2"]:4.348567e-01[&length_mean=4.527
97953e-01,length_median=4.34856700e-01,length_95%HPD={8.69784000e-02,8.75323700e-01
}],(((((11[&prob=1.00000000e+00,prob_stddev=0.00000000e+00,prob_range={1.00000000e+
00,1.00000000e+00},prob(percent)="100",prob+-sd="100+-0"]:1.654670e+00[&length_mean
=1.69201117e+00,length_median=1.65467000e+00,length_95%HPD={1.11964900e+00,2.334244
00e+00}],23[&prob=1.00000000e+00,prob_stddev=0.00000000e+00,prob_range={1.00000000e
+00,1.00000000e+00},prob(percent)="100",prob+-sd="100+-0"]:7.489540e-01[&length_mea
n=7.70243983e-01,length_median=7.48954000e-01,length_95%HPD={3.99826900e-01,1.15952
600e+00}])[&prob=8.84815358e-01,prob_stddev=1.13122002e-02,prob_range={8.76816424e-
01,8.92814291e-01},prob(percent)="88",prob+-sd="88+-1"]:3.221853e-01[&length_mean=3
.36461106e-01,length_median=3.22185300e-01,length_95%HPD={4.76967300e-02,6.48727800
e-01}],(24[&prob=1.00000000e+00,prob_stddev=0.00000000e+00,prob_range={1.00000000e+
00,1.00000000e+00},prob(percent)="100",prob+-sd="100+-0"]:8.541422e-01[&length_mean
=8.70565198e-01,length_median=8.54142200e-01,length_95%HPD={5.76105800e-01,1.200144
00e+00}],(25[&prob=1.00000000e+00,prob_stddev=0.00000000e+00,prob_range={1.00000000
e+00,1.00000000e+00},prob(percent)="100",prob+-sd="100+-0"]:1.550570e-01[&length_me
an=1.60758688e-01,length_median=1.55057000e-01,length_95%HPD={6.46402000e-02,2.6334
3000e-01}],26[&prob=1.00000000e+00,prob_stddev=0.00000000e+00,prob_range={1.0000000
0e+00,1.00000000e+00},prob(percent)="100",prob+-sd="100+-0"]:1.339859e-01[&length_m
ean=1.39842708e-01,length_median=1.33985900e-01,length_95%HPD={5.12939700e-02,2.315
18300e-01}])[&prob=9.97933609e-01,prob_stddev=8.48415015e-04,prob_range={9.97333689
e-01,9.98533529e-01},prob(percent)="100",prob+-sd="100+-0"]:2.117845e-01[&length_me
an=2.18849712e-01,length_median=2.11784500e-01,length_95%HPD={6.01628400e-02,3.9403
4800e-01}])[&prob=1.00000000e+00,prob_stddev=0.00000000e+00,prob_range={1.00000000e
+00,1.00000000e+00},prob(percent)="100",prob+-sd="100+-0"]:3.580939e-01[&length_mea
n=3.66318150e-01,length_median=3.58093900e-01,length_95%HPD={1.74559800e-01,5.92062
900e-01}])[&prob=3.46153846e-01,prob_stddev=7.04184463e-02,prob_range={2.96360485e-
01,3.95947207e-01},prob(percent)="35",prob+-sd="35+-7"]:9.707950e-02[&length_mean=1
.10797773e-01,length_median=9.70795000e-02,length_95%HPD={7.40074300e-05,2.50404800

0000000e+00},prob(percent)="100",prob+-sd="100+-0"]:8.893156e-01[&length_mean=9.056
91518e-01,length_median=8.89315600e-01,length_95%HPD={5.87360000e-01,1.26427100e+00
}])[&prob=8.74016798e-01,prob_stddev=2.60180605e-02,prob_range={8.55619251e-01,8.92
414345e-01},prob(percent)="87",prob+-sd="87+-3"]:2.473064e-01[&length_mean=2.669314
43e-01,length_median=2.47306400e-01,length_95%HPD={1.01178800e-02,5.46827700e-01}])
[&prob=1.58978803e-01,prob_stddev=8.38045499e-02,prob_range={9.97200373e-02,2.18237
568e-01},prob(percent)="16",prob+-sd="16+-8"]:1.807196e-01[&length_mean=1.93034394e
-01,length_median=1.80719600e-01,length_95%HPD={8.27741100e-04,3.85676400e-01}],(((
(8[&prob=1.00000000e+00,prob_stddev=0.00000000e+00,prob_range={1.00000000e+00,1.000
00000e+00},prob(percent)="100",prob+-sd="100+-0"]:1.538077e+00[&length_mean=1.56879
001e+00,length_median=1.53807700e+00,length_95%HPD={9.91987000e-01,2.17017600e+00}]
,13[&prob=1.00000000e+00,prob_stddev=0.00000000e+00,prob_range={1.00000000e+00,1.00
000000e+00},prob(percent)="100",prob+-sd="100+-0"]:1.188172e+00[&length_mean=1.2126
7308e+00,length_median=1.18817200e+00,length_95%HPD={7.48054300e-01,1.75666400e+00}
])[&prob=9.49340088e-01,prob_stddev=2.82805005e-03,prob_range={9.47340355e-01,9.513
39821e-01},prob(percent)="95",prob+-sd="95+-0"]:3.373802e-01[&length_mean=3.5781832
1e-01,length_median=3.37380200e-01,length_95%HPD={7.32109100e-02,6.96500500e-01}],(
(17[&prob=1.00000000e+00,prob_stddev=0.00000000e+00,prob_range={1.00000000e+00,1.00
000000e+00},prob(percent)="100",prob+-sd="100+-0"]:7.339266e-01[&length_mean=7.5040
3305e-01,length_median=7.33926600e-01,length_95%HPD={4.28237600e-01,1.13163500e+00}
],18[&prob=1.00000000e+00,prob_stddev=0.00000000e+00,prob_range={1.00000000e+00,1.0
0000000e+00},prob(percent)="100",prob+-sd="100+-0"]:7.187776e-01[&length_mean=7.282
09061e-01,length_median=7.18777600e-01,length_95%HPD={4.05185100e-01,1.06295700e+00
}])[&prob=1.00000000e+00,prob_stddev=0.00000000e+00,prob_range={1.00000000e+00,1.00
000000e+00},prob(percent)="100",prob+-sd="100+-0"]:7.381643e-01[&length_mean=7.4934
5064e-01,length_median=7.38164300e-01,length_95%HPD={4.05192900e-01,1.11554400e+00}
],(49[&prob=1.00000000e+00,prob_stddev=0.00000000e+00,prob_range={1.00000000e+00,1.
00000000e+00},prob(percent)="100",prob+-sd="100+-0"]:5.878779e-01[&length_mean=6.04
091330e-01,length_median=5.87877900e-01,length_95%HPD={3.30339600e-01,9.20687000e-0
1}],50[&prob=1.00000000e+00,prob_stddev=0.00000000e+00,prob_range={1.00000000e+00,1
.00000000e+00},prob(percent)="100",prob+-sd="100+-0"]:5.478427e-01[&length_mean=5.5
6004672e-01,length_median=5.47842700e-01,length_95%HPD={2.86107300e-01,8.20246800e-
01}])[&prob=1.00000000e+00,prob_stddev=0.00000000e+00,prob_range={1.00000000e+00,1.
00000000e+00},prob(percent)="100",prob+-sd="100+-0"]:7.014197e-01[&length_mean=7.12
877398e-01,length_median=7.01419700e-01,length_95%HPD={3.54701000e-01,1.08600900e+0
0}])[&prob=7.86428476e-01,prob_stddev=1.11236635e-01,prob_range={7.07772297e-01,8.6
5084655e-01},prob(percent)="79",prob+-sd="79+-11"]:2.186007e-01[&length_mean=2.3956
9995e-01,length_median=2.18600700e-01,length_95%HPD={1.93237600e-05,5.20349100e-01}
])[&prob=4.06479136e-01,prob_stddev=4.35519708e-02,prob_range={3.75683242e-01,4.372
75030e-01},prob(percent)="41",prob+-sd="41+-4"]:1.949683e-01[&length_mean=2.1108886
0e-01,length_median=1.94968300e-01,length_95%HPD={8.52405400e-03,4.30373400e-01}],1
2[&prob=1.00000000e+00,prob_stddev=0.00000000e+00,prob_range={1.00000000e+00,1.0000
0000e+00},prob(percent)="100",prob+-sd="100+-0"]:1.742835e+00[&length_mean=1.798685
63e+00,length_median=1.74283500e+00,length_95%HPD={1.12683900e+00,2.60510500e+00}])
[&prob=3.51219837e-01,prob_stddev=2.70078780e-01,prob_range={1.60245301e-01,5.42194
374e-01},prob(percent)="35",prob+-sd="35+-27"]:1.329276e-01[&length_mean=1.44804195
e-01,length_median=1.32927600e-01,length_95%HPD={4.14617200e-04,3.03630000e-01}],((
73[&prob=1.00000000e+00,prob_stddev=0.00000000e+00,prob_range={1.00000000e+00,1.000
00000e+00},prob(percent)="100",prob+-sd="100+-0"]:2.154148e-01[&length_mean=2.20272
090e-01,length_median=2.15414800e-01,length_95%HPD={8.65477700e-02,3.58011800e-01}]
,74[&prob=1.00000000e+00,prob_stddev=0.00000000e+00,prob_range={1.00000000e+00,1.00
000000e+00},prob(percent)="100",prob+-sd="100+-0"]:2.291229e-01[&length_mean=2.3644
3554e-01,length_median=2.29122900e-01,length_95%HPD={1.08589600e-01,3.81920200e-01}
])[&prob=1.00000000e+00,prob_stddev=0.00000000e+00,prob_range={1.00000000e+00,1.000
00000e+00},prob(percent)="100",prob+-sd="100+-0"]:5.643877e-01[&length_mean=5.75451
926e-01,length_median=5.64387700e-01,length_95%HPD={2.89234000e-01,8.78301900e-01}]
,((80[&prob=1.00000000e+00,prob_stddev=0.00000000e+00,prob_range={1.00000000e+00,1.
00000000e+00},prob(percent)="100",prob+-sd="100+-0"]:8.666935e-02[&length_mean=8.93
483203e-02,length_median=8.66693500e-02,length_95%HPD={1.54287800e-02,1.69057900e-0
1}],81[&prob=1.00000000e+00,prob_stddev=0.00000000e+00,prob_range={1.00000000e+00,1
.00000000e+00},prob(percent)="100",prob+-sd="100+-0"]:1.399032e-01[&length_mean=1.4
2038968e-01,length_median=1.39903200e-01,length_95%HPD={5.65272500e-02,2.31937900e-
01}])[&prob=9.76936408e-01,prob_stddev=1.88536670e-03,prob_range={9.75603253e-01,9.
78269564e-01},prob(percent)="98",prob+-sd="98+-0"]:1.949633e-01[&length_mean=2.0126
5135e-01,length_median=1.94963300e-01,length_95%HPD={3.05759400e-02,3.70978500e-01}
],(82[&prob=1.00000000e+00,prob_stddev=0.00000000e+00,prob_range={1.00000000e+00,1.
00000000e+00},prob(percent)="100",prob+-sd="100+-0"]:1.614357e-01[&length_mean=1.64
929818e-01,length_median=1.61435700e-01,length_95%HPD={7.80648400e-02,2.50386100e-0
1}],83[&prob=1.00000000e+00,prob_stddev=0.00000000e+00,prob_range={1.00000000e+00,1
.00000000e+00},prob(percent)="100",prob+-sd="100+-0"]:3.783849e-02[&length_mean=4.4
0470388e-02,length_median=3.78384900e-02,length_95%HPD={3.20517600e-06,1.05696600e-

00000e+00},prob(percent)="100",prob+-sd="100+-0"]:4.830129e-01[&length_mean=4.88500
831e-01,length_median=4.83012900e-01,length_95%HPD={2.20724000e-01,7.67827300e-01}]
,(((((95[&prob=1.00000000e+00,prob_stddev=0.00000000e+00,prob_range={1.00000000e+00
,1.00000000e+00},prob(percent)="100",prob+-sd="100+-0"]:8.447814e-01[&length_mean=8
.62280045e-01,length_median=8.44781400e-01,length_95%HPD={5.65126500e-01,1.19354600
e+00}],(156[&prob=1.00000000e+00,prob_stddev=0.00000000e+00,prob_range={1.00000000e
+00,1.00000000e+00},prob(percent)="100",prob+-sd="100+-0"]:5.298694e-01[&length_mea
n=5.34831371e-01,length_median=5.29869400e-01,length_95%HPD={3.23927900e-01,7.50962
200e-01}],(177[&prob=1.00000000e+00,prob_stddev=0.00000000e+00,prob_range={1.000000
00e+00,1.00000000e+00},prob(percent)="100",prob+-sd="100+-0"]:2.230402e-01[&length_
mean=2.28794009e-01,length_median=2.23040200e-01,length_95%HPD={1.10625500e-01,3.54
442200e-01}],178[&prob=1.00000000e+00,prob_stddev=0.00000000e+00,prob_range={1.0000
0000e+00,1.00000000e+00},prob(percent)="100",prob+-sd="100+-0"]:3.449204e-01[&lengt
h_mean=3.49721308e-01,length_median=3.44920400e-01,length_95%HPD={2.08430300e-01,5.
11675300e-01}])[&prob=9.95267298e-01,prob_stddev=3.48792840e-03,prob_range={9.92800
960e-01,9.97733636e-01},prob(percent)="100",prob+-sd="100+-0"]:2.089280e-01[&length
_mean=2.15544053e-01,length_median=2.08928000e-01,length_95%HPD={7.70502300e-02,3.7
2984100e-01}])[&prob=5.83055593e-01,prob_stddev=2.05599239e-01,prob_range={4.376749
77e-01,7.28436209e-01},prob(percent)="58",prob+-sd="58+-21"]:1.361482e-01[&length_m
ean=1.41283585e-01,length_median=1.36148200e-01,length_95%HPD={2.26589500e-02,2.620
95800e-01}])[&prob=5.08465538e-01,prob_stddev=2.76960368e-01,prob_range={3.12624983
e-01,7.04306093e-01},prob(percent)="51",prob+-sd="51+-28"]:1.161467e-01[&length_mea
n=1.23991826e-01,length_median=1.16146700e-01,length_95%HPD={8.27356300e-03,2.42042
500e-01}],(172[&prob=1.00000000e+00,prob_stddev=0.00000000e+00,prob_range={1.000000
00e+00,1.00000000e+00},prob(percent)="100",prob+-sd="100+-0"]:9.929069e-02[&length_
mean=1.02155793e-01,length_median=9.92906900e-02,length_95%HPD={3.63405400e-02,1.76
823600e-01}],173[&prob=1.00000000e+00,prob_stddev=0.00000000e+00,prob_range={1.0000
0000e+00,1.00000000e+00},prob(percent)="100",prob+-sd="100+-0"]:8.252906e-02[&lengt
h_mean=8.51367891e-02,length_median=8.25290600e-02,length_95%HPD={2.06117400e-02,1.
52775200e-01}])[&prob=1.00000000e+00,prob_stddev=0.00000000e+00,prob_range={1.00000
000e+00,1.00000000e+00},prob(percent)="100",prob+-sd="100+-0"]:2.862087e-01[&length
_mean=2.90752367e-01,length_median=2.86208700e-01,length_95%HPD={1.51458600e-01,4.3
4341600e-01}])[&prob=4.67604319e-01,prob_stddev=2.61971703e-01,prob_range={2.823623
52e-01,6.52846287e-01},prob(percent)="47",prob+-sd="47+-26"]:7.589181e-02[&length_m
ean=8.36170437e-02,length_median=7.58918100e-02,length_95%HPD={9.40601500e-03,1.708
95100e-01}],(((154[&prob=1.00000000e+00,prob_stddev=0.00000000e+00,prob_range={1.00
000000e+00,1.00000000e+00},prob(percent)="100",prob+-sd="100+-0"]:5.023529e-01[&len
gth_mean=5.12149582e-01,length_median=5.02352900e-01,length_95%HPD={2.95846700e-01,
7.17195200e-01}],155[&prob=1.00000000e+00,prob_stddev=0.00000000e+00,prob_range={1.
00000000e+00,1.00000000e+00},prob(percent)="100",prob+-sd="100+-0"]:3.124316e-01[&l
ength_mean=3.20896132e-01,length_median=3.12431600e-01,length_95%HPD={1.53577600e-0
1,5.01350400e-01}])[&prob=1.00000000e+00,prob_stddev=0.00000000e+00,prob_range={1.0
0000000e+00,1.00000000e+00},prob(percent)="100",prob+-sd="100+-0"]:3.741761e-01[&le
ngth_mean=3.82130439e-01,length_median=3.74176100e-01,length_95%HPD={1.97431200e-01
,5.81354400e-01}],174[&prob=1.00000000e+00,prob_stddev=0.00000000e+00,prob_range={1
.00000000e+00,1.00000000e+00},prob(percent)="100",prob+-sd="100+-0"]:2.295846e-01[&
length_mean=2.35364680e-01,length_median=2.29584600e-01,length_95%HPD={1.08882700e-
01,3.74607600e-01}])[&prob=9.99066791e-01,prob_stddev=0.00000000e+00,prob_range={9.
99066791e-01,9.99066791e-01},prob(percent)="100",prob+-sd="100+-0"]:1.755237e-01[&l
ength_mean=1.81397464e-01,length_median=1.75523700e-01,length_95%HPD={6.44488100e-0
2,3.14625500e-01}],(175[&prob=1.00000000e+00,prob_stddev=0.00000000e+00,prob_range=
{1.00000000e+00,1.00000000e+00},prob(percent)="100",prob+-sd="100+-0"]:2.587540e-02
[&length_mean=2.97462051e-02,length_median=2.58754000e-02,length_95%HPD={4.39295600
e-06,6.93574000e-02}],176[&prob=1.00000000e+00,prob_stddev=0.00000000e+00,prob_rang
e={1.00000000e+00,1.00000000e+00},prob(percent)="100",prob+-sd="100+-0"]:5.033358e-
02[&length_mean=5.22324650e-02,length_median=5.03335800e-02,length_95%HPD={6.112662
00e-03,1.00236200e-01}])[&prob=1.00000000e+00,prob_stddev=0.00000000e+00,prob_range
={1.00000000e+00,1.00000000e+00},prob(percent)="100",prob+-sd="100+-0"]:2.238035e-0
1[&length_mean=2.28523349e-01,length_median=2.23803500e-01,length_95%HPD={1.1311080
0e-01,3.57089900e-01}])[&prob=4.61471804e-01,prob_stddev=1.80052520e-02,prob_range=
{4.48740168e-01,4.74203440e-01},prob(percent)="46",prob+-sd="46+-2"]:4.738239e-02[&
length_mean=5.49319192e-02,length_median=4.73823900e-02,length_95%HPD={3.81372000e-
05,1.29553500e-01}])[&prob=6.19317424e-01,prob_stddev=3.00056110e-01,prob_range={4.
07145714e-01,8.31489135e-01},prob(percent)="62",prob+-sd="62+-30"]:2.286839e-01[&le
ngth_mean=2.33996798e-01,length_median=2.28683900e-01,length_95%HPD={8.74583900e-02
,3.90422600e-01}],((161[&prob=1.00000000e+00,prob_stddev=0.00000000e+00,prob_range=
{1.00000000e+00,1.00000000e+00},prob(percent)="100",prob+-sd="100+-0"]:1.246098e-01
[&length_mean=1.29829966e-01,length_median=1.24609800e-01,length_95%HPD={3.22271400
e-02,2.39251400e-01}],162[&prob=1.00000000e+00,prob_stddev=0.00000000e+00,prob_rang
e={1.00000000e+00,1.00000000e+00},prob(percent)="100",prob+-sd="100+-0"]:2.105473e-
01[&length_mean=2.14462370e-01,length_median=2.10547300e-01,length_95%HPD={1.019143

0000e+00},prob(percent)="100",prob+-sd="100+-0"]:1.445121e-01[&length_mean=1.482297
90e-01,length_median=1.44512100e-01,length_95%HPD={7.07612400e-02,2.29008600e-01}])
[&prob=9.73136915e-01,prob_stddev=3.29939173e-03,prob_range={9.70803893e-01,9.75469
937e-01},prob(percent)="97",prob+-sd="97+-0"]:8.306591e-02[&length_mean=8.67251894e
-02,length_median=8.30659100e-02,length_95%HPD={1.30685300e-02,1.60572400e-01}])[&p
rob=9.79669377e-01,prob_stddev=2.82805005e-04,prob_range={9.79469404e-01,9.79869351
e-01},prob(percent)="98",prob+-sd="98+-0"]:1.069374e-01[&length_mean=1.13202189e-01
,length_median=1.06937400e-01,length_95%HPD={2.39546700e-02,2.12908100e-01}])[&prob
=1.00000000e+00,prob_stddev=0.00000000e+00,prob_range={1.00000000e+00,1.00000000e+0
0},prob(percent)="100",prob+-sd="100+-0"]:2.862437e-01[&length_mean=2.95298833e-01,
length_median=2.86243700e-01,length_95%HPD={1.46052200e-01,4.70678000e-01}])[&prob=
5.18797494e-01,prob_stddev=3.66703823e-02,prob_range={4.92867618e-01,5.44727370e-01
},prob(percent)="52",prob+-sd="52+-4"]:8.769570e-02[&length_mean=9.79233554e-02,len
gth_median=8.76957000e-02,length_95%HPD={4.63015400e-03,2.07440600e-01}],((181[&pro
b=1.00000000e+00,prob_stddev=0.00000000e+00,prob_range={1.00000000e+00,1.00000000e+
00},prob(percent)="100",prob+-sd="100+-0"]:6.330204e-02[&length_mean=6.59217663e-02
,length_median=6.33020400e-02,length_95%HPD={5.63574400e-03,1.19990500e-01}],(182[&
prob=1.00000000e+00,prob_stddev=0.00000000e+00,prob_range={1.00000000e+00,1.0000000
0e+00},prob(percent)="100",prob+-sd="100+-0"]:7.313419e-03[&length_mean=9.79875387e
-03,length_median=7.31341900e-03,length_95%HPD={4.59691800e-07,2.73883600e-02}],183
[&prob=1.00000000e+00,prob_stddev=0.00000000e+00,prob_range={1.00000000e+00,1.00000
000e+00},prob(percent)="100",prob+-sd="100+-0"]:7.444538e-03[&length_mean=9.8141760
4e-03,length_median=7.44453800e-03,length_95%HPD={7.76380900e-06,2.71488800e-02}])[
&prob=6.57978936e-01,prob_stddev=1.19720786e-02,prob_range={6.49513398e-01,6.664444
74e-01},prob(percent)="66",prob+-sd="66+-1"]:2.566673e-02[&length_mean=3.14293711e-
02,length_median=2.56667300e-02,length_95%HPD={4.90619700e-06,8.32073600e-02}])[&pr
ob=1.00000000e+00,prob_stddev=0.00000000e+00,prob_range={1.00000000e+00,1.00000000e
+00},prob(percent)="100",prob+-sd="100+-0"]:3.291756e-01[&length_mean=3.39278397e-0
1,length_median=3.29175600e-01,length_95%HPD={1.72670100e-01,5.14561200e-01}],(184[
&prob=1.00000000e+00,prob_stddev=0.00000000e+00,prob_range={1.00000000e+00,1.000000
00e+00},prob(percent)="100",prob+-sd="100+-0"]:3.938004e-01[&length_mean=4.01628374
e-01,length_median=3.93800400e-01,length_95%HPD={2.42962800e-01,5.82056900e-01}],18
5[&prob=1.00000000e+00,prob_stddev=0.00000000e+00,prob_range={1.00000000e+00,1.0000
0000e+00},prob(percent)="100",prob+-sd="100+-0"]:2.442999e-01[&length_mean=2.509313
52e-01,length_median=2.44299900e-01,length_95%HPD={1.05677600e-01,3.94473700e-01}])
[&prob=9.99733369e-01,prob_stddev=1.88536670e-04,prob_range={9.99600053e-01,9.99866
684e-01},prob(percent)="100",prob+-sd="100+-0"]:2.455346e-01[&length_mean=2.5256640
6e-01,length_median=2.45534600e-01,length_95%HPD={1.03732800e-01,4.20974300e-01}])[
&prob=1.00000000e+00,prob_stddev=0.00000000e+00,prob_range={1.00000000e+00,1.000000
00e+00},prob(percent)="100",prob+-sd="100+-0"]:2.973551e-01[&length_mean=3.07258148
e-01,length_median=2.97355100e-01,length_95%HPD={1.24938700e-01,4.96830500e-01}])[&
prob=5.43594187e-01,prob_stddev=3.51620890e-02,prob_range={5.18730836e-01,5.6845753
9e-01},prob(percent)="54",prob+-sd="54+-4"]:1.071708e-01[&length_mean=1.16572910e-0
1,length_median=1.07170800e-01,length_95%HPD={5.14114000e-03,2.41737400e-01}],(((((
93[&prob=1.00000000e+00,prob_stddev=0.00000000e+00,prob_range={1.00000000e+00,1.000
00000e+00},prob(percent)="100",prob+-sd="100+-0"]:3.741570e-01[&length_mean=3.84156
614e-01,length_median=3.74157000e-01,length_95%HPD={1.98323600e-01,5.69544400e-01}]
,94[&prob=1.00000000e+00,prob_stddev=0.00000000e+00,prob_range={1.00000000e+00,1.00
000000e+00},prob(percent)="100",prob+-sd="100+-0"]:3.354209e-01[&length_mean=3.4214
0611e-01,length_median=3.35420900e-01,length_95%HPD={1.74281400e-01,5.38882500e-01}
])[&prob=1.00000000e+00,prob_stddev=0.00000000e+00,prob_range={1.00000000e+00,1.000
00000e+00},prob(percent)="100",prob+-sd="100+-0"]:5.864627e-01[&length_mean=5.98270
735e-01,length_median=5.86462700e-01,length_95%HPD={3.29182700e-01,8.61664300e-01}]
,(96[&prob=1.00000000e+00,prob_stddev=0.00000000e+00,prob_range={1.00000000e+00,1.0
0000000e+00},prob(percent)="100",prob+-sd="100+-0"]:2.746202e-01[&length_mean=2.795
26934e-01,length_median=2.74620200e-01,length_95%HPD={1.26813300e-01,4.30140800e-01
}],(97[&prob=1.00000000e+00,prob_stddev=0.00000000e+00,prob_range={1.00000000e+00,1
.00000000e+00},prob(percent)="100",prob+-sd="100+-0"]:8.535449e-02[&length_mean=8.9
1711423e-02,length_median=8.53544900e-02,length_95%HPD={3.26530900e-02,1.56768000e-
01}],98[&prob=1.00000000e+00,prob_stddev=0.00000000e+00,prob_range={1.00000000e+00,
1.00000000e+00},prob(percent)="100",prob+-sd="100+-0"]:3.503750e-02[&length_mean=3.
88065727e-02,length_median=3.50375000e-02,length_95%HPD={5.62699700e-05,8.35461300e
-02}])[&prob=9.99533396e-01,prob_stddev=2.82805005e-04,prob_range={9.99333422e-01,9
.99733369e-01},prob(percent)="100",prob+-sd="100+-0"]:1.902246e-01[&length_mean=1.9
8002362e-01,length_median=1.90224600e-01,length_95%HPD={6.68295800e-02,3.49835500e-
01}])[&prob=1.00000000e+00,prob_stddev=0.00000000e+00,prob_range={1.00000000e+00,1.
00000000e+00},prob(percent)="100",prob+-sd="100+-0"]:4.811060e-01[&length_mean=4.89
635307e-01,length_median=4.81106000e-01,length_95%HPD={2.52172300e-01,7.19625500e-0
1}])[&prob=8.50353286e-01,prob_stddev=2.54524505e-03,prob_range={8.48553526e-01,8.5
2153046e-01},prob(percent)="85",prob+-sd="85+-0"]:1.687153e-01[&length_mean=1.91595
482e-01,length_median=1.68715300e-01,length_95%HPD={1.87118900e-03,4.22239900e-01}]
