# Supplementary material for: Membrane Trafficking Modulation during Entamoeba Encystation
Source: Sci Rep. 2017 Oct 9;7:12854. doi: 10.1038/s41598-017-12875-6 (PMC5634486; doi:10.1038/s41598-017-12875-6)
Supplement: Supplementary file 12 — Tree file [file 41598_2017_12875_MOESM12_ESM.pdf]

((((((((((((((EinRabX35:100.0,EhiRabX35:100.0):100.0,(EhiRaxX12:100.0,
(EhiRabX4:100.0,EinRabX4:100.0):100.0):100.0):35.0,EhiRabX27:100.0):15.0,EhiRAN:100.0):3.00,
((EhiRabX5:100.0,(EinRabX40:100.0,EhiRabX32:100.0):48.0):29.0,(EinRabX3:100.0,
((EhiRabX33:100.0,EhiRabX100:100.0):99.0,EinRabZ10:100.0):100.0):13.0):5.00):3.00,
((((((EhiRabX23:100.0,EinRabX23:100.0):100.0,(((EinRabX22C:100.0,EinRabX22A:100.0):100.0,
EhiRabX22:100.0):98.0,EinRabX22B:100.0):98.0):100.0,((EinRab5A:100.0,EhiRab5:100.0):99.0,
EinRab5B:100.0):100.0):64.0,(((EhiRabN2:100.0,(EhiRabN1:100.0,EinRabN1:100.0):100.0):100.0,
EinRabN2:100.0):100.0,(EinRabZ24:100.0,EinRabZ23:100.0):77.0):40.0):28.0,EinRabZ6:100.0):15.0,
((((((EinRabL1:100.0,EinRabL2:100.0):93.0,EhiRab32a:100.0):93.0,EhiRabL1:100.0):100.0,
(((EinRabX11B:100.0,EinRabX11A:100.0):100.0,EhiRabX11:100.0):100.0,EinRabX11C:100.0):100.0):100.0,
EinRabZ1:100.0):100.0,((((((EinRabX31D:100.0,EinRabX31C:100.0):100.0,EinRabX31B:100.0):83.0,
EinRabX31A:100.0):100.0,EhiRabX31:100.0):100.0,EinRabZ13:100.0):100.0,EhiRab21:100.0):84.0):27.0):10.0,
((((((((EinRab7F:100.0,EhiRab7F:100.0):100.0,(EhiRab7A:100.0,EinRab7A:100.0):100.0):100.0,
(EhiRab7C:100.0,((EinRab7E:100.0,EhiRab7E:100.0):100.0,EinRab7C1:100.0):67.0):100.0):100.0,
(((EhiRab7B:100.0,EinRab7B:100.0):100.0,(EhiRab7I:100.0,EinRab7I:100.0):100.0):84.0,
(((EinRabX2B:100.0,EinRabX2A:100.0):83.0,EhiRabX2:100.0):100.0,EhiRabX36:100.0):83.0):53.0):83.0,
(EinRab7D:100.0,EhiRab7D:100.0):100.0):72.0,(EinRab7G2:100.0,(EinRab7G1:100.0,
EhiRab7G:100.0):100.0):100.0):48.0,(EinRab7H:100.0,EhiRab7H:100.0):100.0):83.0,
(((EinRabX14B:100.0,EinRabX14A:100.0):100.0,EhiRabX14:100.0):100.0,(((EinRab8A:100.0,
(EhiRAB8B:100.0,EinRab8B:100.0):100.0):97.0,EhiRab8A:100.0):68.0,(EhiRabX13:100.0,
EinRabZ9:100.0):60.0):59.0):57.0,EhiRabX24:100.0):49.0):18.0,(((EinRabX25:100.0,
EhiRabX25:100.0):100.0,((EinRabA:100.0,EhiRabA:100.0):99.0,(EinRabH:100.0,
EhiRabH:100.0):100.0):100.0):70.0,((EinRabZ20:100.0,EhiRaxX11:100.0):99.0,(((EhiRaxX14:100.0,
EhiRaxX13:100.0):86.0,(EhiRaxX16:100.0,EhiRaxX15:100.0):100.0):96.0,EhiRabX21:100.0):88.0):63.0):59.0):12.0):2.00,
((((((((EhiRabF1:100.0,EinRabF1:100.0):100.0,(EhiRabF5:100.0,EinRabF5:100.0):100.0):100.0,
((EhiRabP:100.0,EinRabX39:100.0):100.0,((EhiRabX15:100.0,EinRabX15:100.0):100.0,
EinRabZ25:100.0):98.0):79.0):42.0,(((EhiRabF3:100.0,EinRabF3:100.0):100.0,(EinRabF4:100.0,
EhiRabF4:100.0):100.0):88.0,((EhiRabX9:100.0,EinRabX9:100.0):100.0,(EhiRabF2:100.0,
EinRabF2:100.0):100.0):99.0):98.0):37.0,EhiRabX20:100.0):33.0,(EinRabX1A:100.0,
(EhiRabX1:100.0,EinRabX1B:100.0):100.0):100.0):53.0,EhiRabX8:100.0):38.0,((EhiRabM2:100.0,
(EinRabM3:100.0,EhiRabM3:100.0):99.0):83.0,(EinRabM1:100.0,EhiRabM1:100.0):96.0):100.0):19.0,
(((((((EhiRab11D:100.0,EinRab11D:100.0):100.0,(EhiRab11B:100.0,EinRab11B:100.0):100.0):100.0,
((EhiRab11C:100.0,EinRab11C:100.0):100.0,(EhiRab11A:100.0,EinRab11A:100.0):100.0):99.0):100.0,
(EhiRabX16:100.0,EinRabX16:100.0):100.0):90.0,(((EinRab2B:100.0,EhiRab2C:100.0):100.0,
EhiRab2B:100.0):100.0,(EhiRab2A:100.0,EinRab2A:100.0):100.0):100.0):86.0,(EinRabZ5:100.0,
EinRabZ7:100.0):100.0):26.0,(((((EinRabX30:100.0,EhiRabX30:100.0):100.0,((EhiRabP1:100.0,
EhiRabP2:100.0):100.0,EinRabP:100.0):100.0):99.0,EhiRabX18:100.0):76.0,(((EinRabX34A:100.0,
EinRabX34B:100.0):99.0,EhiRabX34:100.0):100.0,((EinRab1B:100.0,EhiRab1B:100.0):100.0,
(EhiRab1A:100.0,EinRab1A:100.0):100.0):86.0):73.0):41.0,(EinRabX12:100.0,EhiRabX12:100.0):98.0):28.0):8.00):3.00):2.00,
((((((((EinRab17A:100.0,EhiRabX17:100.0):100.0,EinRabX17B:100.0):100.0,
EinRabX17C:100.0):100.0,EinRabZ16:100.0):100.0,((EhiRabX10:100.0,EinRabX10:100.0):100.0,
(EinRabZ17:100.0,EhiRaxX7:100.0):100.0):88.0):48.0,(((((EinRabZ26:100.0,(EinRabZ8A:100.0,
EinRabZ8B:100.0):100.0):100.0,((EhiRaxX9:100.0,EinRabZ12:100.0):98.0,(EhiRabX29:100.0,
EinRabX29:100.0):100.0):72.0):72.0,EhiRabX7:100.0):52.0,(EinRabX7:100.0,(EinRabX6:100.0,
EhiRabX6:100.0):98.0):99.0):95.0,EinRabX41:100.0):94.0):33.0,(EhiRaxX10:100.0,

```
EinRabZ15:100.0):94.0):14.0,((((((EinRabC5:100.0,EhiRabC5:100.0):100.0,((((EinRabZ22:100.0,
EinRabZ21A:100.0):100.0,(EinRabZ4A:100.0,EinRabZ4B:100.0):100.0):100.0,(EhiRabC2:100.0,
EinRabC2:100.0):100.0):51.0,(EhiRabC6:100.0,((EhiRabC8:100.0,EinRabC8:100.0):99.0,
(EinRabC7:100.0,EhiRabC7:100.0):100.0):92.0):98.0):33.0):53.0,(EhiRabB:100.0,
EinRabB:100.0):100.0):32.0,(((EinRabZ3:100.0,(EinRabC4:100.0,EhiRabC4:100.0):100.0):64.0,
EinRabZ18:100.0):71.0,(((EinRabZ2A:100.0,EinRabZ2B:100.0):100.0,EinRabC3B:100.0):100.0,
((EhiRabC3:100.0,EinRabC3A:100.0):100.0,(EhiRabC1:100.0,EinRabC1:100.0):100.0):44.0):44.0):85.0):53.0,
((EinRabX19:100.0,EhiRabX19:100.0):81.0,((EhiRabX26:100.0,EinRabX26:100.0):99.0,
EinRabX26B:100.0):100.0):100.0):29.0,((((EhiRabD3:100.0,EinRabD:100.0):100.0,
EhiRabD1:100.0):78.0,EhiRabD2:100.0):100.0,EinRabZ19:100.0):31.0):8.00):2.00):63.0,
((EhiRabI2:100.0,EhiRabI3:100.0):100.0,(EinRabI:100.0,EhiRabI1:100.0):100.0):100.0):90.0,
EinRabZ11:100.0):84.0,(EinRabK5:100.0,EhiRabK5:100.0):100.0):72.0,(EhiRabK4:100.0,
EinRabK4:100.0):100.0):48.0,EhiRabK3:100.0):94.0,(EinRabK2:100.0,(EhiRabK2:100.0,
EinRabZ14:100.0):83.0):91.0):100.0,EhiRabK1:100.0):100.0,EinRabK1:100.0);
```
